# A methodological framework for constructing opioid agonist therapy episodes in administrative health data

**DOI:** 10.1186/s44330-026-00064-9

**Published:** 2026-03-12

**Authors:** Kiana Yazdani, Cassidy Tam, Christopher C. Fisher, Scott D. Emerson, Jason Trigg, Katherine W. Kooij, Mary A. De Vera, Fidel Vila-Rodriguez, Robert S. Hogg, Julio S. G. Montaner, Viviane Dias Lima

**Affiliations:** 1 Epidemiology and Population Health Program, British Columbia Centre for Excellence in HIV/AIDS, 608-1081 Burrard Street, Vancouver, BC V6Z 1Y6 Canada; 2 The University of British Columbia, Vancouver, BC Canada; 3 Simon Fraser University, Burnaby, BC Canada; 4 Arthritis Research Canada, Vancouver, Canada; 5 Spanish Consortium of Biomedical Research in Epidemiology and Public Health, Madrid, Spain

**Keywords:** Opioid agonist therapy, Opioid use disorder, Administrative health data, Medication episode construction, Allen’s relations, Temporal margin, Permissible gap

## Abstract

**Background:**

Opioid Agonist Therapy (OAT) is the most effective intervention to reduce overdose risk, and administrative health data are now increasingly used to study OAT outcomes. However, current methods for constructing OAT episodes rely heavily on fixed permissible gaps and often overlook switches between medications, concurrent therapies, and the temporal complexity of dispensing patterns. We aimed to develop a robust episode-construction framework that more precisely reflects real-world OAT use within administrative health data.

**Methods:**

We analyzed OAT dispensations from people living with HIV in British Columbia (2010–2020). Episodes were constructed using three components: Allen’s interval algebra, temporal margins ($\mathcal{E}$), and drug-specific permissible gaps. Allen’s algebra characterized the temporal relation between two dispensations as *meets* (one starts when another ends), *before* (a positive gap), *overlaps* (partial overlap), *starts* (same start, earlier end), *finishes* (later start, same end), *contains* (one fully encloses another), or *equals* (identical start and end). Temporal margins distinguished short from long overlaps within the same OAT medication and differentiated switches from concurrent therapy when different OATs overlapped. Permissible gaps defined the maximum interruption allowed before episode discontinuation and were mapped onto Allen’s *before* relation. Using this combined framework, we identified monotherapy (single-OAT use), transition (medication switches), multitherapy (concurrent OAT use), and transition-multitherapy episodes.

**Results:**

The *before* relation predominated across all OATs, particularly for methadone (99.43%). The *equals* relation was notably prevalent for buprenorphine (21.46%), slow-release oral morphine (20.87%), and injectable OAT (1.91%). To build monotherapy episodes, we applied $\mathcal{E}$=7 days, while $\mathcal{E}$=14 days differentiated transition from multitherapy episodes. We identified 10,386 episodes: 93.53% monotherapy, 3.72% transition, 2.27% multitherapy, and < 1% transition-multitherapy.

**Conclusion:**

We propose an episode-building framework based on Allen’s relations, temporal margins, and permissible gaps that fine-tune OAT classification in administrative health data. The method is transferable to other settings and populations with suitable parameter adjustments.

**Supplementary Information:**

The online version contains supplementary material available at 10.1186/s44330-026-00064-9.

## Introduction

Opioid Use Disorder (OUD) is a chronic relapsing condition associated with elevated rates of morbidity and mortality [1]. In 2016, British Columbia (BC), Canada, declared a public health emergency due to the alarming increase in overdose deaths driven by a toxic drug supply (the increasingly contaminated and unpredictable unregulated drug market) [2, 3]. Opioid poisoning has since become the leading cause of unnatural death in BC, surpassing the combined number of homicides, suicides, and motor vehicle accidents [4]. Please confirm the inserted city name is correct and amend if necessary.

Despite the complexity of OUD, individuals can achieve sustained long-term recovery with appropriate treatment and support [4]. Opioid Agonist Therapy (OAT) is currently the most effective, evidence-based intervention for OUD, reducing all-cause and overdose mortality [5, 6]. Research additionally suggests that OAT prevents overdoses during the fentanyl crisis, where this potent opioid has entered the illicit drug supply and significantly increases overdose risk [6, 7].

A growing body of OAT-focused research in Canada, especially in BC, now extensively uses administrative health data [6, 8–12]. Administrative health data refer to routinely collected records generated through interactions with the healthcare system, including clinic visits, hospital encounters, laboratory testing, and pharmacy dispensations, primarily for billing, reimbursement, and system management rather than for research purposes [13]. Owing to their population coverage and real-world nature, administrative health datasets provide a powerful and cost-efficient resource for large-scale research [14, 15]. However, because they were not designed for research, they may contain diagnostic under-capture, incomplete information, or coding inconsistencies [13]. Therefore, rigorous methodological approaches are essential to ensure valid inferences and to mitigate data-related bias.

Although administrative health data offer valuable insights into OAT outcomes, current research has two important methodological limitations in constructing OAT treatment episodes. First, most studies do not fully reflect significant programmatic changes in OAT delivery introduced in response to the increasingly toxic drug supply and escalating overdose mortality [4, 9]. For example, OAT treatment options have expanded; in BC, four medications are now approved for treating OUD: methadone, buprenorphine, slow-release oral morphine (SROM), and injectable OAT (iOAT). Less restrictive prescribing practices also allow patients to switch between therapies or use multiple regimens concurrently, depending on their preferences, treatment goals, and disease severity [4, 9]. Yet, most administrative-data studies continue to construct treatment episodes for a single OAT medication, typically methadone or buprenorphine, with limited consideration of additional treatment options, switching patterns, or concurrent OAT use.

Second, episode construction has mainly relied on the permissible gap framework, which defines a continuous episode by specifying the maximum allowable gap between two consecutive dispensations [16]. While this method is practical, assuming fixed gaps oversimplifies the temporal dynamics of OAT dispensations, which may result from patient behaviour, prescriber decisions, programmatic factors, or pharmacy workflow [17]. The permissible gap framework is also limited when dealing with overlapping dispensations. Common practice is either to include the overlap periods or to omit them at the expected end time, failing to account for overlap lengths [16, 18, 19]. Improper management of these overlaps can lead to misclassification of treatment exposure and biased estimates of associations with clinical outcomes [16, 20].

To address these methodological challenges and given the critical importance of OAT outcomes amid the ongoing toxic drug supply, we developed a comprehensive framework for constructing OAT treatment episodes in administrative health data that more precisely reflects real-world OUD care and strengthens OAT-related analyses. This framework integrated three key components: (1) Allen’s interval algebra, a temporal logic system that characterizes the full range of temporal relationships between two dispensations; (2) temporal margins ($\mathcal{E}$), which extend this algebra by differentiating short from long overlaps and enabling the identification of episodes with medication switches or concurrent therapies; and (3) the permissible gap, that determines when an episode is discontinued.

## Methods

### Data sources and study sample

The data were sourced from the *S*eek and *T*reat for *O*ptimal *P*revention of HIV/AIDS (STOP HIV/AIDS) study, an open, bidirectional, population-based longitudinal cohort maintained at the BC Centre for Excellence in HIV/AIDS (BC-CfE) [21]. It includes de-identified, individual-level data on adults ≥ 19 years old with HIV, covering the period from April 1, 1996, to March 31, 2020. The cohort was established by linking three provincial data sources: the BC-CFE Drug Treatment Program [22], the BC Centre for Disease Control HIV/AIDS Surveillance database [23], and administrative health datasets provided by the BC Ministry of Health. Central to our study of OAT was the PharmaNet administrative health database, which records all dispensed prescription medications from BC community and hospital outpatient pharmacies for at-home use [24]. Here, dispensation data refers to records of medications provided to patients, rather than prescriptions written and actual medication use. PharmaNet database excludes inpatient dispensations, over-the-counter medications, and antiretroviral drugs for HIV treatment provided by BC-CfE.

To study OAT using PharmaNet data, we used several core fields. The Drug/Product Identification Number (DIN/PIN) was used to identify the specific OAT medication dispensed. The Date of Service indicated when the medication was provided, while the Days supply reflected the duration for which the dispensation was intended. The Quantity Dispensed represented the total number of OAT units supplied, and the Drug Strength captured the potency of the active ingredients per unit. The daily dose was calculated by multiplying the quantity dispensed by the drug strength and dividing the result by the days supply.

This was not a prespecified secondary analysis of the STOP HIV/AIDS study, but was undertaken to address the complexity observed in OAT data from a prior project [11] and within the context of an ongoing project examining the impact of stimulant use disorder (StUD) on OAT retention, and to help bridge the gap between clinical practice and dispensation data. We analyzed a sample of people living with HIV (PLWH) from the STOP cohort diagnosed with either OUD alone or concurrent OUD and StUD, who received at least one OAT dispensation between April 1, 2010, and March 31, 2020. The case-finding algorithms for OUD and StUD are detailed in Appendix 1.

### Identifying OAT dispensations

The OAT medications examined in this study were methadone, buprenorphine, SROM, and iOAT, identified using their respective DIN/PIN in PharmaNet. Methadone, a long-acting full agonist, is available in several formulations, including liquid, unflavored, and compounded preparations. Buprenorphine, a partial agonist, is available as either buprenorphine/naloxone (Suboxone) tablets or subcutaneous buprenorphine (Sublocade), administered as a monthly injection. The SROM, an intermediate-acting full agonist, is available in a 24-hour formulation. Lastly, iOAT consists of short-acting full agonists, including diacetylmorphine and hydromorphone [4]. Appendix 2 and the complementary Excel file contain the complete definitions and set of DIN/PIN codes for all OAT medications.

### Initial pharmanet data cleaning

Given the variability in OAT dosing (e.g., split doses, variable quantities, multiple same-day pickups) and pharmacy-specific workflow patterns, raw dispensation records sometimes contained errors or inconsistent values requiring correction. These included days supply that were incompatible with the dispensing interval, misaligned service dates, implausible quantity-per-day (QPD) values inconsistent with adjacent records, and large outliers. To address these issues, before constructing treatment episodes, we implemented a sequence of logic-based corrections, adapted from the published methodologies of Pearce et al. [6] and from our previous analysis [11]. We first consolidated split-dose dispensations by summing the quantities for the same day. We then corrected days supply or service dates when they were inconsistent with adjacent records and had inconsistent QPD values. Large QPD deviations were addressed by recalculating the days supply when this produced a clinically plausible pattern, or by adjusting the quantity dispensed when the implied daily dose conflicted with adjacent records. Days supply values that exceeded the time to the next dispensation were reset to match that interval. Appendix 3 provides a detailed description of these cleaning procedures and contrasts them with those used in earlier research. The complementary Excel file illustrates each correction type, and all corresponding code syntax is included in the supplementary materials.

### Episode-construction framework

After completing the initial PharmaNet data cleaning described above, we operationalized three integrated components to construct OAT treatment episodes: Allen’s interval algebra, temporal margins ($\mathcal{E}$), and the permissible gaps.

To capture the complexity of OAT dispensing patterns and the full range of temporal relationships that can stem from irregular medication use, dose adjustments, medication loss, treatment switching or concurrent therapy, and pharmacy workflow inconsistencies (see Appendix 4 for examples), we incorporated Allen’s interval algebra, as adapted for multitherapy medication datasets by Khotimah et al. [19] Allen’s logic defines all possible temporal relations between two intervals, allowing us to characterize each pair of dispensations using one of seven base relations—*meets*, *overlaps*, *before*, *starts*, *finishes*, *contains*, *equals*—and their inverses (13 total) [25, 26]. These relations specify whether one dispensation begins exactly when another ends (*meets*), follows another with a non-zero gap (*before*), overlaps in part (*overlaps*), begins simultaneously but ends earlier (*starts*), ends simultaneously but begins later (*finishes*), fully contains or is fully contained within another (*contains*), or shares identical start and end times (*equals*) (Table 1).

**Table 1:** Allen’s interval algebra

Basic Relations	Description	Inverse Relations	Visual Representation	Temporal Order
X meets Y	X ends exactly where Y starts	Y met by X		X_start_ < X_end_ = Y_start_ < Y_end_
X overlaps Y	X starts before Y, and X ends during Y	Y overlapped by X		X_start_ < Y_start_ < X_end_ < Y_end_
X before Y	X ends before Y starts	Y after X		X_start_ < X_end_ < Y_start_ < Y_end_
X starts Y	X starts at the same time as Y, but ends earlier	Y started by X		X_start_ = Y_start_ < X_end_ < Y_end_
X finishes Y	X ends at the same time as Y, but starts later	Y finished by X		Y_start_ < X_start_< X_end_ = Y_end_
X contains Y	Y starts after X, and ends before X	Y during X		X_start_ < Y_start_ < Y_end_ < X_end_
X equals Y	X and Y start and end at the same time	X equals Y		X_start_ = Y_start_ < X_end_ = Y_end_

Note: Allen’s time interval algebra describes temporal relationships between time intervals based on their chronological order. In our study, a time interval refers to the period from when an OAT is dispensed to the date it is expected to last, as determined by the days supply field in PharmaNet. Table is adapted with modifications from [Mate S, Bürkle T, Kapsner LA, et al. A method for the graphical modeling of relative temporal constraints. J Biomed Inform. 2019; 100:103314. 10.1016/j.jbi.2019.10331] [26]

Although Allen’s relations identify whether an overlap exists, they do not account for the overlap length. To address this, we incorporated temporal margins ($\mathcal{E}$) into the *overlaps* relation. Short overlaps (≤$\mathcal{E}$) were interpreted as continuation of therapy and recoded as *meets*, whereas longer overlaps (>$\mathcal{E}$) were retained to avoid inappropriate inflation of episode length.

The third component, the permissible gap, specifies the maximum allowable interruption between dispensations before an episode is classified as discontinued. This measure defines the discontinuation window and maps onto the *before* relation in Allen’s relations, which captures intervals separated by a positive gap. Establishing this window is critical for measuring OAT outcomes such as treatment retention, adherence, or care discontinuity.

**Fig. 1:**
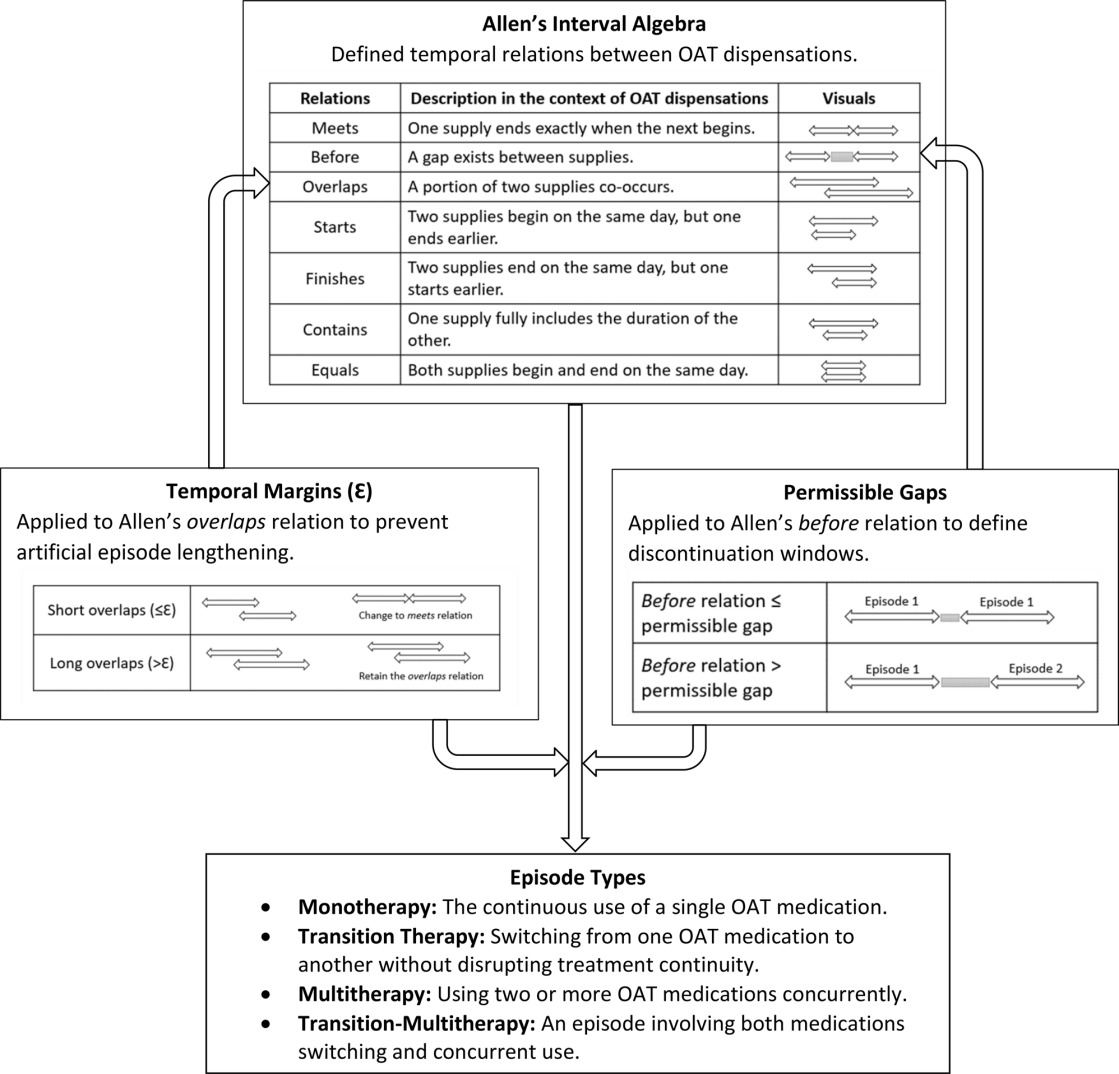
Integrated framework for constructing OAT treatment episodes using Allen’s interval algebra, temporal margins ($\mathcal{E}$), and permissible gaps

Using these three components together, our framework ultimately identified four types of OAT episodes: (1) monotherapy episodes, in which a single OAT medication is dispensed continuously; (2) transition therapy episodes, where an individual switches from one OAT to another while maintaining continuity of care; (3) multitherapy episodes, reflecting concurrent use of two or more OAT medications; and (4) transition-multitherapy, where both switching and concurrent use occur within the same episode. The following sections describe in detail how each component of the framework is applied to identify these episode types. Figure 1 provides a visual schematic of the overall episode-construction framework.

### Constructing monotherapy episodes

Table 2 summarises the procedures used to operationalise Allen’s relations for the construction of monotherapy episodes. Short overlaps between the same OAT dispensations, typically resulting from early refills, routine pharmacy variation, or minor supply carryover, were treated as uninterrupted treatment and recoded as *meets* using the temporal margin ($\mathcal{E}$). In contrast, longer overlaps, which more often signal changes in circumstances such as dose adjustments or lost medication, were left unchanged. To select an appropriate value for $\mathcal{E}$, we compared scenarios with no margin, a 7-day margin, and a 14-day margin by examining the difference between the original Dates of Service and the shifted dates after recoding *overlaps* as *meets*. Episode discontinuation was defined by pairing Allen’s *before* relation with OAT drug-specific permissible gaps, informed by BC OAT clinical guidelines, and the half-lives of OAT medications [4, 9]. Permissible gaps were set at ≤ 6 days for buprenorphine/naloxone, ≤ 5 days for methadone and SROM, and ≤ 3 days for iOAT.

**Table 2:** A description of data manipulation techniques applied to allen’s relations to minimize discrepancies in the OAT data and support the construction of monotherapy episodes

Allen’s Relations	Manipulation Techniques	Descriptions
	Concatenation	Linked consecutive dispensations with no gap to create a continuous single-OAT treatment.
	Temporal Margin ($\mathcal{E}$)	Relaxed Allen’s *overlaps* relation using the concept of $\mathcal{E}$ to differentiate between short and long overlaps under the following conditions: If the overlap duration was ≤ $\mathcal{E}$ days, we shifted the start date of the subsequent dispensation forward and reclassified the temporal relation from *overlaps* to *meets*. If the overlap duration was > $\mathcal{E}$ days, no adjustments were made.
	Permissible Gap	Integrated Allen’s relation with OAT-specific permissible gaps to allow short interruptions within a continuous treatment. If gaps in the *before* relation exceeded the permissible gap, we classified the OAT as discontinued.
	Aggregation	The two dispensations were merged at the common start date value, and the daily dose was added for the nested period.
	Aggregation	The two dispensations were merged at the common end date value, and the daily dose was added for the nested period.
	Aggregation	The two dispensations were merged, using the start and end dates of the dispensation with the larger days supply, and the daily dose was added for the nested period.
	Aggregation	The two dispensations were merged, using the start and end dates from one of the two dispensations. The daily dose was added for the nested period.

Note: Appendix 4, Example 5 provides an example of applying these techniques to PharmaNet dispensations for monotherapy episode construction

### Constructing transition and multitherapy episodes

**Fig. 2:**
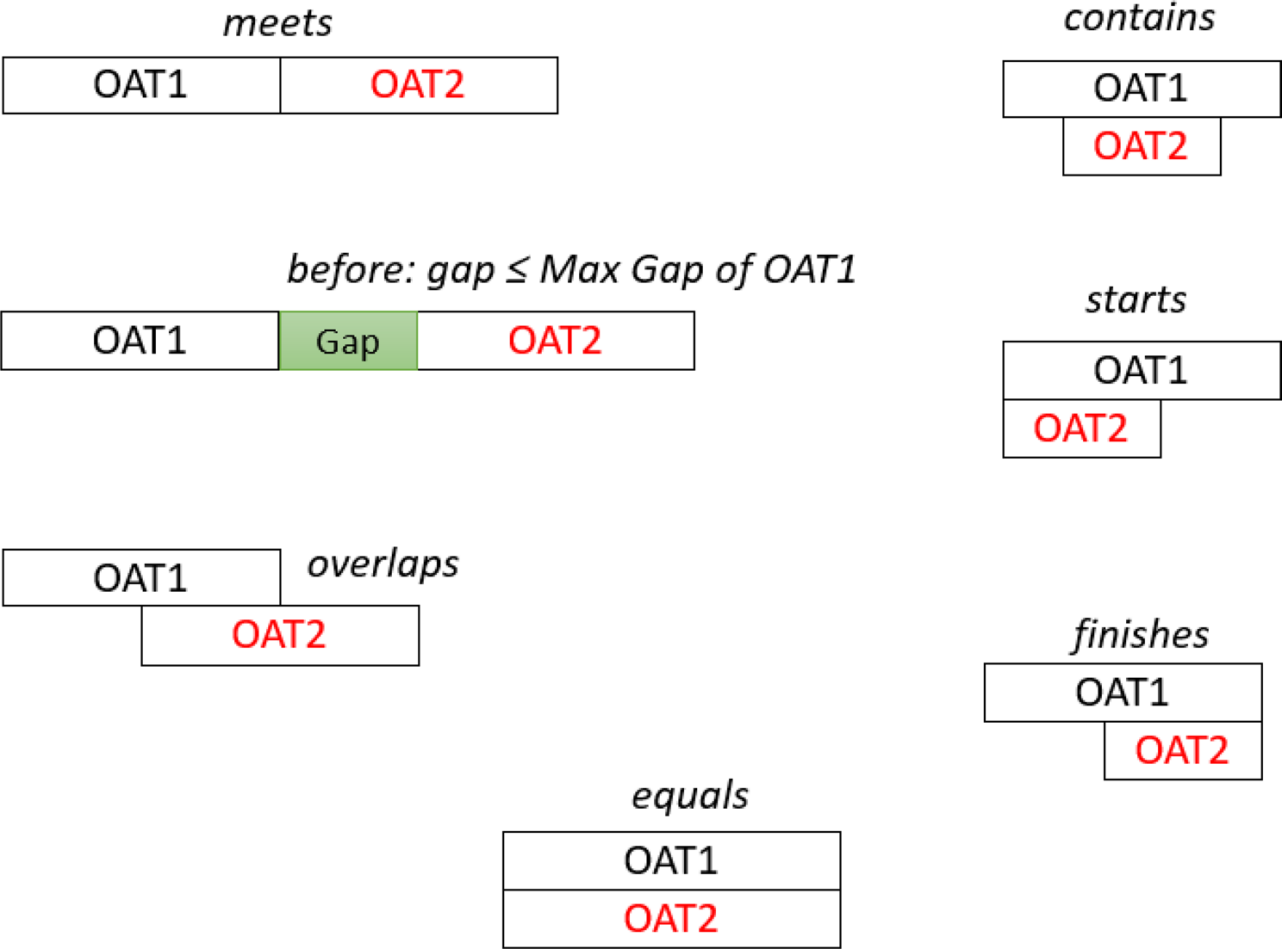
Allen’s relations across different OAT dispensations. The *meet*s relation could occur when switching between full agonists, with no washout period required. The *before* (≤ permissible gap) relation may happen when a washout period is necessary to prevent withdrawal symptoms, especially when transitioning from a full agonist to a partial agonist. The *overlaps* may occur during multitherapy or cross-tapering between OAT medications, particularly when moving from a full agonist to a partial agonist after the new low-dose induction protocol. The *equals*, *contains*, *starts*, or *finishes* relations could occur due to multitherapy or failed transition attempts

Transition therapy refers to a switch from one OAT medication to another while maintaining overall continuity of care. In clinical practice, transitions may occur without a washout period (for example, buprenorphine to methadone), with a brief washout (as previously required for methadone to buprenorphine), or through structured low-dose inductions that introduce short periods of overlap [4]. Within Allen’s relations, these transitions could appear as *meets*, *before* (with a washout period shorter than the drug-specific permissible gap), or as short *overlaps*, respectively (Fig. 2).

Multitherapy occurs when a new OAT is added to the previous one and is distinct from transition therapy. It entails the concurrent use of multiple medications, suggesting that a single treatment is insufficient to address a patient’s condition. In multitherapy, Allen’s relations could be reflected as *overlaps*, *equals*, *contains*, *starts*, or *finishes* (Fig. 2). These relations should remain unchanged to reflect the concurrent use of OAT medications without artificially lengthening the episode.

Therefore, as with constructing monotherapy episodes, a temporal margin ($\mathcal{E}$) is necessary to differentiate between an overlap signifying a transition or multitherapy between different OAT dispensations. Furthermore, to classify *equals*, *contains*, *starts*, or *finishes* relations between different OAT medications as multitherapy, it was necessary to determine the minimum co-dispensation duration for two OAT types, as these relations may also arise during unsuccessful or incomplete transitions between medications.

To define this threshold, we used an approach informed by both empirical data and clinical expertise to distinguish transitions from multitherapy. We identified all periods in which two OAT medications were co-dispensed, summarized their durations using descriptive statistics, and consulted with co-author Christopher C. Fisher, a clinical pharmacist specializing in OAT, to determine the minimum duration needed to categorize an episode as multitherapy. For both transition and multitherapy episodes, discontinuation was defined using the permissible gap corresponding to the last OAT medication dispensed during that episode.

### OAT episodes and hospitalizations

Our data source, PharmaNet, does not include dispensations provided during hospitalizations. Based on inpatient continuity of care guidelines, it is reasonable to assume that OAT continues in some form during patients’ hospital stays [27]. Thus, we assumed OAT continuation if a patient was admitted amid an ongoing OAT episode.

### Statistical analysis

Descriptive statistics were used to characterize OAT dispensations and the constructed episodes. For continuous variables, we calculated means with standard deviations (SD), medians with first and third quartiles (Q1, Q3), and minima, maxima, and modes when relevant. Categorical variables were summarized using frequencies and percentages. All analytic procedures related to episode construction were based on the application of Allen’s interval algebra, including the identification of temporal relations, the use of temporal margins ($\mathcal{E}$) to distinguish short from long overlaps, and the permissible gap to determine episode discontinuation. No inferential modelling was undertaken, as the primary aim was to construct and describe OAT treatment episodes. All analyses were performed using SAS version 9.4 (SAS, Cary, NC, USA).

## Results

Between 2010 and 2020, out of 11,376 PLWH in the STOP cohort, we identified 1,559 (13.70%) individuals with either OUD alone (*n* = 396) or co-occurring OUD-StUD (*n* = 1,163) who were dispensed at least one OAT.

### Initial pharmanet data cleaning

To contextualize the performance of our cleaning procedures, we compared error percentages with earlier PharmaNet-based work. Of 2,094,532 OAT dispensations, 21,977 (1.05%) were flagged and 8,993 (40.92%) corrected. As shown in Appendix 3, this percentage is lower than that reported in a previous BC study of PLWH receiving OAT using the same STOP data source (2.9%) [11] and lower than in a provincial OUD cohort (2.3%) [6]. This difference is expected given our population (PLWH with OUD or OUD-StUD) and refinements to our correction rules, while remaining broadly comparable to prior analyses.

### Constructing monotherapy episodes

The distribution of Allen’s relations across same-type dispensations is summarized in Table 3. The *before* relation dominated across all OAT medications, occurring in > 90% of methadone and iOAT dispensations and 76% of buprenorphine and SROM dispensations. *Equals* relations were most pronounced for buprenorphine (21.46%) and SROM (20.87%), with rare occurrences of other relations (< 1%).

**Table 3:** Allen’s Temporal relations between same-type OAT dispensations in PLWH with OUD, or OUD-StUD

	Methadone (*n* = 1,788,395)	Buprenorphine (*n* = 63,592)	SROM (*n* = 207,960)	iOAT (*n* = 34,585)
Not applicable*	1,331 (0.07)	529 (0.83)	690 (0.33)	175 (0.51)
Meets	3,752 (0.21)	466 (0.73)	1,677 (0.81)	80 (0.23)
Before	1,778,136 (99.43)	48,452 (76.19)	159,516 (76.71)	33,528 (96.94)
Overlaps	406 (0.02)	265 (0.42)	2,069 (0.99)	72 (0.21)
Starts	26 (0.00)	57 (0.09)	140 (0.07)	25 (0.07)
Finishes	113 (0.01)	27 (0.04)	112 (0.05)	11 (0.03)
Contains	540 (0.03)	149 (0.23)	355 (0.17)	34 (0.10)
Equals	4,091 (0.23)	13,647 (21.46)	43,401 (20.87)	660 (1.91)

Note: Data indicate n (%). Frequencies represent dispensation-level counts before episode construction and prior to applying temporal margins and permissible-gap rules.* The not applicable category refers to the first record for participants

Abbreviations: OAT: Opioid Agonist Therapy; SROM: Slow-Release Oral Morphine; iOAT: Injectable Opioid Agonist therapy; PLWH: People living with HIV; OUD: Opioid Use Disorder; StUD: Stimulant Use Disorder

**Fig. 3:**
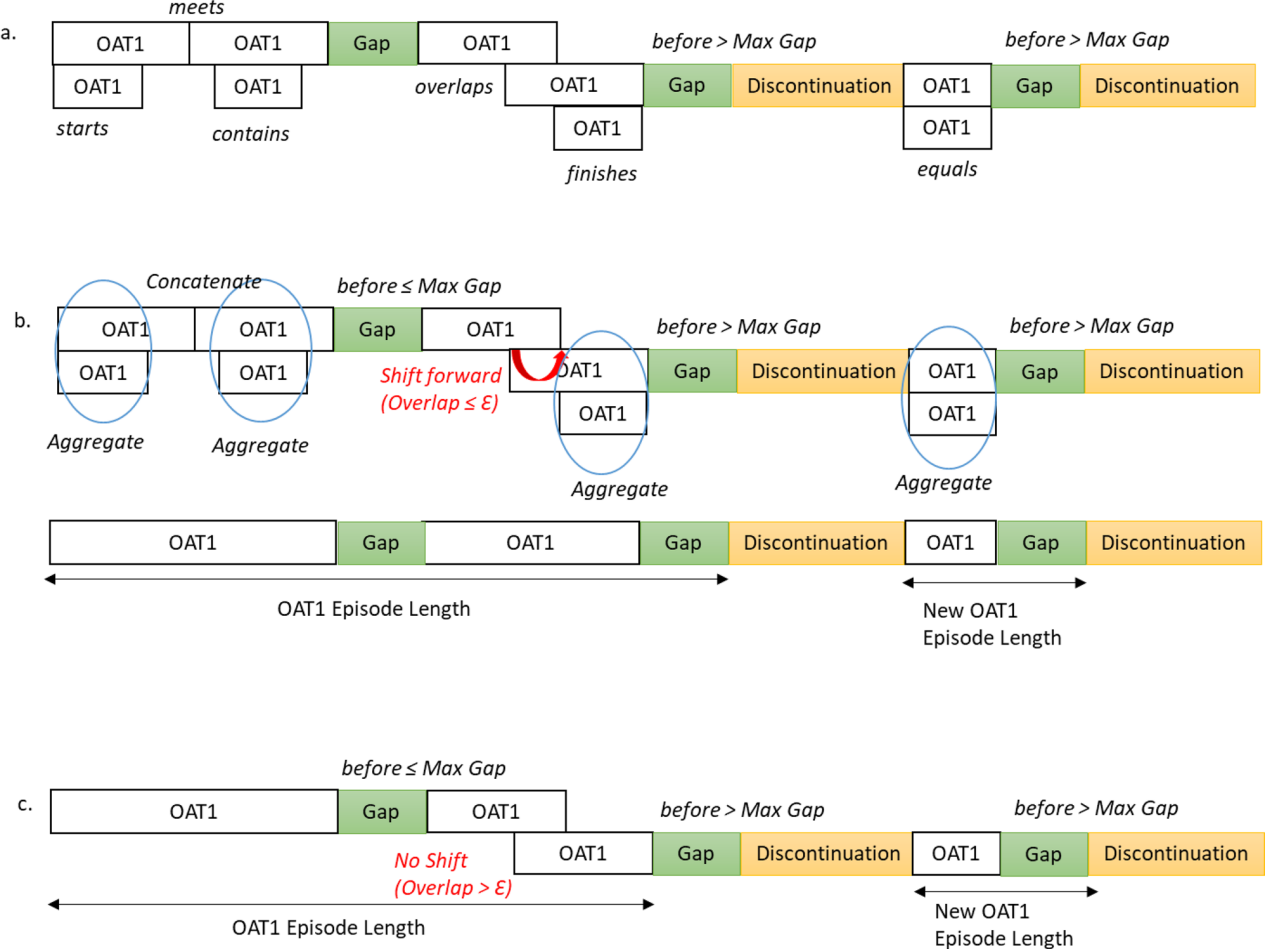
Constructing continuous monotherapy episodes using Allen’s relation, adjusted with temporal margins and permissible gaps. The $\mathcal{E}$ was set to seven days. If the overlap duration was ≤7 days, we shifted the start date of the next dispensation forward, changing the relation from *overlaps* to *meets* (**b**). No adjustments were made for overlaps >7 days (**c**)

Without applying a temporal margin ($\mathcal{E}$), the mode and median differences between original and shifted dates were both zero, indicating minimal shifts for most dispensations. However, large mean and maximum shifts were observed for some SROM dispensations (mean [SD]: 5.44 days [28.03]; max: 695 days), driven by accumulated overlaps. Applying $\mathcal{E}$=7 days reduced this mean difference by approximately 98% to 0.12 days (SD:1.71) (max: 98 days), while $\mathcal{E}$=14 days produced a mean of 0.23 days (SD:3.91) (max: 260 days) (Appendix 5). Therefore, a 7-day margin was chosen to distinguish between short and long overlaps between the same OAT dispensations. Figure 3 illustrates the framework for constructing OAT monotherapy episodes.

### Constructing transition and multitherapy episodes

We identified 2,192 dispensation sequences involving more than one type of OAT, based on co-dispensation periods (Table 4). The most frequently observed pattern was a return to the initial therapy following a period of overlap, with the sequence OAT1→(OAT1 + OAT2)→OAT1 accounting for 1,800 cases (82.12%). A smaller proportion of sequences involved a transition from the initial to the second therapy, represented by OAT1→(OAT1 + OAT2)→OAT2, observed in 328 cases (14.96%). Among the former group, the most common pattern was iOAT→(iOAT+SROM)→iOAT, which occurred in 977 cases (54.28%). In the latter group, the predominant sequence was SROM→(SROM+Methadone)→Methadone, observed in 94 cases (28.66%). Other uncommon sequences with no specific pattern (*n*=64, 2.92%) are detailed in Appendix 6.

**Table 4:** All identified co-dispensation patterns with more than one OAT medication

	Frequency	Median (Q1, Q3)	Mean (SD)	Min	Max
**OAT1** → **(OAT1 + OAT2)** → **OAT1(***n* **= 1,800)**					
iOAT → (iOAT+SROM) → iOAT	977	3 (1, 7)	7.59 (19.58)	1	413
SROM → (SROM + MTD) → SROM	293	4 (2, 9)	13.15 (31.85)	1	277
MTD → (MTD+SROM) → MTD	231	13 (4, 41)	50.85 (110.46)	1	970
iOAT → (iOAT + MTD) → iOAT	101	2 (1, 4)	6.09 (14.97)	1	133
MTD → (MTD+iOAT) → MTD	92	2 (1, 8.5)	18.91 (42.66)	1	217
SROM → (SROM+iOAT) → SROM	77	3 (1, 7)	8.87 (22.37)	1	169
MTD → (MTD + Bup/nal) → MTD	19	4 (2, 8)	5.05 (3.81)	1	16
SROM → (SROM + Bup/nal) → SROM	7	7 (3, 7)	8.29 (9.88)	1	30
iOAT → (iOAT + Bup/nal) → iOAT	< 5	Censored for privacy reasons			
Bup/nal → (Bup/nal+SROM) → Bup/nal	< 5	Censored for privacy reasons			
**OAT1** → **(OAT1 + OAT2)** → **OAT2 (*n*** **= 328)**					
SROM → (SROM + MTD) → MTD	94	11.5 (3,30)	38.29 (98.88)	1	760
MTD → (MTD+SROM) → SROM	77	11 (4, 34)	75.97 (270.48)	1	2,058
SROM → (SROM+iOAT) → iOAT	41	6 (3, 19)	24.93 (50.36)	1	283
iOAT → (iOAT+SROM) → SROM	24	4.5 (2, 12)	10 (15.84)	1	74
MTD → (MTD + Bup/nal) → Bup/nal	24	8 (7, 12.5)	9.79 (6.41)	1	28
MTD → (MTD+iOAT) → iOAT	18	8.5 (2, 25)	56.78 (149.34)	1	637
Bup/nal → (Bup/nal + MTD) → MTD	20	7 (2, 14)	9.85 (10.60)	1	38
iOAT → (iOAT + MTD) → MTD	17	6 (2, 15)	21 (37.91)	1	139
SROM → (SROM + Bup/nal) → Bup/nal	6	7 (2, 7)	7.67 (6.98)	2	21
Bup/nal → (Bup/nal+SROM) → SROM	< 5	Censored for privacy reasons			
Bup/nal → (Bup/nal + iOAT) → iOAT	< 5	Censored for privacy reasons			
iOAT → (Bup/nal + iOAT) → Bup/nal	< 5	Censored for privacy reasons			

Other Sequences with frequencies < 5 (*n* = 64), Appendix 6

Note: The sequences were identified by analyzing the co-dispensation periods. Frequency indicates how often co-dispensations occurred. Other summary statistics provide an overview of the co-dispensation durations

Abbreviations: OAT: Opioid Agonist Therapy; MTD: Methadone; Bup/nal: Buprenorphine/naloxone; SROM: Slow-Release Oral Morphine; iOAT: Injectable Opioid Agonist Therapy; Q1: 25% interquartile; Q3: 75% interquartile; SD: Standard Deviation

**Fig. 4:**
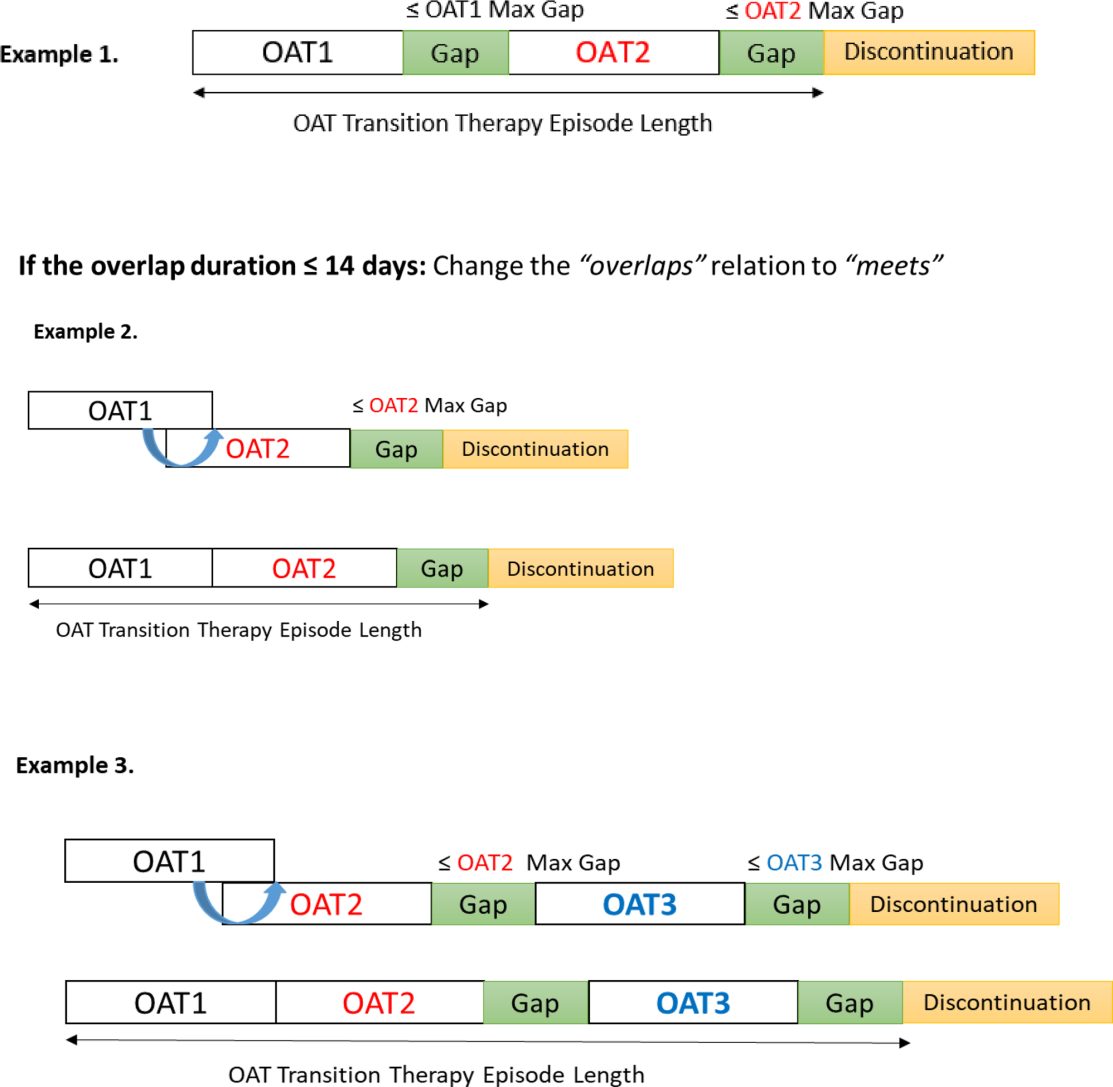
Constructing continuous transition therapy episodes using Allen’s relation, adjusted with temporal margins and permissible gap concepts. The $\mathcal{E}$ was set to 14 days. If Allen’s *overlaps* relation was ≤14 days, we adjusted it to *meets* to build a continuous transition therapy episode (Examples 2 and 3)

The median duration of the co-dispensation periods for all identified sequences was <14 days (Table 4). However, investigating the mean and maximum values showed prolonged co-dispensation periods, suggesting multitherapy before patients either resume their initial OAT or move to an alternative OAT. We chose a 14-day margin to differentiate between transition and multitherapy. Similar to monotherapy episode construction, we applied Allen’s *before* relation with the permissible gap and adjusted the *overlaps* relations using $\mathcal{E}$=14 days. If a new OAT dispensation occurred within the grace period of the previous OAT, we maintained the *before* relation and classified the episode as a transition (Fig. 4, Example 1). If Allen’s *overlaps* relation was ≤14 days, we adjusted it to *meets* to form a transition therapy episode (Fig. 4, Example 2).

**Fig. 5:**
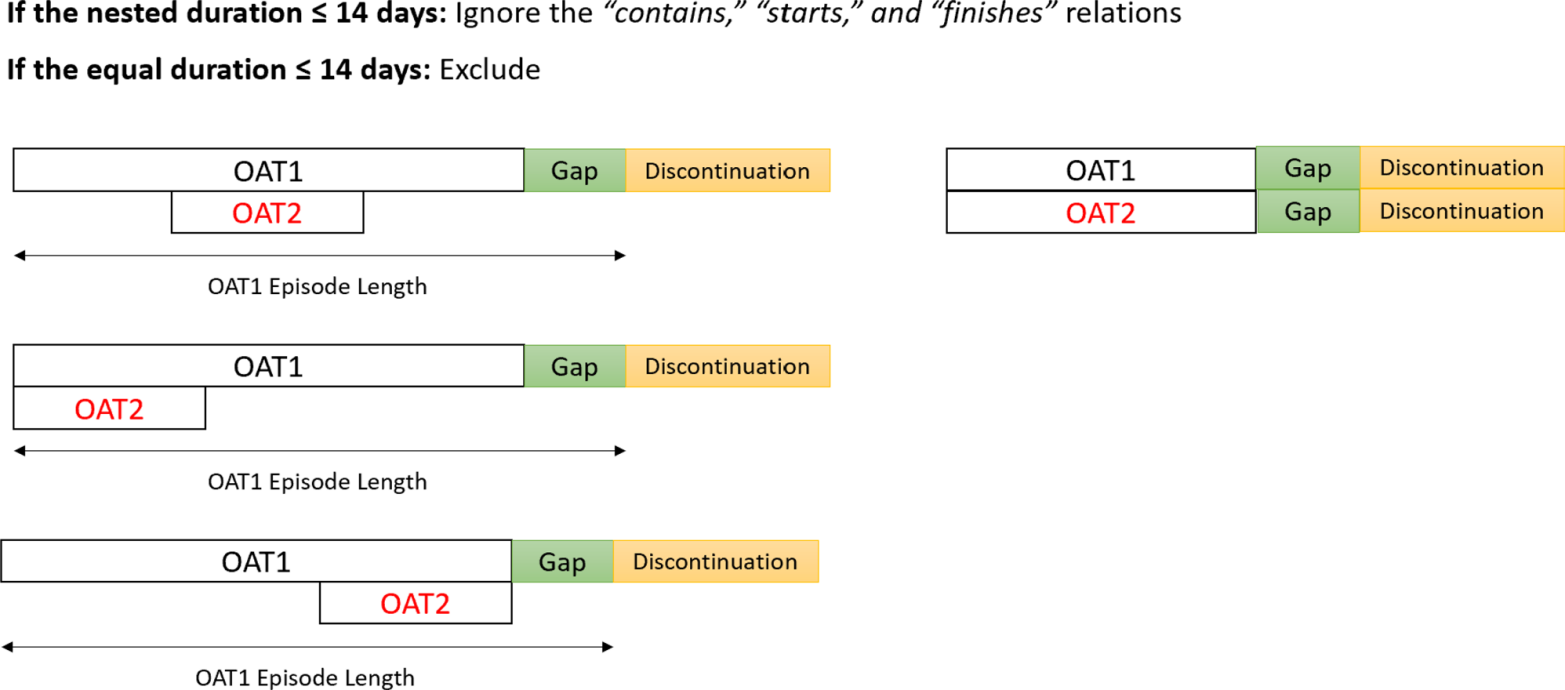
Handling *contains*, *starts*, *finishes*, and *equals* relations between OAT dispensations of multiple types with a co-dispensation period ≤14 days. The *contains*, *starts*, *finishes* relations with co-dispensation periods ≤14 days were ignored, and the episode was assigned to the OAT type encompassing the others. The *equals* relations with the co-dispensation periods ≤14 days were excluded (*n*=15)

**Fig. 6:**
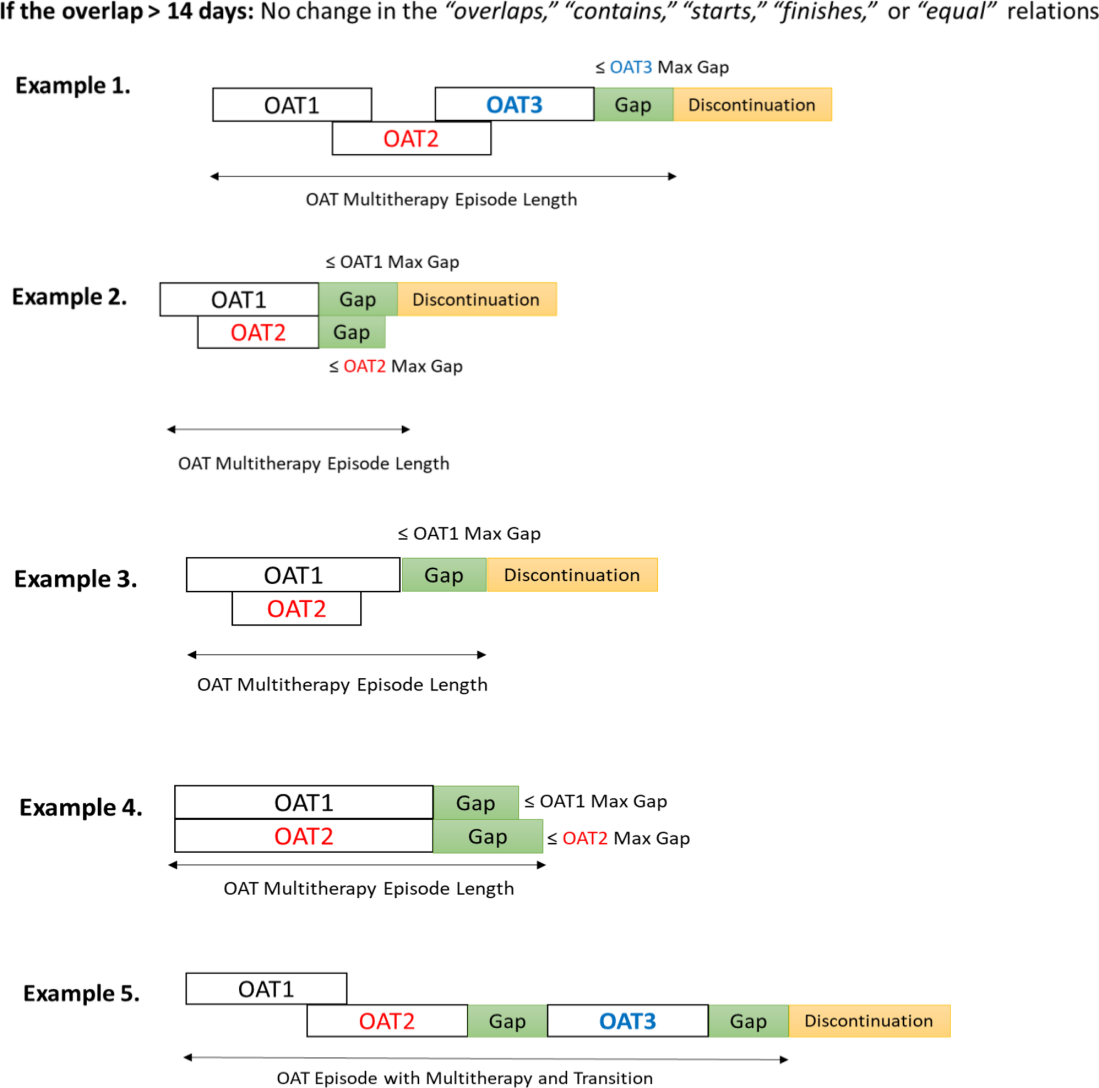
Constructing OAT multitherapy episode using Allen’s relations with a temporal margin of 14 days. If the co-dispensation period in Allen’s *overlaps*, *contains*, *starts*, *finishes*, or *equals* relation was >14 days, the relations remained unchanged, and the episode was categorized as an OAT multitherapy episode. *Example 5* indicates an episode with both multitherapy and transition events

We ignored the *contains*, *starts*, *and finishes* relations with co-dispensation periods shorter than the 14-day margin and attributed the episode to the OAT type encompassing the others. The *equals* relations with durations ≤14 days were excluded (*n*=15) (Fig. 5). Alternatively, if the co-dispensation period in Allen’s *overlaps*, *contains*, *starts*, *finishes*, or *equals* relation was >14 days, we classified the episode as an OAT multitherapy episode with no adjustments made to the relations (Fig. 6).

### OAT episodes and hospitalizations

In Appendix 7, we explored various scenarios to more precisely define an episode’s start and end dates when OAT is initiated following hospitalization, when hospitalization occurs before the preceding OAT episode reaches its discontinuation threshold, or when OAT regimen changes occur post-hospitalization.

We identified 10,386 episodes, of which 93.53% (*n*=9,714) were monotherapy, 3.72% (*n*=386) transition therapy, and 2.27% (*n*=236) multitherapy. Less than 1% (*n*=50) involved transition and multitherapy. The median (Q1, Q3) duration of all episodes was 39 days (13, 165).

## Discussion

We proposed a refined methodological framework to build OAT episodes in administrative health data by integrating Allen’s relation with the concepts of permissible gap and temporal margins ($\mathcal{E}$). This combined methodology improves upon using the permissible gap method alone. The permissible gap provides a framework for defining discontinuation windows, which are critical components in analyzing medication outcomes. However, this approach is limited by its reliance on a fixed temporal relation, accounting only for the *before* relation and failing to consider the magnitude of overlaps or other temporal relations. Therefore, extending the framework with Allen’s relations and temporal margins permits a more nuanced construction of OAT treatment episodes that better reflect the complexity of real-world OAT use.

Although in our study the predominant temporal relation between same-type OAT dispensations was *before*, we also observed a high proportion of *equals* relations for buprenorphine, SROM, and iOAT that may not have been adequately captured by the permissible gap method alone. The observed relations also align with clinical practice: gaps in the data reflected as *before* relations could be attributed to treatment interruptions, discontinuations, or delayed refills among patients with OUD. Conversely, *equals* relations are likely linked to same-day supplies for witnessed ingestions, as-needed OAT medications, or split dosing [4, 27].

Incorporating a temporal margin into Allen’s relations improves the handling of overlaps and reduces misclassification of OAT exposure by preventing artificially prolonged episodes. It also allows for differentiating episodes with transition therapy and multitherapy. Although Khotimah et al. previously applied temporal margins to relax Allen’s relations in a study of diabetes medications, their method applied $\mathcal{E}$ to both *overlaps* and *before* relations [19]. This approach treats any gap shorter than $\mathcal{E}$ as *meets* and continuous therapy, which makes the definition of a distinct discontinuation window dependent on the choice of $\mathcal{E}$. In contrast, our framework preserves the *before* relation through clinically informed permissible gaps [4], ensuring discontinuation windows remain grounded in OAT pharmacology and clinical practice.

Additionally, the temporal margin was applied in two ways in our analysis: (a) to construct monotherapy episodes, a seven-day margin was used to account for short versus long overlaps between same-type OAT dispensations, and (b) a 14-day margin was applied to differentiate transitions from multitherapy when more than one OAT was dispensed. This contrasts with the approach by Khotimah et al., in which the temporal margin was applied across diabetes medication classes to define continuous episodes, rather than distinguish transitions from multitherapy.

The selection of $\mathcal{E}$=7 days for same-OAT dispensations and $\mathcal{E}$=14 days for overlaps involving different OAT medications was informed by both empirical data and clinical practice. In constructing monotherapy episodes, we observed that recoding all *overlaps* to *meets* without a temporal margin ($\mathcal{E}$) led to substantial artificial shifts in Dates of Service, producing patterns that were inconsistent with real-world dispensing behaviour. Introducing a small margin ($\mathcal{E}$=7) effectively minimized these distortions while preserving clinically appropriate continuity of therapy. For different OAT dispensations, examination of co-dispensation lengths showed that most overlaps were less than 14 days. Input from an OAT-specialized clinical pharmacist and current treatment guidelines [4] supported the use of this range to represent intentional transitions. Longer overlap periods, by contrast, more often reflected concurrent therapy rather than transitional switching. Accordingly, a 14-day margin provides a clinically appropriate threshold for distinguishing transition episodes from multitherapy.

Additional support for these parameters comes from BC’s “refilling too soon” policy, which permits refills only when 14 or fewer days of supply remain [28]. This policy reflects routine dispensing patterns that naturally allow short overlaps of this magnitude. Longer overlaps, in contrast, are unlikely to represent routine continuation and should not be appended to the end of an episode, as they likely reflect a clinically meaningful change in treatment rather than ongoing therapy. Consistent with previous OAT research, treatment duration in our cohort was short, with the median episode lasting less than two months [6, 11, 29, 30]. In such settings, shorter temporal margins are more appropriate, as they better capture the brief periods of continuity, transition, or multitherapy that characterise real-world OAT use. Similar to our findings, Khotimah et al. also observed minimal changes in episode structure when margins exceeded 14 days [19].

As in Khotimah’s study, *starts*, *finishes*, and *contains* relations were relatively rare, but they remain important because they represent situations in which abrupt changes in a patient’s condition occur, requiring physician management. These changes might include instances when an OAT medication is taken as needed, vomited, lost, or used subtherapeutically.

In our analysis of PharmaNet data, the most frequent co-dispensation involved SROM and methadone (*n*=524) (Table 4), consistent with clinical practice for patients with more severe OUD [4]. Dispensation sequences further showed that individuals receiving methadone or SROM who were initiated on buprenorphine/naloxone often reverted to their original therapy (at least 26 observed sequences) (Table 4), which could reflect the higher retention observed with methadone [31] and likely suggesting a broader preference for full-agonist therapies.

The study has limitations inherent to administrative health databases. First, PharmaNet captures medications dispensed rather than actual ingestion, and it contains no information on patient intentions, provider-directed holds, or other behavioural factors that influence treatment continuity. Nevertheless, PharmaNet remains a uniquely valuable resource, providing population-wide, event-based dispensing records within a universal health system. Moreover, combining Allen’s relations with temporal margins and drug-specific permissible gaps provides a framework that bridges temporal logic with clinical guidelines, making it well-suited to event-based datasets such as PharmaNet. Alternative approaches—such as sequence-alignment and changepoint-detection algorithms—can characterize longitudinal treatment patterns but do not represent the temporal relationships between dispensations. Sequence-alignment methods treat medication data as a sequence of discrete states and are primarily intended to measure similarity between time-indexed observations for clustering or stratification [32]. Changepoint algorithms detect abrupt or significant shifts in regularly sampled time series [33]. Therefore, these approaches cannot account for clinically meaningful overlap lengths, washout periods, drug-specific discontinuation windows, or the ordering and relational structures captured by Allen’s relations, which are required to differentiate transition and multitherapy episodes. In contrast, our interval-based framework was purposefully developed for irregularly spaced dispensing data and enables clinically meaningful reconstruction of OAT use.

Second, our findings are most directly applicable to PLWH with OUD or OUD–StUD. However, our episode-construction framework is broadly transferable because Allen’s relations, temporal margins, and drug-specific permissible gaps operate on interval timing rather than on population- or jurisdiction-specific fields. In settings without explicit days supply information, intervals can be inferred from the quantity dispensed, standard dosing schedules, or local pharmacy practices. As such, the framework can be adapted for non-HIV populations and applied in international contexts with appropriate calibration of permissible gaps and temporal margins to reflect local clinical practice.

Third, our data ended in March 2020 and therefore do not capture the substantial changes in OAT delivery that occurred during the COVID-19 pandemic. We recommend that future studies apply this methodological framework to post-2020 data to assess how shifts in prescribing practices, increased use of take-home doses [9], and evolving models of care influence OAT episode structure, continuity, transitions, and multitherapy patterns.

Fourth, although external validation through chart review or comparison with a clinical gold standard would have strengthened the framework, it would have required additional administrative approvals, ethics amendments, and chart linkages beyond the scope and timelines of this methodological study. To enhance internal validity, an OAT-specialized clinical pharmacist (Christopher C. Fisher) reviewed all data-cleaning and episode-construction steps, and the conceptual framework was vetted by three physicians with addiction expertise (Drs. Ron Joe, Michael Mulvey, and Galilee Thompson). We also benchmarked our initial cleaning error rates against two prior studies led by our group—one among PLWH receiving OAT in BC [11] and another in a provincial OUD cohort [6]—both of which showed comparable patterns. Finally, the distribution of temporal relations in our cohort closely mirrored those reported by Khotimah et al. in a different therapeutic area, supporting the consistency of our interval-based approach [19].

## Conclusion

We developed a refined framework for constructing monotherapy, transition therapy, and multitherapy episodes in administrative health data by combining Allen’s relations with the permissible gap and data-driven temporal margins. Our methodology builds on previous research that either solely used the permissible gap to construct episodes for the same medication or used Allen’s relations with a temporal margin across different medication classes. By combining both techniques, we propose a more flexible and comprehensive approach that leverages the strengths of each, enabling more precise construction of medication episodes and better capture of the complexity of real-world patterns. By offering methodological tools to optimize the usability of administrative health data, this study contributes to clinical practice and policy development. It facilitates more precise assessments of OAT outcomes by properly considering overlaps, transitions, multitherapy, and temporal dynamics, which are increasingly relevant as OAT evolves to meet the complex needs of patients with OUD. Future studies could use this method to examine OUD populations and replicate its applicability using data from after 2020, a period marked by increased OAT transitions, combination therapies, and the COVID-19 pandemic.

## Supplementary Information

Below is the link to the electronic supplementary material.

Supplementary Material 1

Supplementary Material 2

Supplementary Material 3

## Data Availability

The sponsors had no role in the design, data collection, analysis, interpretation, or report writing. All inferences, opinions, and conclusions drawn in this publication are those of the authors and do not reflect the opinions or policies of the Data Stewards. The BC-CFE is prohibited from making individual-level data available publicly due to provisions in our service contracts, institutional policy, and ethical requirements. We make such data available via individual data access requests to facilitate research. Some BC-CFE data is unavailable externally due to prohibitions in service contracts with our funders or data providers. Institutional policies stipulate that all external data requests require collaboration with a BC-CFE researcher. For more information or to make a request, please contact privacy@bccfe.ca. All data relevant to the analysis are included in the tables or supplementary materials. The underlying analytical codes are attached as an Appendix.
