# Supplementary material for: A methodological framework for constructing opioid agonist therapy episodes in administrative health data
Source: BMC Methods. 2026 Mar 12;3(1):11. doi: 10.1186/s44330-026-00064-9 (PMC12979340; doi:10.1186/s44330-026-00064-9)
Supplement: Supplementary file 2 — Supplementary Material 2 [file 44330_2026_64_MOESM2_ESM.docx]

Appendix Table of Contents

[**Appendix 1. Study Sample** 2](#_Toc215661364)

[**Appendix 2. Identifying OAT in Administrative Databases** 4](#_Toc215661365)

[**Appendix 2 Table.** Feeitems in the MSP database to identify cases of OUD or individuals receiving OAT 5](#_Toc215661366)

[**Appendix 3 Figure.** The cleaning procedure implemented by Pearce and colleagues (2020) 6](#_Toc215661367)

[**Appendix 3 Table a**. Summary of Logic-Based Cleaning Procedures Applied to PharmaNet OAT Dispensations Before Episode Construction. 7](#_Toc215661368)

[**Appendix 3 Table b.** Comparison of the initial PharmaNet data cleaning process in the current study with that of previously published studies 8](#_Toc215661369)

[**Appendix 4. Examples of OAT Data Discrepancies in PharmaNet** 9](#_Toc215661370)

[**Appendix 5. Allen’s Relations in Single-OAT Episodes: Temporal Margin Analysis** 11](#_Toc215661371)

[**Appendix 5 Table a.** Temporal margin analysis with no Ɛ value 11](#_Toc215661372)

[**Appendix 5 Table b.** Temporal margin analysis with Ɛ = 7 days 11](#_Toc215661373)

[**Appendix 5 Table c.** Temporal margin analysis with Ɛ = 14 days 11](#_Toc215661374)

[**Appendix 6. Additional Multiple OATs Sequence of Dispensations** 12](#_Toc215661375)

[**Appendix 6 Table.** Multiple OAT dispensation sequences with **frequency <5** were identified in our PharmaNet data exploration 12](#_Toc215661376)

[**Appendix 7. Hospitalizations** 14](#_Toc215661377)

[**Scenario 1:** Hospitalization occurs within the grace period of OAT1, and there are no more OAT dispensations 14](#_Toc215661378)

[**Scenario 2:** Hospitalization occurs within the grace period of OAT1, and there are OAT dispensations following discharge 14](#_Toc215661379)

[**Scenario 3:** OAT dispensations follow a discharge (The therapeutic dose should be recorded on the dispensing date rather than the dose reached later during treatment) 14](#_Toc215661380)

[**Scenario 4:** Hospitalization is nested within an OAT episode 15](#_Toc215661381)

[**Scenario 5:** Hospitalization overlaps with OAT episode (we did not shift the hospitalization) 15](#_Toc215661382)

[**Scenario 6:** Hospitalization in a transition therapy episode 15](#_Toc215661383)

[**Scenario 7:** Hospitalization in a multitherapy episode 16](#_Toc215661384)

# **Appendix 1. Study Sample**

**Data Source:** The data were collected from the Seek and Treat for Optimal Prevention of HIV/AIDS (STOP HIV/AIDS) study, an open, bidirectional, population-based longitudinal cohort housed at the BC Centre for Excellence in HIV/AIDS (BC-CfE). This study contains de-identified, individual-level data for adults aged 19 and older living with HIV, covering the period from April 1, 1996, to March 31, 2020. People living with HIV (PLWH) were ascertained via having a detectable plasma viral load for HIV, a dispensed antiretroviral drug for HIV treatment, a positive laboratory result indicating HIV infection (reported to the BCCDC database), an HIV/AIDS-related death, and/or meeting the criteria of a high-specificity healthcare contact-based case-finding algorithm.^1^

We identified cases of opioid use disorder (OUD) alone and those with concurrent OUD and stimulant use disorder (StUD) using case-finding algorithms published by Palis et al.^2, 3^, Keen et al.,^4^ and Janjua et al.,^5^ with the International Classification of Diseases codes from the 9th revision, 9th revision clinical modification, and 10th Canadian revision (ICD-9/9-CM/10-CA). A 5-year wash-out period was applied to ensure incident cases were captured.^6^ Two administrative databases from the STOP HIV/AIDS cohort were used:

- **Medical Services Plan Payment Information File (MSP):** This database includes all health practitioner claims, each associated with a single ICD-9/9-CM diagnostic code indicating the reason for the visit ^7^
- **Discharge Abstract Database (DAD):** This database includes acute inpatient hospitalizations in British Columbia hospitals, including up to 25 diagnostic codes (ICD-10-CA, starting April 2001) per hospitalization, with one code identifying the single reason/issue most responsible for a person’s overall hospital stay and 20 procedure codes^8^

**OUD Assessment:** All cases of OUD were identified as having one hospitalization record in DAD [any position] or two health practitioner encounters in MSP within 12 months

- ICD-9/9-CM: 3040, 3047, 3055, E8500, 9650
- ICD-10-CA: F11, T400, T401, T402, T403, T404, R781, T507, Y4509, Y501
- **Exclusion of StUD Cases:** Among identified cases of OUD, we excluded individuals with any indication of StUD using the below codes in either MSP or DAD
  - ICD-9/9-CM: 3042, 3044, 3056, 3057, 9697, 970, 9700, 9701, 9708, 9709, 76075, E8542, E8543, E9397, E9408, E9409
  - ICD-10-CA: F14, F15, T405, R782, T436, Y497, Y508, Y509, T460, T472, Y520, Y532

**StUD Assessment:** Cases of StUD were identified as having one hospitalization record in DAD [any position] or two health practitioner visits in MSP within 12 months

- ICD-9/9-CM: 3042, 3044, 3056, 3057, 9697, 970, 9700, 9701, 9708, 9709, 76075, E8542, E8543, E9397, E9408, E9409
- ICD-10-CA: F14, F15, T405, R782, T436, Y497, Y508, Y509, T460, T472, Y520, Y532

**OUD-StUD Assessment:** Persons who meet both definition criteria for OUD and StUD

**Note:** The study samples (PLWH-OUD) and (PLWH-OUD-StUD) were initially developed to assess the impact of StUD on OAT outcomes. The methodological framework for OAT was subsequently developed as part of a secondary analysis for this project.

# **Appendix 2. Identifying OAT in Administrative Databases**

**OAT Availability and Regulations**

The OAT program in BC was established in 1996 and has undergone significant development and expansion since then. Before 2008, oral solution methadone was the main pharmacotherapy for OUD prescribed by licensed physicians. In 2008, Buprenorphine/Naloxone was added to the provincial drug formulary and recommended as the first-line treatment in 2017 due to its safety profile, lower risk of fatal overdose, and faster induction schedule. The slow-release oral morphine (SROM) was approved for use in Canada in November 2014 and recommended as an option for patients who have not responded to previous treatments. Injectable OAT (iOAT) with diacetylmorphine (pharmaceutical-grade heroin) was available in research settings before 2016. It was formally approved in BC under supervised conditions in May 2016, and the government of Canada officially approved hydromorphone in May 2019 as a second iOAT medication to treat severe OUD. In 2023, OAT guidelines were revised to emphasize tailoring treatment based on patient-specific factors, preferences, and goals. The clinical practice also reflects the increasing use of combined OAT regimens, especially pairing iOAT with SROM and methadone, as a response to escalating opioid tolerance and severe OUD cases.^9^

**Identifying OAT Using MSP**

Researchers may additionally use MSP to identify people receiving OAT via MSP feeitems (i.e., a unique billing code linked to a specific medical service within Canada’s healthcare system). These fee items suggest a wider application of point-of-care (POC) urine drug testing (UDT) to manage OUD or other forms of substance use.^9^ It is possible that physicians, when seeing a new patient with OUD for the first time, code MSP 39. However, there is a chance the patient will not follow through and pick up the prescribed medication, and therefore may not appear in PharmaNet. Since MSP 39 is billed weekly per patient, a patient might see a physician and receive their prescribed OAT at the pharmacy on the same day, while the physician might not submit the MSP billing until later in the week, such as over the weekend. Also, before 2020, there were only MSP fee codes 39 and 15039, and other MSP fee codes were introduced after 2020. MSP 15039 is more for controlling and monitoring, with urine drug testing allowed only 26 times per year, whereas MSP 39 is permitted around 50 times annually.

## **Appendix 2 Table.** Feeitems in the MSP database to identify cases of OUD or individuals receiving OAT

| Management of OAT maintenance | 39 |
| --- | --- |
| General practitioner POC testing for OAT | 15039 |
| Assessment for induction of OAT | 13013 |
| Management of OAT induction for OUD | 13014 |
| Nurse practitioner POC testing for OAT | 36521 |
| General practitioner POC testing for amphetamines, opioids, and oxycodone | 15040 |
| Nurse practitioner POC testing for amphetamines, benzodiazepines, buprenorphine/naloxone, cocaine metabolites, methadone metabolites, opioids, and oxycodone | 36522 |

Table Abbreviations**:** OAT: Opioid Agonist Therapy; POC: Point of Care; OUD: Opioid Use Disorder

In a previously published work, we found that 1,515 (13.8%) of PLWH (n=10,959) in the STOP cohort between 2008 and 2020 had at least one OAT dispensation recorded in the PharmaNet database. Using the MSP fee item 39, we identified an additional 197 people, 152 of whom had no PharmaNet records. The remaining 45 had PharmaNet records, but none for any of the OAT DIN/PINs we used. All individuals with fee item 15039 also had fee item 39, and none had fee items 13013 or 13014.^10^

**Appendix 3. Initial PharmaNet Cleaning Process**

Careful cleaning processes were implemented to identify and correct potential errors and ensure error-free, accurate OAT records in the PharmaNet data. The primary cleaning process followed the approach outlined by Pearce and colleagues (Figure a).^11^

##
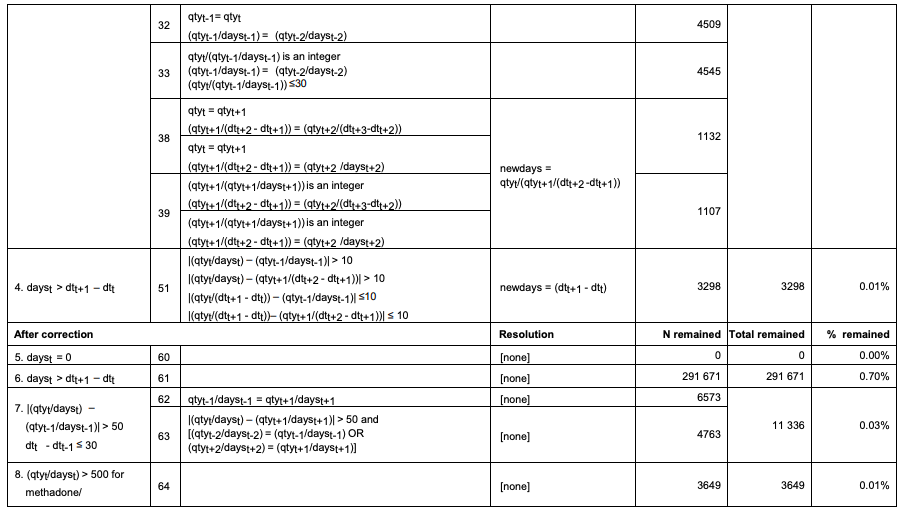

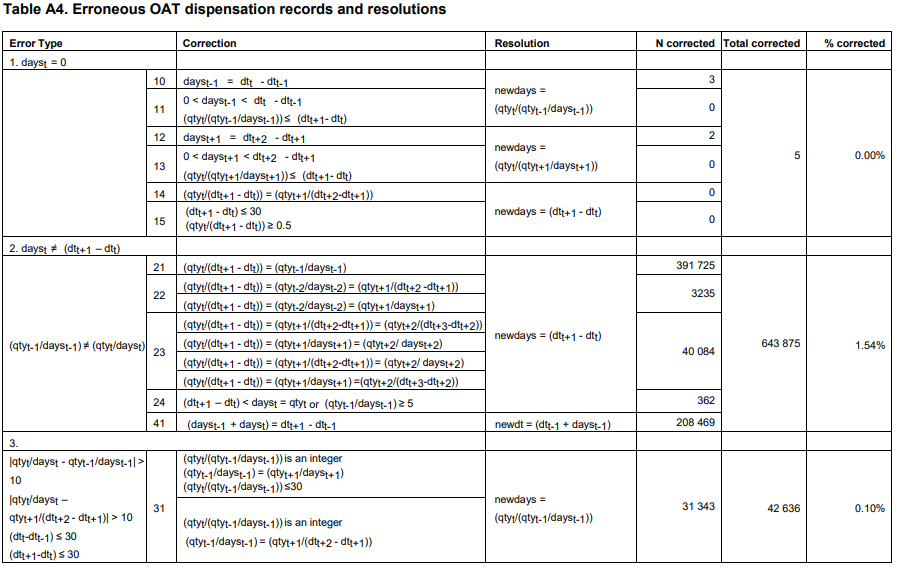
**Appendix 3 Figure.** The cleaning procedure implemented by Pearce and colleagues (2020)

## **Appendix 3 Table a**. Summary of Logic-Based Cleaning Procedures Applied to PharmaNet OAT Dispensations Before Episode Construction.

| Cleaning Step | Issue Addressed | Logic / Rule Applied | Correction Applied | Notes |
| --- | --- | --- | --- | --- |
| 1. Split-dose consolidation | Multiple same-day dispensations of the same drug type, same-day supply. | Group by drug type, days supply, and DoS. | Sum quantities into a single record. | Grouping by days supply in the case of a split dose and early-refill on the same day. |
| 2. Error 2:  Days supply and QPD mismatch | Days supply inconsistent with dispensing interval; QPD mismatch. | Rules 21 | Recalculate days supply to match interval OR shift service date (rule 41). | None |
| 3. Error 3:  Large QPD anomalies | QPD differs from previous/next by >10. | Rules 31–33, 38–39 | Recalculate the days supply using the previous or next QPD. | New days supply constraint ≤30 days.  Did not update if dose/day is consistent with previous and next dose/day. |
| 4. Error 4:  Days supply > period | Days supply exceeds observed interval. | Rule 51 | Reset the days supply to the period length. | None |
| 5. Error 5 :  Outlier quantity/day patterns | quantity or days supply abnormal | Fix 5a & 5b | Recalculate days supply (5a) or previous QPD (5b). | Only change the quantity if an inconsistent dose, not a split-dose dispensation. |

Note: Examples are provided in the supplementary Excel file.

Table Abbreviations: DoS: Date of Service; QDP: Quantity per Day.

## **Appendix 3 Table b.** Comparison of the initial PharmaNet data cleaning process in the current study with that of previously published studies

|  | **Current Study^֎^** | **Yazdani, et al.^֎֎^** | **Bohdan, et al.^֎֎֎^** |
| --- | --- | --- | --- |
| **Total OAT Dispensations** | 2,094,532 | 3,854,055 | 41,704,201 |
| **Errors Identified** | 21,977 (1.05%) | 114,290 (2.9%) | 996,470 (2.3%) |
| **Errors Corrected** | 8,993 (40.92%) | 27,236 (23.8%) | 689,814 (69.2%) |

**^֎^** PLWH diagnosed with OUD or OUD=StUD dispensed with at least one OAT)

**^֎֎^** PLWH dispensed with at least one OAT^10^

**^֎֎֎^** People with OUD^11^

Table Abbreviations: OAT: Opioid Agonist Therapy; PWH: People with HIV; OUD: Opioid Use Disorder; StUD: Stimulant Use Disorder

# **Appendix 4. Examples of OAT Data Discrepancies in PharmaNet**

Examples of data discrepancies in OAT dispensations within the PharmaNet data and potential explanations provided by our co-author, pharmacist Chase Fisher

**
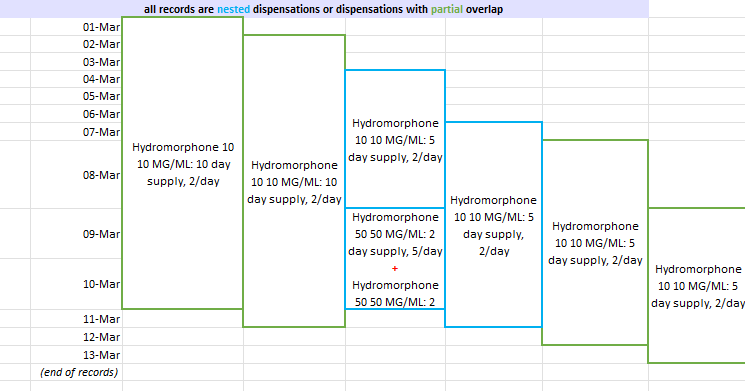
Example 1.** On March 1, a 10-day HDM 10 mg/mL supply was dispensed. The client likely required a higher dose, so a subsequent Rx was written to top up doses on March 2 (10-day supply).

**
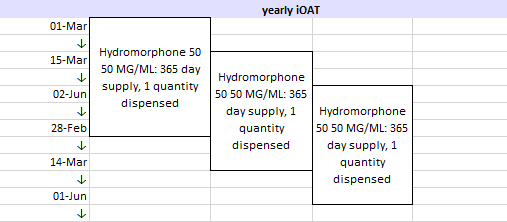
Example 2.** We had 41 yearly iOAT dispensations that were considered potential entry errors and were excluded.

**
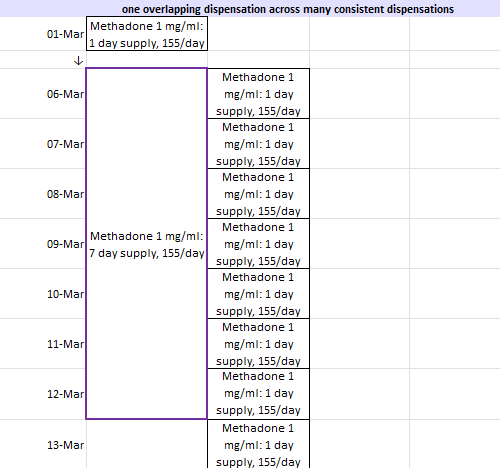
Example 3.** Methadone in the community is typically 10 mg/mL commercial strength. Methadol 1 mg/mL is usually provided in hospitals, or it could be compounded methadone 1 mg/mL. It seems this is workflow-related—the pharmacy dispenses multi-day supplies to deliver to the client’s facility (a typical PharmaNet pattern in recently admitted/incarcerated people). Nested dispensations could suggest they were not admitted or needed a new replacement, or the dose was stolen, vomited, or subtherapeutic.

**
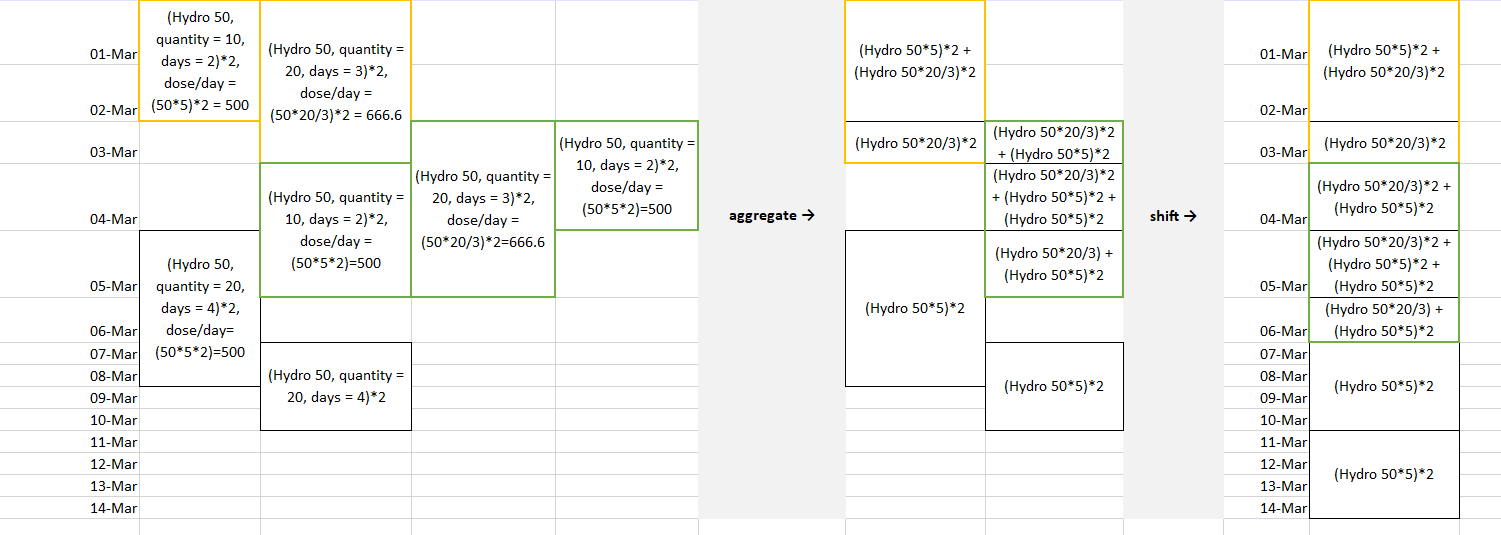
Example 4.** An example of applying Allen’s relations and data manipulation techniques to PharmaNet dispensations for constructing continuous single-OAT episodes. In this figure, before aggregation and shifting, we can observe the following Allen’s relations: starts (yellow dispensations), “*starts*” and “*finishes*” (green dispensations), and “*overlaps*” (green and black dispensations).

# **Appendix 5. Allen’s Relations in Single-OAT Episodes: Temporal Margin Analysis**

## **Appendix 5 Table a.** Temporal margin analysis with no Ɛ value

| **Difference Between Date of Service and New Shifted Date of Service (Days)**  **No Ɛ** **Applied (Adding All Overlapping Supply)** | | | | | | | |
| --- | --- | --- | --- | --- | --- | --- | --- |
|  | **N** | **NMiss** | **Mean (SD)** | **Median** | **Mode** | **Min** | **Max** |
| **Methadone** | 1,784,881 | 0 | 0.23 (2.97) | 0 | 0 | 0 | 233 |
| **Buprenorphine** | 50,134 | 0 | 0.66 (4.45) | 0 | 0 | 0 | 87 |
| **SROM** | 164,899 | 0 | 5.44 (28.03) | 0 | 0 | 0 | 695 |
| **iOAT** | 33,957 | 0 | 0.18 (3.02) | 0 | 0 | 0 | 105 |

## **Appendix 5 Table b.** Temporal margin analysis with Ɛ = 7 days

| **Difference Between Date of Service and New Shifted Date of Service (Days)**  **Ɛ=7 Days** | | | | | | | |
| --- | --- | --- | --- | --- | --- | --- | --- |
|  | **N** | **NMiss** | **Mean (SD)** | **Median** | **Mode** | **Min** | **Max** |
| **Methadone** | 1,784,881 | 0 | 0 (0.09) | 0 | 0 | 0 | 15 |
| **Buprenorphine** | 50,134 | 0 | 0.03 (0.39) | 0 | 0 | 0 | 19 |
| **SROM** | 164,899 | 0 | 0.12 (1.71) | 0 | 0 | 0 | 98 |
| **iOAT** | 33,957 | 0 | 0.02 (0.56) | 0 | 0 | 0 | 28 |

## **Appendix 5 Table c.** Temporal margin analysis with Ɛ = 14 days

| **Difference Between Date of Service and New Shifted Date of Service (Days)**  **Ɛ=14 Days** | | | | | | | |
| --- | --- | --- | --- | --- | --- | --- | --- |
|  | **N** | **NMiss** | **Mean (SD)** | **Median** | **Mode** | **Min** | **Max** |
| **Methadone** | 1,784,881 | 0 | 0.01 (0.57) | 0 | 0 | 0 | 232 |
| **Buprenorphine** | 50,134 | 0 | 0.04 (0.51) | 0 | 0 | 0 | 28 |
| **SROM** | 164,899 | 0 | 0.23 (3.91) | 0 | 0 | 0 | 260 |
| **iOAT** | 33,957 | 0 | 0.03 (0.57) | 0 | 0 | 0 | 28 |

# **Appendix 6. Additional Multiple OATs Sequence of Dispensations**

## **Appendix 6 Table.** Multiple OAT dispensation sequences with **frequency <5** were identified in our PharmaNet data exploration

| (SROM + Methadone)🡪 (iOAT + SROM + Methadone)🡪(SROM + Methadone) |
| --- |
| (SROM + Methadone)🡪(iOAT + SROM + Methadone)🡪(iOAT + SROM) |
| (SROM + Methadone)🡪(iOAT + SROM + Methadone)🡪Methadone |
| (SROM + Methadone)🡪(iOAT + SROM)🡪(SROM + Methadone) |
| (SROM + Methadone)🡪(iOAT + SROM)🡪iOAT |
| (iOAT + Methadone)🡪(iOAT + SROM + Methadone)🡪(SROM + Methadone) |
| (iOAT + Methadone)🡪 (iOAT + SROM + Methadone)🡪(iOAT + Methadone) |
| (iOAT + Methadone)🡪(iOAT + SROM + Methadone)🡪(iOAT + SROM) |
| (iOAT + Methadone)🡪(iOAT + SROM)🡪iOAT |
| (iOAT + SROM + Methadone)🡪(SROM + Methadone)🡪Methadone |
| (iOAT + SROM + Methadone)🡪(iOAT + Methadone)🡪(iOAT + SROM + Methadone) |
| (iOAT + SROM + Methadone)🡪(iOAT + Methadone)🡪Methadone |
| (iOAT + SROM + Methadone)🡪(iOAT + SROM)🡪SROM |
| (iOAT + SROM + Methadone)🡪(iOAT + SROM)🡪iOAT |
| (iOAT + SROM)🡪(Buprenorphine + iOAT + SROM)🡪Buprenorphine |
| (iOAT + SROM)🡪(SROM + Methadone)🡪Methadone |
| (iOAT + SROM)🡪(iOAT + Methadone)🡪Methadone |
| (iOAT + SROM)🡪(iOAT + Methadone)🡪iOAT |
| (iOAT + SROM)🡪(iOAT + SROM + Methadone)🡪(iOAT + Methadone) |
| (iOAT + SROM) 🡪(iOAT + SROM + Methadone) 🡪 iOAT |
| Buprenorphine🡪(SROM + Methadone)🡪Methadone |
| Methadone🡪(Buprenorphine + SROM + Methadone)🡪Methadone |
| Methadone🡪(SROM + Methadone)🡪(iOAT + SROM + Methadone) |
| Methadone 🡪 (SROM + Methadone) 🡪 (iOAT + SROM) |
| Methadone🡪(iOAT + Methadone)🡪(iOAT + SROM) |
| Methadone🡪(iOAT + SROM)🡪(iOAT + SROM + Methadone) |
| Methadone🡪(iOAT + SROM)🡪Methadone |
| Methadone🡪(iOAT + SROM)🡪iOAT |
| SROM🡪(iOAT + SROM)🡪(Buprenorphine + iOAT + SROM) |
| SROM🡪(iOAT + SROM + Methadone)🡪SROM |
| iOAT🡪(iOAT + Methadone)🡪(iOAT + SROM + Methadone) |
| iOAT🡪(iOAT + Methadone)🡪(iOAT + SROM) |
| iOAT🡪(iOAT + SROM + Methadone)🡪(iOAT + SROM) |
| iOAT🡪(iOAT + SROM)🡪(iOAT + Methadone) |
| iOAT 🡪 (iOAT + SROM) 🡪 (iOAT + SROM + Methadone) |

# **Appendix 7. Hospitalizations**

Hospitalization can present both advantages and significant risks for individuals with OUD. Risks include limited harm reduction supplies and safe spaces for substance use. Advantages include opportunities to optimize OAT treatment, address co-occurring substance use or other comorbidities, and assess the need for more intensive support systems.^12^ Here, with consultation with an addiction psychiatrist, Dr. Ron Joe, we defined seven scenarios to determine the start and end dates of OAT episodes more accurately. These scenarios address cases where OAT begins after hospitalization, hospitalization occurs before the previous OAT episode reaches its discontinuation threshold, or changes in the OAT regimen occur following hospitalization.

## **Scenario 1:** Hospitalization occurs within the grace period of OAT1, and there are no more OAT dispensations


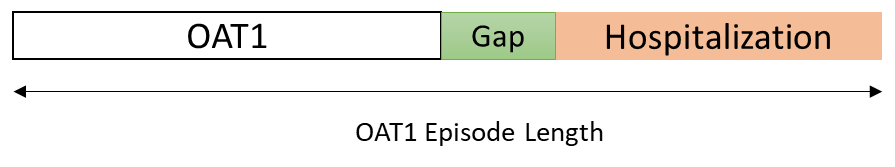


## **Scenario 2:** Hospitalization occurs within the grace period of OAT1, and there are OAT dispensations following discharge


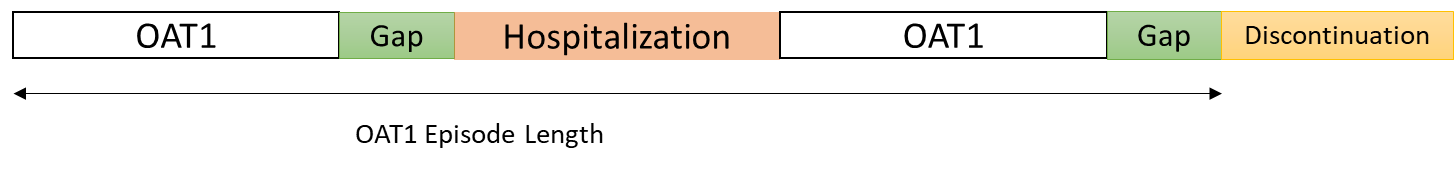


##
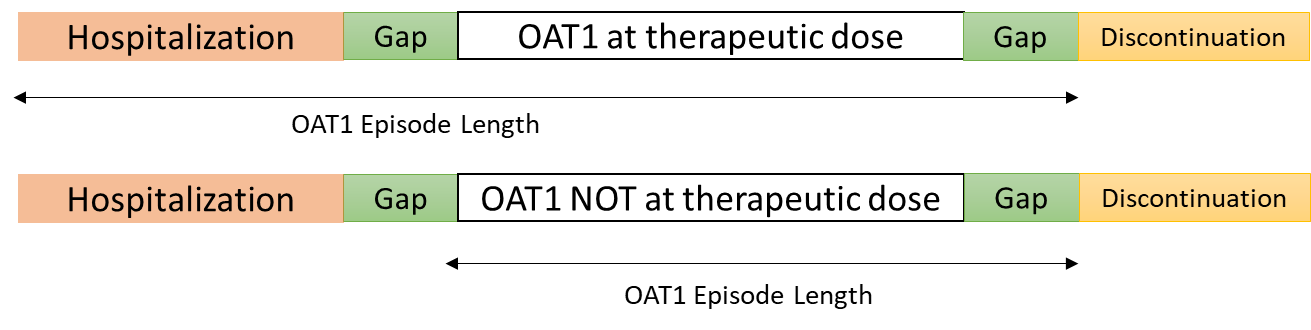
**Scenario 3:** OAT dispensations follow a discharge (The therapeutic dose should be recorded on the dispensing date rather than the dose reached later during treatment)

## **Scenario 4:** Hospitalization is nested within an OAT episode


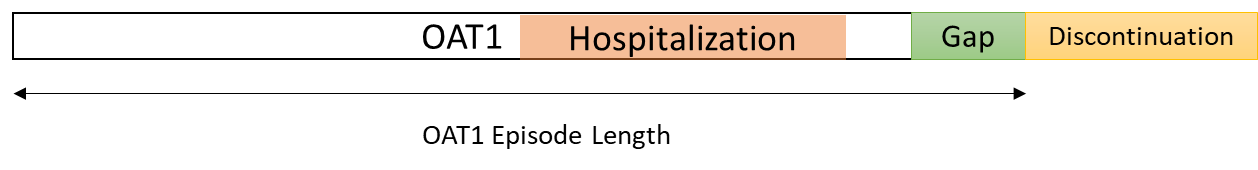


## **Scenario 5:** Hospitalization overlaps with OAT episode (we did not shift the hospitalization)


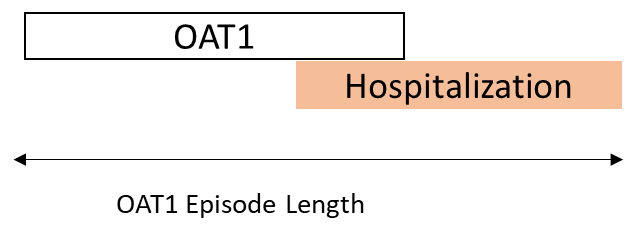


## **Scenario 6:** Hospitalization in a transition therapy episode


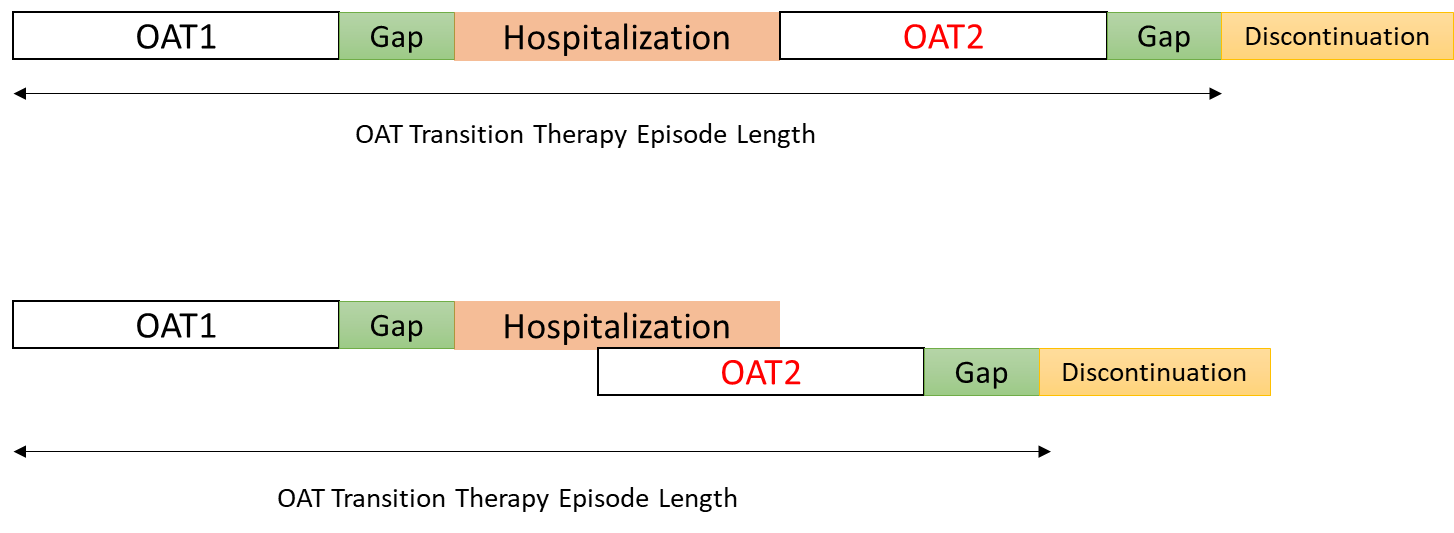


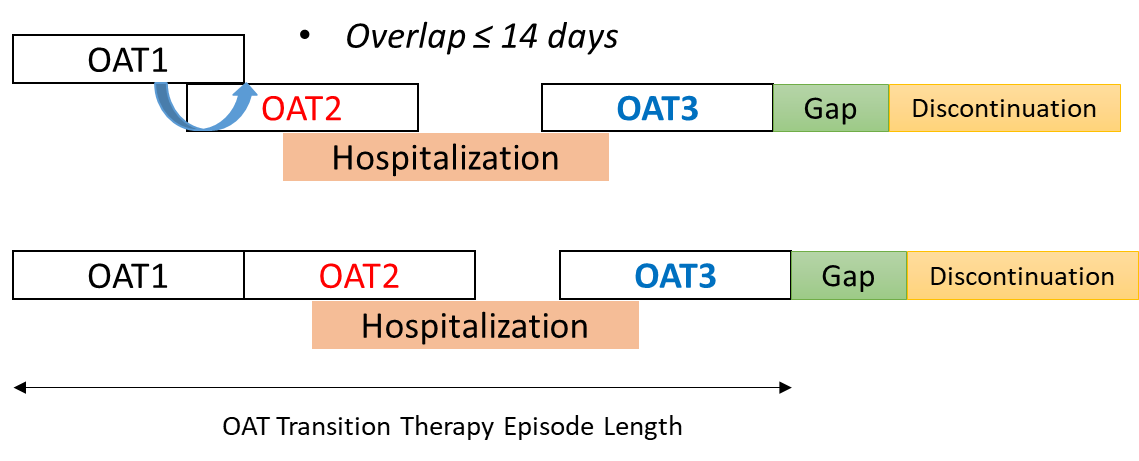


## **Scenario 7:** Hospitalization in a multitherapy episode

**
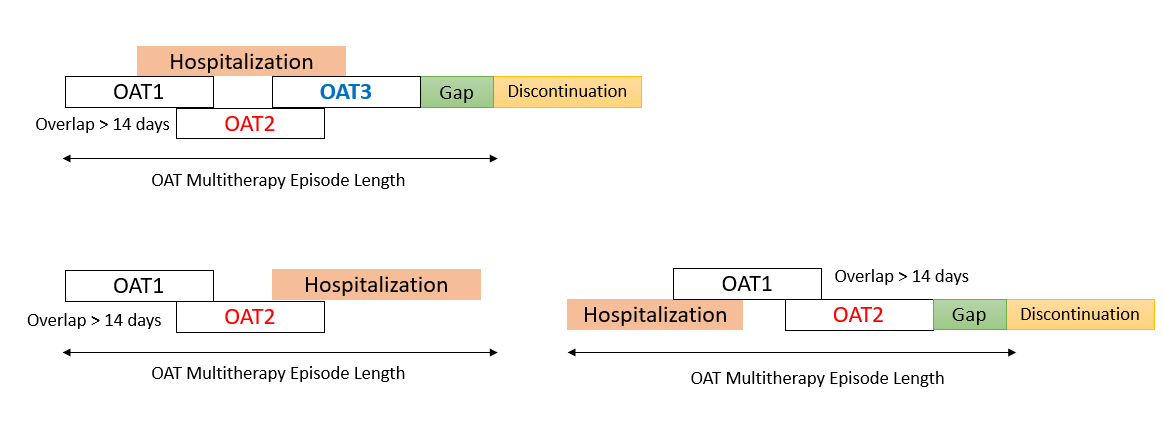
**

**Appendix References**

**1.** Emerson SD, McLinden T, Sereda P, et al. Identification of people with low prevalence diseases in administrative healthcare records: A case study of HIV in British Columbia, Canada. *PLoS One.* 2023;18(8):e0290777.

**2.** Palis H, Xavier C, Dobrer S, et al. Concurrent use of opioids and stimulants and risk of fatal overdose: A cohort study. *BMC Public Health.* 2022/11/15 2022;22(1):2084.

**3.** Palis H, Zhao B, Young P, et al. Stimulant use disorder diagnosis and opioid agonist treatment dispensation following release from prison: a cohort study. *Subst Abuse Treat Prev Policy.* Nov 24 2022;17(1):77.

**4.** Keen C, Kinner SA, Young JT, et al. Prevalence of co-occurring mental illness and substance use disorder and association with overdose: a linked data cohort study among residents of British Columbia, Canada. *Addiction.* Jan 2022;117(1):129-140.

**5.** Janjua NZ, Islam N, Kuo M, et al. Identifying injection drug use and estimating population size of people who inject drugs using healthcare administrative datasets. *Int J Drug Policy.* May 2018;55:31-39.

**6.** Nanditha NGA, Dong X, McLinden T, et al. The impact of lookback windows on the prevalence and incidence of chronic diseases among people living with HIV: an exploration in administrative health data in Canada. *BMC Med Res Methodol.* Jan 6 2022;22(1):1.

**7.** BC Ministry of Health [creator] (2020): Medical Services Plan (MSP) Payment Information File. BC Ministry of Health [publisher]. Data Extract. MOH (2020). Available at: <https://www2.gov.bc.ca/gov/content/health/conducting-health-research-evaluation/data-access-health-data-central>.

**8.** Canadian Institute for Health Information. Discharge Abstract Database (Hospital Separations).V2. Available at: <https://www.popdata.bc.ca/data/health/dad>.

**9.** British Columbia Centre on Substance Use. A Guideline for the Clinical Management of Opioid Use Disorder. 2023.

**10.** Yazdani K, Dolguikh K, Ye M, et al. Characterizing opioid agonist therapy uptake and factors associated with treatment retention among people with HIV in British Columbia, Canada. *Prev Med Rep.* Oct 2023;35:102305.

**11.** Pearce LA, Min JE, Piske M, et al. Opioid agonist treatment and risk of mortality during opioid overdose public health emergency: population based retrospective cohort study. *Bmj.* Mar 31 2020;368:m772.

**12.** Centre for Addition and Mental Health. Opioid Agonist Therapy: A Synthesis of Canadian Guidelines for Treating Opioid Use Disorder. Available at: <https://www.camh.ca/-/media/professionals-files/canadian-opioid-use-disorder-guideline2021-pdf.pdf>.
