# Supplementary material for: A methodological framework for constructing opioid agonist therapy episodes in administrative health data
Source: BMC Methods. 2026 Mar 12;3(1):11. doi: 10.1186/s44330-026-00064-9 (PMC12979340; doi:10.1186/s44330-026-00064-9)
Supplement: Supplementary file 3 — Supplementary Material 3 [file 44330_2026_64_MOESM3_ESM.docx]

/* A Methodological Framework for Constructing Opioid Agonist Therapy Episodes in Administrative Data: Insights and Key Challenges

* Researcher: Kiana Yazdani

* Analyst: Cassidy Tam

* Related to request 434: Impact of stimulant use disorder on outcomes related to opioid agonist therapy in people living with HIV

* June/03/2025 */

/* PharmaNet OAT cleaning following the methods described in:

* Opioid agonist treatment and risk of mortality during opioid overdose public health emergency:

population based retrospective cohort study by Lindsay Pearce et al. and

* Characterizing opioid agonist therapy uptake and factors associated with treatment retention among

people with HIV in British Columbia, Canada by Yazadani et al.

* This code builds on the work done by Katerina Dolguikh (request 351-01)

* GOAL: reduce human error

* DEFINITIONS:

- QUANTITY RATIO: (quantity dispensed / days supply)

- PERIOD: date of following prescription - date of prescription

* PHARMANET VARIABLES:

- days_supply = estimated/intended number of days product should last for

- quantity_dispensed = total amount of product dispensed

- date_of_service = drug dispensation date

- moh_id = unique participant ID

* Base each fix on previous fixes (i.e. use updated data set based on fix 2 for fix 3 etc.)

* Need to reset previous and next values before each fix

* Note that taking the previous and 2nd previous value / next and 2nd next value does not account for split doses

- Split doses will have the same quantity dispensed, days supply, and date of service

- In the case that a record is preceeded or followed by a split dose then the (T-1)=(T-2) and (T+1)=(T+2)

- Most fixes do not compare (T-1) and (T-2) or (T+1) and (T+2) values

- In the fixes that do (fix 2: a3, fix 3), most of the values align anyways for this study period (>= 2010)

* In some cases, a dispensation is fixed to have same days supply as other dispensations on the same day, creating a new split dose

- Note that this fix does not necessarily mean the new split dose is consistent with previous/next dispensations

- Future work could consider applying a fix to non-split dose dispensations

- Future work could consider redefining split doses after each fix, but these new split doses may be more of a coincidence

* To create split doses, group by participant ID (moh_id), date of service, and days supply

- Ordering by days supply, in the case of non-split doses can change the order of dispensations

* Future work could also consider the typical days supply of specific OAT drugs as they may differ between drugs

and could incorrectly be classified as an error

- For example Sublocade is typically prescribed once per month, so days supply should also be around 1 month,

which will differ from the typical days supply of other Buprenorphine drugs */

**/* Split-dose consolidation */**

**/* Split-dose consolidation */**

**/* Split-dose consolidation */**

**/* Split-dose consolidation */**

/* Code to identify split doses before cleaning

* split dose = OAT products and supply days dispensed on the same day are similar

* need to consider case of > 2 dispensations on the same day - sort by days supply

* count number of rows for each unique (moh_id, date of service, days supply) combination */

**%macro** getSplitDose(drug_grp, in_data);

**%macro** ***getColours***;

**%mend** getColours;

**proc** **sql**;

create table &drug_grp._multi_tmp as

select distinct moh_id, date_of_service, days_supply, count(*) as num_rows

from &in_data.

group by moh_id, date_of_service, days_supply;

**quit**;

/* if >1 row for a given (moh_id, date of service, days supply) combo then considered a split dose */

**data** &drug_grp._multi_tmp2;

set &drug_grp._multi_tmp;

by moh_id date_of_service;

if num_rows > **1** then

split_flag = **1**;

/* not a split dose but multiple dispensation on same day */

else if not (first.date_of_service and last.date_of_service) then

split_flag = **2**;

else split_flag = **0**;

**run**;

/* add back split_flag */

**proc** **sql**;

create table &drug_grp._multi_tmp3 as

select a.*, b.split_flag

from &in_data. a

left join &drug_grp._multi_tmp2 b on a.moh_id = b.moh_id and a.date_of_service = b.date_of_service and a.days_supply = b.days_supply

order by a.moh_id, a.date_of_service, a.days_supply;

**quit**;

/* sum up quantity dispensed and oat dose for split doses

* last record will have corresponding quantity dispensed and oat dose for each unique (moh_id, date_of_service, days_supply) combo */

**data** &drug_grp._multi_tmp4;

set &drug_grp._multi_tmp3;

by moh_id date_of_service days_supply;

retain quant_disp_sum oat_dose_sum;

if first.moh_id or first.date_of_service or first.days_supply then

do;

quant_disp_sum = quantity_dispensed;

oat_dose_sum = quantity_dispensed*oat_drug_quant;

end;

else if split_flag = **1** then

do;

quant_disp_sum = quant_disp_sum + quantity_dispensed;

oat_dose_sum = oat_dose_sum + quantity_dispensed*oat_drug_quant;

end;

if last.days_supply;

**run**;

/* add back summed up quantity dispensed and oat dose */

**proc** **sql**;

create table &drug_grp._multi_out as

select a.*, b.quant_disp_sum as quan_use, b.oat_dose_sum, b.split_flag

from &in_data. a

left join &drug_grp._multi_tmp4 b on a.moh_id = b.moh_id and a.date_of_service = b.date_of_service and a.days_supply = b.days_supply

order by a.moh_id, a.date_of_service, a.days_supply;

**quit**;

/* flag non-first records within split dose group */

**data** &drug_grp._multi_out;

set &drug_grp._multi_out;

by moh_id date_of_service days_supply;

if not first.days_supply then

split_flag2 = **1**;

else split_flag2 = **0**;

**run**;

/* Delete temporary data sets to save work space */

**proc** **datasets** lib=work nolist;

delete &drug_grp._multi_tmp:;

**run**;

**%mend** getSplitDose;

/* Code to get previous and next values

* Add variables used in cleaning

* add prev and next days supply, quantity, and date

* as well as quantity ratio */

**%macro** getPrevNext(data_in, data_out);

**%macro** ***getColours***;

**%mend** getColours;

**proc** **sort** data=&data_in. out=prev_tmp;

by moh_id date_of_service days_supply;

**run**;

**data** prev_tmp1;

set prev_tmp;

by moh_id date_of_service;

/* previous variables */

prev_days = lag(days_supply);

prev_quan = lag(quan_use);

prev_rx_date = lag(date_of_service);

prev_drug = lag(oat_drug_name);

/* use OAT drug dose SUM to handle split doses

* if not a split dose then OAT drug dose SUM = quantity_dispensed*oat_drug_quant */

prev_oat_quant = lag(oat_dose_sum);

if first.moh_id then

do;

prev_days = **.**;

prev_quan = **.**;

prev_rx_date = **.**;

prev_drug = '';

prev_oat_quant = **.**;

end;

prev_period = date_of_service - prev_rx_date;

prev_quan_pday = prev_quan / prev_days;

/* for some parts of the cleaning process, need quantity at time T-2 */

prev2_quan_pday = lag(prev_quan_pday);

prev2_quan = lag(prev_quan);

if first.moh_id then

do;

prev2_quan = **.**;

prev2_quan_pday = **.**;

end;

/* quantity per day */

quan_pday = quan_use / days_supply;

format prev_rx_date date9.;

**run**;

/* build off previous data set, now adding the NEXT value of each

* sort by descending date of service => lag values will be T+1 dispensations */

**proc** **sort** TAGSORT data=prev_tmp1;

by moh_id descending date_of_service descending days_supply;

**run**;

**data** prev_tmp2;

set prev_tmp1;

by moh_id;

/* next variables */

next_days = lag(days_supply);

next_quan = lag(quan_use);

next_rx_date = lag(date_of_service);

next_drug = lag(oat_drug_name);

next_oat_quant = lag(oat_dose_sum);

if first.moh_id then

do;

next_days = **.**;

next_quan = **.**;

next_rx_date = **.**;

next_drug = '';

next_oat_quant = **.**;

end;

next_quan_pday = next_quan / next_days;

/* Some cleaning processes need variables at time T+2 */

next2_quan_pday = lag(next_quan_pday);

next2_rx_date = lag(next_rx_date);

if first.moh_id then

do;

next2_quan_pday = **.**;

next2_rx_date = **.**;

end;

/* calculate period */

period = next_rx_date - date_of_service;

next_period = next2_rx_date - next_rx_date;

/* calculate quantity per day, as it is used frequently as a comparison to the listed quantity */

if next_rx_date ne **.** and period ne **0** then

quan_pd_calc = quan_use / period;

else if period = **0** then

quan_pd_calc = quan_use / days_supply;

if next_rx_date ne **.** and next_period ne **0** and next2_rx_date ne **.** then

next_quan_pd_calc = next_quan / next_period;

/* calculated quantity per day */

if next_quan_pd_calc eq **.** then

next_quan_pd_calc = next_quan_pday;

/* calculated quantity per day at T+2 */

next2_quan_pd_calc = lag(next_quan_pd_calc);

if first.moh_id then

next2_quan_pd_calc = **.**;

format next_rx_date next2_rx_date date9.;

**run**;

**proc** **sort** data=prev_tmp2 TAGSORT out=&data_out.;

by moh_id date_of_service days_supply;

**run**;

/* Delete temporary data sets to save work space */

**proc** **datasets** lib=work nolist;

delete prev_tmp:;

**run**;

**%mend** getPrevNext;

/* Code to clean dispensations for each OAT subgroup

* Following the methods described in Opioid agonist treatment and risk of mortality during opioid overdose public health emergency:

population based retrospective cohort study by Lindsay Pearce et al. and

* Characterizing opioid agonist therapy uptake and factors associated with treatment retention among

people with HIV in British Columbia, Canada by Yazadani et al.

* Building on the code written by Katerina Dolguikh */

**%macro** cleanPnet(drug_grp);

**%macro** ***getColours***;

**%mend** getColours;

/* 1. IDENTIFY SPLIT DOSES */

%***getSplitDose***(&drug_grp., &drug_grp._raw);

/* 2. PNET CLEANING */

%***getPrevNext***(&drug_grp._multi_out, &drug_grp._next);

/* CASE 1: days supply = 0

* Should not apply to Pharmanet data */

**data** &drug_grp._fix1_check;

set &drug_grp._next;

where days_supply = **0**;

**run**;

/* CASE 2: days supply does not equal the time from service date to next service date (days_supply != next Rx date - Rx date)

and quantity/days_supply ne previous quantity/days_supply

* added period ne 0 condition since dispensations with period = 0 uses the same record for previous and next, by the way it is sorted

- so not valuable to use previous and next values for these dispensations

* note that the first dispensation on the day will still have previous/next records as seen if sorted just by moh_id and date of service

* either adjust days_supply (fix 2a) or date_of_service (fix 2b)

* different cases (A1-A4) to ensure certain level of consistency across previous and next records

* otherwise case B: adjust date of service - previous days supply + days supply align with next date of service

* recall: period is based on service dates (next date_of_service - date_of_service)

* note that conditions only depend on quantities dispensed and quantity/day

* want to make updates based on previous fixes - need to update previous variables with fixes

* previous variables that need to be carried forward:

- prev days (changing depending on updates)

- prev quantity (static)

- prev date of service (changing depending on updates, for fix B) */

**/* Fix 2: Days supply and quantity per day mismatch */**

**/* Fix 2: Days supply and quantity per day mismatch */**

**/* Fix 2: Days supply and quantity per day mismatch */**

**/* Fix 2: Days supply and quantity per day mismatch */**

**data** &drug_grp._fix2;

set &drug_grp._next;

by moh_id date_of_service days_supply;

length fix_flag $**100**;

/* carry forward fixes

* these variables will have values from previous dispensations */

retain days_fix2a date_fix2b;

/* update prev_days with fix

* if first moh_id prev_days will be missing

* else set prev_days = days_fix2a which equals the fix from the previous record (since we used retain)

* apply same logic to prev_date */

if first.moh_id then

do;

days_fix2a = **.**;

date_fix2b = **.**;

end;

/* save previous fixes as the new previous values to use in conditions below */

prev_days_new = days_fix2a;

prev_date_new = date_fix2b;

/* since days_fix2a is the fix from the previous record

* lag(days_fix2a) will be the fix from 2 previous records

* set this to prev2_days

* need first.moh_id check to make sure first 2 records have missing prev2_days */

prev2_days_new = lag(days_fix2a);

if first.moh_id then

prev2_days_new = **.**;

/* calculate the fixes

* fix values will now either be updated with corresponding fix or set to the original value

* do not fix non-first records of split doses (split_flag2)

- fix from first record in split dose will be carried forward */

if days_supply ne period and prev_quan/prev_days_new ne quan_use/days_supply

and not first.moh_id and split_flag2 ne **1** and period ne **0** then

do;

flag_error = **1**;

/* A1: calculated quan per day = prev quan per day (21)

* A2: calculated quan per day = prev prev quan per day = calculated next quan per day

OR calculated quan per day = prev prev quan per day = next quan per day (22)

* A3: calculated quan per day = next calculcated quan per day = next next calculated quan per day

OR calculated quan per day = next quan per day = next next quan per day

OR calculated quan per day = calculated next quan per day = next next quan per day

OR calculated quan per day = next quan per day = calculated next next quan per day (23)

* A4: not sure, ignore (24) */

/* A1 */

if quan_pd_calc eq prev_quan/prev_days_new and prev_quan/prev_days_new ne **.** then

do;

days_fix2a = next_rx_date - date_of_service;

fix_flag = catx('/', fix_flag, 'a1');

end;

/* A2 */

else if (quan_pd_calc eq prev2_quan/prev2_days_new eq next_quan_pd_calc and prev2_quan/prev2_days_new ne **.**)

OR (quan_pd_calc eq prev2_quan/prev2_days_new eq next_quan_pday and next_quan_pday ne **.**) then

do;

days_fix2a = next_rx_date - date_of_service;

fix_flag = catx('/', fix_flag, 'a2');

end;

/* A3 */

else if (quan_pd_calc eq next_quan_pd_calc eq next2_quan_pd_calc and next2_quan_pd_calc ne **.**)

OR (quan_pd_calc eq next_quan_pday eq next2_quan_pday and next_quan_pday ne **.**)

OR (quan_pd_calc eq next_quan_pd_calc eq next2_quan_pday and next2_quan_pday ne **.**)

OR (quan_pd_calc eq next_quan_pday eq next2_quan_pd_calc and next_quan_pday ne **.**) then

do;

days_fix2a = next_rx_date - date_of_service;

fix_flag = catx('/', fix_flag, 'a3');

end;

/* no fix, carry forward original days supply */

else days_fix2a = days_supply;

/* B: prev days supply + days supply = next Rx date - prev Rx date (41) */

if prev_days_new + days_supply eq next_rx_date - prev_date_new and prev_date_new ne **.** then

do;

date_fix2b = prev_date_new + prev_days_new;

fix_flag = catx('/', fix_flag, 'b');

end;

/* no fix, carry forward original date of service */

else date_fix2b = date_of_service;

end;

/* no error, carry forward original values */

else

do;

flag_error = **0**;

days_fix2a = days_supply;

date_fix2b = date_of_service;

end;

/* at this point fix values have been updated, but not the previous values */

/* set final days fix and date fix values */

/* carry fix flag / days fix / date fix forward

* to use in other dispensations within a split dose group */

retain prev_fix_flag prev_days_fix prev_date_fix;

format prev_date_fix date9.;

/* pick A if it gets corrected both ways */

if flag_error = **1** and fix_flag ne 'b' and fix_flag ne '' then

do;

days_fix2 = days_fix2a;

date_fix2 = **.**;

end;

else if flag_error = **1** and fix_flag = 'b' then

do;

date_fix2 = date_fix2b;

days_fix2 = **.**;

end;

/* no error or no fix */

else

do;

days_fix2 = **.**;

date_fix2 = **.**;

end;

/* update other dispensations within SPLIT dose group

* split dose group determined by (moh_id, date_of_service, days_supply) */

/* reset if first moh_id or date of service or days supply */

if first.moh_id or first.date_of_service or first.days_supply then

do;

prev_fix_flag = fix_flag;

prev_date_fix = date_fix2;

prev_days_fix = days_fix2;

end;

/* if non-first record within split dose group then update date of service or days supply to fix

* update fix variables as well to carry forward to next dispensation (if same participant) */

else if split_flag = **1** and fix_flag = '' and prev_fix_flag ne '' then

do;

flag_error = **1**;

fix_flag = prev_fix_flag;

if prev_fix_flag = 'b' then

do;

date_fix2 = prev_date_fix; /* final fix variable */

date_fix2b = prev_date_fix; /* previous value for next dispensation */

end;

else if prev_fix_flag ne 'b' then

do;

days_fix2 = prev_days_fix; /* final fix variable */

days_fix2a = prev_days_fix; /* previous value for next dispensation */

end;

end;

/* otherwise set to missing values since values are not needed to carry over */

else

do;

prev_fix_flag = '';

prev_date_fix = **.**;

prev_days_fix = **.**;

end;

format date_fix2b date_fix2 date_of_service date9.;

**run**;

/* Prepare data for fix 3 */

**data** &drug_grp._fix2_tmp;

set &drug_grp._fix2;

drop prev: next:;

rename days_supply=old_days_supply date_of_service=old_date_of_service quan_pday=old_quan_pday fix_flag=old_fix_flag;

**run**;

/* If no fix 2 then set days supply and date of service to original value

* otherwise set days supply / date of service to the corresponding fix

* prioritizing the days supply fix over the date of service fix

* create a new error flag to use as the official error flag variable to carry forward to next fix

* create a new fix flag variable that indicates if fix 2 was applied */

**data** &drug_grp._fix2_out;

set &drug_grp._fix2_tmp;

length fix_flag $**100**;

if old_fix_flag = '' then

do;

days_supply = old_days_supply;

date_of_service = old_date_of_service;

fix_flag = '';

end;

else if days_fix2 ne **.** then

do;

days_supply = days_fix2;

date_of_service = old_date_of_service;

fix_flag = '2';

end;

else if date_fix2 ne **.** then

do;

date_of_service = date_fix2;

days_supply = old_days_supply;

fix_flag = '2';

end;

format date_of_service date9.;

drop days_fix2 date_fix2 days_fix2a date_fix2b old:;

flag_error_new = flag_error;

drop flag_error quan_pd_calc period;

**run**;

/* Reset the previous and next values to use in fix 3 */

%***getPrevNext***(&drug_grp._fix2_out, &drug_grp._next);

**/* Fix 3: Large quantity per day anomalies */**

**/* Fix 3: Large quantity per day anomalies */**

**/* Fix 3: Large quantity per day anomalies */**

**/* Fix 3: Large quantity per day anomalies */**

/* CASE 3: unusually large quantity per day AND short time between Rx

* 39 (B3/B4) typo: Kat changed [NEXT QUAN/(NEXT QUAN/NEXT DAYS)] to [QUAN/(NEXT QUAN/NEXT DAYS)]

* 39 (B3/B4) follow 31/33 and check (days supply fix = quantity_dispensed/next_quan_pd_calc) is an integer */

**data** &drug_grp._fix3;

set &drug_grp._next;

by moh_id date_of_service days_supply;

/* carry fix flag / days fix forward, to use when updating other dispensations within a split dose group */

retain prev_fix_flag prev_days_fix;

/* carry days fix, to use as a previous value */

retain days_fix3;

/* update prev_days with fix

* if first moh_id, prev_days will be missing

* else set previous values to the fix from the previous record (since we used retain)

* to use in conditions below */

if first.moh_id then

days_fix3 = **.**;

prev_days_new = days_fix3;

prev_quan_pday_new = prev_quan/prev_days_new;

/* T-2 variables to use in conditions below */

prev2_days_new = lag(days_fix3);

prev2_quan_pday_new = prev2_quan/prev2_days_new;

if first.moh_id then

do;

prev2_days_new = **.**;

prev2_quan_pday_new = **.**;

end;

/* Fix 3 error condition

* only check if not already fixed by fix 2

* do not check if non-first record in a split dose (split_flag2) */

if abs(quan_pday - prev_quan_pday_new) gt **10** AND abs(quan_pday - next_quan_pd_calc) gt **10**

AND intck('day', prev_rx_date, date_of_service) le **30**

AND intck('day', date_of_service, next_rx_date) le **30**

AND prev_rx_date ne **.** and next_rx_date ne **.** and split_flag2 ne **1** and fix_flag = '' then

do;

/* no error if dose/day is consistent */

if prev_oat_quant/prev_days_new = oat_dose_sum/days_supply = next_oat_quant/next_days then

flag_error = **2**;

else

do;

flag_error = **1**;

/* A1: quan dispensed / prev quan pday is an integer <= 30 AND prev quan pday = next quan pday (31a)

* A2: quan dispensed / prev quan pday is an integer AND prev quan pday = calc next quan pday (31b)

* A3: quantity = prev quantity AND prev quan pday = prev prev quan pday (32)

* A4: quan dispensed / prev quan pday is an integer <= 30 AND prev quan pday = prev prev quan pday (33) */

/* for now prioritize A since it is an integer */

if (quan_use/prev_quan_pday_new eq int(quan_use/prev_quan_pday_new)

AND quan_use/prev_quan_pday_new le **30**

AND prev_quan_pday_new eq next_quan_pday

AND prev_rx_date ne **.** AND next_rx_date ne **.**)

OR (quan_use/prev_quan_pday_new eq int(quan_use / prev_quan_pday_new)

AND prev_quan_pday_new eq next_quan_pd_calc

AND prev_rx_date ne **.** AND next_rx_date ne **.**)

OR (quan_use = prev_quan AND prev_quan_pday_new = prev2_quan_pday_new)

OR (quan_use/prev_quan_pday_new eq int(quan_use/prev_quan_pday_new)

AND quan_use/prev_quan_pday_new le **30**

AND prev_quan_pday_new = prev2_quan_pday_new) then

do;

fix_flag = '3a';

days_fix3a = quan_use / prev_quan_pday_new;

end;

/* B1: quantity = next quantity AND next quan pday calc = next next quan pday calc (38a)

* B2: quantity = next quantity AND next quan pday calc = next next quan pday (38b)

* B3: quantity / next quan pday is an integer AND next quan pday calc = next next quan pday calc (39a)

* B4: quantity / next quan pday is an integer AND next quan pday calc = next next quan pday (39b) */

else if (quan_use eq next_quan AND next_quan_pd_calc eq next2_quan_pd_calc AND next_rx_date ne **.**)

OR (quan_use eq next_quan AND next_quan_pd_calc eq next2_quan_pday AND next_rx_date ne **.** AND next2_quan_pday ne **.**)

OR (quan_use/next_quan_pd_calc eq int(quan_use/next_quan_pd_calc) AND next_quan_pd_calc eq next2_quan_pd_calc

AND next_rx_date ne **.** AND next2_quan_pday ne **.**)

OR (quan_use/next_quan_pd_calc eq int(quan_use/next_quan_pd_calc) AND next_quan_pd_calc eq next2_quan_pday

AND next_rx_date ne **.** AND next2_quan_pday ne **.**) then

do;

fix_flag = '3b';

days_fix3b = quan_use / next_quan_pd_calc;

end;

end;

end;

/* set days fix which previous value will be set to in next iteration */

if fix_flag = '3a' then

days_fix3 = days_fix3a;

else if fix_flag = '3b' then

days_fix3 = days_fix3b;

else days_fix3 = days_supply;

/* now days_fix3 either updated with fix or set to original value

* at this point, different from previous values */

/* set FINAL days fix for clarity */

/* no fix */

if fix_flag ne '3a' and fix_flag ne '3b' then

days_fix3_new = **.**;

/* update with fix */

else days_fix3_new = days_fix3;

/* update other dispensations within SPLIT dose group */

/* reset if first moh_id or date of service or days supply */

if first.moh_id or first.date_of_service or first.days_supply then

do;

prev_fix_flag = fix_flag;

prev_days_fix = days_fix3_new;

end;

/* update days of supply based on previous fixes within SPLIT dose group */

else if split_flag = **1** and fix_flag = '' and prev_fix_flag ne '' then

do;

flag_error = **1**;

fix_flag = prev_fix_flag;

days_fix3_new = prev_days_fix;

days_fix3 = days_fix3_new;

end;

/* otherwise set to missing values */

else

do;

prev_fix_flag = '';

prev_days_fix = **.**;

end;

**run**;

/* Prepare data for fix 4 */

**data** &drug_grp._fix3_tmp;

set &drug_grp._fix3;

drop prev: next:;

rename days_supply=old_days_supply quan_pday=old_quan_pday fix_flag=old_fix_flag;

**run**;

/* If no updates with fix 3, then carry forward days supply from before

* otherwise set it to the updated days supply from fix 3 */

**data** &drug_grp._fix3_out;

set &drug_grp._fix3_tmp;

if days_fix3_new = **.** then do;

days_supply = old_days_supply;

fix_flag = old_fix_flag;

end;

else do;

days_supply = days_fix3_new;

fix_flag = '3';

end;

drop days_fix3: old: ;

if flag_error_new ne **1** and flag_error = **1** then flag_error_new = flag_error;

drop flag_error quan_pd_calc period;

**run**;

/* Reset previous and next values for fix 4 */

%***getPrevNext***(&drug_grp._fix3_out, &drug_grp._next);

**/* Fix 4: Days supply > period */**

**/* Fix 4: Days supply > period */**

**/* Fix 4: Days supply > period */**

**/* Fix 4: Days supply > period */**

/* CASE 4: days supply > period

* unless multiple dispensations on the same day (ie. period = 0) */

**data** &drug_grp._fix4;

set &drug_grp._next;

by moh_id date_of_service days_supply;

/* carry days fix forward to update other dispensations in split dose group */

retain prev_days_fix;

/* carry forward fixes to use as previous values */

retain days_fix4;

if first.moh_id then

days_fix4 = **.**;

/* update prev_days with fix

* if first moh_id, prev_days will be missing

* else set prev_days = days_fix4 which equals the fix from the previous record (since we used retain)

* to use in conditions below */

prev_days_new = days_fix4;

prev_quan_pday_new = prev_quan/prev_days_new;

/* fix 4 error condition

* only check if not already fixed

* do not check if non-first record within a split dose group (split_flag2)

* do not check if period is 0 since fix is setting days suppy to period

- recall there are some dispensations on the same day that are not split doses */

if days_supply gt period and period > **0** and split_flag2 ne **1** and fix_flag = '' then

do;

flag_error = **1**;

/* A1: diff bw quan pday and prev quan pday > 10

AND diff bw quan pday and next quan pday > 10

AND diff bw calc quan pday and prev quan pday <= 10

AND diff bw calc quan pday and next quan pday <= 10 (51) */

if abs(quan_pday - prev_quan_pday_new) gt **10** AND abs(quan_pday - next_quan_pday) gt **10**

AND abs(quan_pd_calc - prev_quan_pday_new) le **10** AND abs(quan_pd_calc - next_quan_pday) le **10** then

do;

days_fix4 = period;

fix_flag = '4';

end;

/* no fix */

else

do;

flag_error = **2**;

days_fix4 = days_supply;

end;

end;

/* no error */

else

do;

days_fix4 = days_supply;

end;

/* days_fix4 now either updated with fix or set to original value

* at this point, different from previous values now */

/* set final days fix variable for clarity */

/* no error or no fix */

if fix_flag ne '4' then

days_fix4_new = **.**;

/* update with fix */

else days_fix4_new = days_fix4;

/* update other dispensations within SPLIT dose group */

/* reset if first moh_id or date of service or days supply */

if first.moh_id or first.date_of_service or first.days_supply then

do;

prev_days_fix = days_fix4_new;

end;

/* update days of supply based on previous fix within SPLIT dose group */

else if split_flag = **1** and flag_error ne **1** and prev_days_fix ne **.** then

do;

flag_error = **1**;

days_fix4_new = prev_days_fix;

days_fix4 = days_fix4_new;

end;

/* otherwise set to missing values */

else

do;

prev_days_fix = **.**;

end;

**run**;

**/* Fix 5 and 6: Remaining quantity per day outliers*/**

**/* Fix 5 and 6: Remaining quantity per day outliers*/**

**/* Fix 5 and 6: Remaining quantity per day outliers*/**

**/* Fix 5 and 6: Remaining quantity per day outliers*/**

/* Prepare data for fix 5 */

**data** &drug_grp._fix4_tmp;

set &drug_grp._fix4;

drop prev: next:;

rename days_supply=old_days_supply quan_pday=old_quan_pday fix_flag=old_fix_flag;

**run**;

/* If no fix 4 then carry forward days supply value from before

* otherwise update with fix */

**data** &drug_grp._fix4_out;

set &drug_grp._fix4_tmp;

if days_fix4_new = **.** then do;

days_supply = old_days_supply;

fix_flag = old_fix_flag;

end;

else do;

days_supply = days_fix4_new;

fix_flag = '4';

end;

drop days_fix4: old: ;

if flag_error_new ne **1** and flag_error = **1** then flag_error_new = flag_error;

drop flag_error quan_pd_calc period;

**run**;

/* Reset previous and next values to use in fix 5 */

%***getPrevNext***(&drug_grp._fix4_out, &drug_grp._next);

/* EXTRA FIX (by Katerina Dolguikh): If quantity or days supply stands out (eg. 11 mg, but 110 mg in surrounding doses, or 47 days where it’s been consistently 4 days) recalculate erroneous ones based on the previous quantity per day and whichever field is not an error*/

**data** &drug_grp._fix5;

set &drug_grp._next;

by moh_id date_of_service days_supply;

/* carry fix flag / days fix / quantity fix forward

* to update other dispensations within same split dose group */

retain prev_days_fix prev_quan_fix prev_fix_flag;

/* fix variables */

retain days_fix5 quan_fix5;

if first.moh_id then

do;

days_fix5 = **.**;

quan_fix5 = **.**;

prev_oat_quant = **.**;

end;

/* update prev_days with fix

* previous values to use in conditions below */

prev_days_new = days_fix5;

prev_quan_new = quan_fix5;

prev_quan_pday_new = prev_quan_new/prev_days_new;

/* T-2 variables */

prev2_days_new = lag(days_fix5);

prev2_quan_new = lag(quan_fix5);

prev2_oat_quant = lag(prev_oat_quant);

if first.moh_id then

do;

prev2_days_new = **.**;

prev2_quan_new = **.**;

prev2_oat_quant = **.**;

end;

prev2_quan_pday_new = prev2_quan_new/prev2_days_new;

/* T-3 variables */

prev3_quan_pday_new = lag(prev2_quan_pday_new);

if first.moh_id then

prev3_quan_pday_new = **.**;

/* if previous and next quantity per day are consistent and quantity per day stands out

* quantity per day not equal to previous quantity per day

* only check for fix if no fix has been applied yet

* skip if a non-first record within a split dose group (split_flag2) */

if quan_pday ne prev_quan_pday_new and not first.moh_id and

prev_quan_pday_new eq prev2_quan_pday_new eq prev3_quan_pday_new

and prev_quan_pday_new eq next_quan_pday and split_flag2 ne **1** and fix_flag = '' then

do;

flag_error = **1**;

/* if days supply differ and previous quantity per day and quantity per day differ by > 20 */

if days_supply ne prev_days_new and quan_use eq prev_quan_new

and abs(quan_pday - prev_quan_pday_new) gt **20** then

r = **1**;

/* otherwise compare with T-2 variables */

else if days_supply ne prev2_days_new and quan_use eq prev2_quan_new

and abs(quan_pday - prev2_quan_pday_new) gt **20** and prev2_quan_pday_new ne **.** then

s = **1**;

/* otherwise if quantities differ and previous quantity per day and quantity per day differ by > 20 */

else if days_supply eq prev_days_new and quan_use ne prev_quan_new

and abs(quan_pday - prev_quan_pday_new) gt **20** then

do;

/* if a split dose then do not change the quantity dispensed */

if split_flag = **1** then

do;

flag_error = **2**;

p = **3**;

end;

/* otherwise if OAT dose is consistent do not change */

else if oat_dose_sum = prev_oat_quant then

do;

flag_error = **2**;

p = **2**;

end;

else p = **1**;

end;

/* otherwise compare with T-2 variables */

else if days_supply eq prev2_days_new and quan_use ne prev2_quan_new

and abs(quan_pday - prev2_quan_pday_new) gt **20** and prev2_quan_pday_new ne **.** then

do;

if split_flag = **1** then

do;

flag_error = **2**;

q = **3**;

end;

else if oat_dose_sum = prev2_oat_quant then

do;

flag_error = **2**;

q = **2**;

end;

else q = **1**;

end;

end;

/* change quantity dispensed */

if p eq **1** or q eq **1** then

do;

quan_fix5 = prev_quan_pday_new * days_supply;

fix_flag = '5a';

end;

else quan_fix5 = quan_use;

/* change days supply */

if r eq **1** or s eq **1** then

do;

days_fix5 = quan_use * (**1** / prev_quan_pday_new);

fix_flag = '5b';

end;

else days_fix5 = days_supply;

/* if days supply within range of integer then round days supply

* otherwise do not use fix */

if abs(days_fix5 - round(days_fix5, **1**)) le **0.2** then

days_fix5 = round(days_fix5, **1**);

else

do;

days_fix5 = days_supply;

flag_error = **2**;

fix_flag = '';

end;

/* no error, so no fix5 */

if fix_flag ne '5a' and fix_flag ne '5b' then

do;

days_fix5_new = **.**;

quan_fix5_new = **.**;

end;

/* otherwise update final fix5 variables */

else

do;

if r eq **1** or s eq **1** then

do;

fix_flag = '5a';

days_fix5_new = days_fix5;

end;

if p eq **1** or q eq **1** then

do;

fix_flag = '5b';

quan_fix5_new = quan_fix5;

end;

end;

/* update other dispensations within SPLIT dose group */

/* reset if first moh_id or date of service */

if first.moh_id or first.date_of_service or first.days_supply then

do;

prev_days_fix = days_fix5_new;

prev_quan_fix = quan_fix5_new;

prev_fix_flag = fix_flag;

end;

/* update days of supply based on previous fixes within SPLIT dose group */

else if split_flag = **1** and flag_error ne **1** and prev_fix_flag ne '' then

do;

flag_error = **1**;

fix_flag = prev_fix_flag;

/* fix days supply */

if prev_fix_flag = '5a' then

do;

days_fix5_new = prev_days_fix;

days_fix5 = days_fix5_new;

end;

/* or quantity dispensed */

else if prev_fix_flag = '5b' then

do;

quan_fix5_new = prev_quan_fix;

quan_fix5 = quan_fix5_new;

end;

end;

/* otherwise split flag but no fix from previous dispensation

* should set to missing values */

else

do;

prev_days_fix = **.**;

prev_quan_fix = **.**;

prev_fix_flag = '';

end;

**run**;

**data** &drug_grp._fix5_tmp;

set &drug_grp._fix5;

drop prev: next: r s p q;

rename days_supply=old_days_supply quantity_dispensed=old_quantity_dispensed quan_pday=old_quan_pday fix_flag=old_fix_flag;

**run**;

/* If no fix 5 then carry forward days supply and quantity dispensed value from before

* otherwise update with fix */

**data** &drug_grp._fix5_out;

set &drug_grp._fix5_tmp;

if days_fix5_new ne **.** then do;

days_supply = days_fix5_new;

fix_flag = '5';

quantity_dispensed = old_quantity_dispensed;

end;

else if quan_fix5_new ne **.** then do;

quantity_dispensed = quan_fix5_new;

fix_flag = '5';

days_supply = old_days_supply;

end;

else do;

days_supply = old_days_supply;

quantity_dispensed = old_quantity_dispensed;

fix_flag = old_fix_flag;

end;

drop days_fix5: quan_fix5: ;

if flag_error_new ne **1** and flag_error = **1** then flag_error_new = flag_error;

drop flag_error;

**run**;

/* final cleaned pharmanet data */

**data** &drug_grp._fix_out;

set &drug_grp._fix5_out;

if flag_error_new ne **1** then no_fix = **9**;

/* otherwise no fix for error */

else if flag_error_new = **1** and fix_flag = '' then no_fix = **1**;

else no_fix = **0**;

rename split_flag = split_flag_old;

drop quan_use oat_dose_sum;

**run**;

**proc** **freq** data=&drug_grp._fix_out;

tables flag_error_new no_fix flag_error_new*no_fix;

format flag_error_new flag_error_fmt. no_fix no_fix_fmt.;

**run**;

/* 3. Re-identify split doses */

**proc** **sort** data=&drug_grp._fix_out;

by moh_id date_of_service days_supply;

**run**;

%***getSplitDose***(&drug_grp._fix, &drug_grp._fix_out);

/* Delete temporary data sets to save work space */

**proc** **datasets** lib=work nolist;

delete &drug_grp._fix1: &drug_grp._fix2: &drug_grp._fix3: &drug_grp._fix4: &drug_grp._fix5: ;

**run**;

**%mend** cleanPnet;

/* A Methodological Framework for Constructing Opioid Agonist Therapy Episodes in Administrative Data: Insights and Key Challenges

* Researcher: Kiana Yazdani

* Analyst: Cassidy Tam

* Related to request 434: Impact of stimulant use disorder on outcomes related to opioid agonist therapy in people living with HIV

* June/03/2025 */

/* This code implements the Pharamanet cleaning code in 05a_pnet_clean_macro.sas

* to Pharmanet records for the cohort described in request 434 and during the request 434 study period */

%include "&SAS_path\05a_pnet_clean_macro.sas";

/* Load OAT information supplied by Kiana Yazdani for request 434 */

options validvarname=v7;

**data** ref_codes(compress=y);

set ref.OAT;

rename Flag_1 = oat_drug_type Flag_2 = oat_drug_quant_str;

oat_drug_name = compbl(translate(OAT_Brand_Name,' ','A0'x));

length oat_drug_gen $ **100**;

din_pin = input(DINPIN__PharmaNet_, **10.**);

if find(Flag_1, 'MTD') then

oat_drug_gen = 'Methadone';

else if find(Flag_1, 'Kadian') then

oat_drug_gen = 'SROM';

else if find(Flag_1, 'M-Eslon') then

oat_drug_gen = 'SROM';

else if find(Flag_1, 'Bup') then

oat_drug_gen = 'Buprenorphine';

else if find(Flag_1, 'iOAT') then

oat_drug_gen = 'Injective OAT';

if find(Flag_2, 'm') ne **0** then

oat_drug_quant = input(substr(Flag_2, **1**, find(Flag_2, 'm')-**1**), best.);

where Flag_1 ne '';

keep oat_drug_name Flag_1 Flag_2 oat_drug_gen din_pin oat_drug_quant;

**proc** **sort**;

by din_pin;

**run**;

/* remove duplicates */

**proc** **sort** data=ref_codes out=ref_codes_nodup nodupkey;

by din_pin oat_drug_name;

**run**;

* Katerina Dolguikh removed Pharmanet records with no date or negative cost;

**proc** **sql** noprint;

create table pharmanet as

select *

from moh.pnet

where din_pin in (select distinct din_pin from ref_codes_nodup)

and (total_amount_claimed ge **0** or total_amount_claimed eq **.**) and date_of_service ne **.**;

**quit**;

**proc** **contents** data=pharmanet;

**run**;

* All OAT dispensations;

**proc** **sql**;

create table oat_raw as

select a.moh_id, a.prac_moh_key, a.days_supply, a.din_pin, b.oat_drug_name,

a.date_of_service, a.quantity_dispensed, b.oat_drug_gen, b.oat_drug_type, b.oat_drug_quant,

a.drug_strength, a.drug_brand_name, a.drug_generic_name

from pharmanet a

left join ref_codes_nodup b on a.din_pin = b.din_pin

order by a.moh_id, a.date_of_service, b.oat_drug_gen, b.oat_drug_name, b.oat_drug_type, a.quantity_dispensed, a.days_supply;

**quit**;

**proc** **freq** data=oat_raw;

tables oat_drug_gen;

**run**;

/* fill in missing oat drug quantity for Methadose (Methadone HCL) */

**proc** **freq** data=oat_raw(where=(oat_drug_quant = **.**));

tables drug_strength;

**run**;

**data** oat_raw;

set oat_raw;

if oat_drug_quant = **.** then

oat_drug_quant = input(substr(drug_strength, **1**, **2**), best.);

**run**;

* load cohort for request 434;

**data** cohort;

set myref.edar434_cohort;

**run**;

* All OAT dispensations for cohort during study period;

**proc** **sql**;

create table cohort_oat_raw as

select a.moh_id, a.oud, a.stud, a.oud_stud, b.*

from cohort a

inner join oat_raw(rename=moh_id=b_moh_id) b on a.moh_id = b.b_moh_id and b.date_of_service>=&study_start_dt. and date_of_service <= &study_end_dt.

;

**quit**;

**proc** **sql**;

create table cohort_oat as

select distinct moh_id

from cohort_oat_raw;

**quit**;

/* SPLIT UP OAT_RAW BY OAT DRUG GENERAL GROUP AND THEN CLEAN WITHIN EACH GROUP

- avoids comparing quantities of different drugs */

/* delete Butrans - Buprenorphine patch */

**data** bup_raw;

set cohort_oat_raw;

where oat_drug_gen = 'Buprenorphine';

oat_type = **1**;

if find(oat_drug_type, 'bup_butrans', 'i') then

delete;

**run**;

/* delete yearly iOAT dispensations */

**data** ioat_raw;

set cohort_oat_raw;

where oat_drug_gen = 'Injective OAT';

oat_type = **2**;

if days_supply = **365** then

delete;

**run**;

**data** ioat_yearly;

set cohort_oat_raw;

where oat_drug_gen = 'Injective OAT';

oat_type = **2**;

if days_supply = **365** then

output;

**run**;

**data** srom_raw;

set cohort_oat_raw;

where oat_drug_gen = 'SROM';

oat_type = **3**;

**run**;

**data** meth_raw;

set cohort_oat_raw;

where oat_drug_gen = 'Methadone';

oat_type = **4**;

**run**;

/* 1. Buprenorphine */

%let drug_grp = bup;

%***cleanPnet***(bup);

/* 2. iOAT */

%let drug_grp = ioat;

%***cleanPnet***(ioat);

/* 3. SROM */

%let drug_grp = srom;

%***cleanPnet***(srom);

/* 4. Methadone */

%let drug_grp = meth;

%***cleanPnet***(meth);

/* merge fixes */

**data** oat_fix;

set bup_fix_multi_out ioat_fix_multi_out srom_fix_multi_out meth_fix_multi_out;

by moh_id date_of_service days_supply;

dose_pday = oat_dose_sum / days_supply;

label dose_pday = 'Dose/day'

days_supply = 'Days supply'

oat_drug_gen = 'OAT type'

quantity_dispensed = 'Quantity dispensed';

**run**;

/* A Methodological Framework for Constructing Opioid Agonist Therapy Episodes in Administrative Data: Insights and Key Challenges

* Researcher: Kiana Yazdani

* Analyst: Cassidy Tam

* Related to request 434: Impact of stimulant use disorder on outcomes related to opioid agonist therapy in people living with HIV

* June/03/2025 */

/* PHARMANET VARIABLES:

- days_supply = estimated/intended number of days product should last for

- quantity_dispensed = total amount of product dispensed

- date_of_service = drug dispensation date

- moh_id = unique participant ID

* VARIABLES following reference documents from Kiana Yazdani for request 434:

- oat_type = numeric variable indicating OAT type:

1 = Buprenorphine

2 = iOAT

3 = Kadian and M-Eslon (SROM)

4 = Methadone

- oat_drug_gen = OAT type: Buprenorphine, iOAT, Kadian and M-Eslon (SROM), Methadone

- oat_drug_type = OAT brand name abbreviation

- oat_drug_name = OAT brand name

- oat_drug_quant = Quantity of OAT in given drug */

/* Code to count how each dispensation (disp2) relates to the previous dispensation (disp1)

* Allen left-side temporal relations:

1. disp1 before disp2: disp1_end < disp2_start (disp1, disp2 can be 1 day)

2. disp1 meets disp2: disp1_end = disp2_start (disp1, disp2 can be 1 day)

3. disp1 overlaps disp2: disp1_start < disp2_start < disp1_end < disp2_end => disp1, disp2 > 1 day

4. disp1 is finished by disp2: disp1_start < disp2_start < disp2_end = disp1_end => disp1, disp2 > 1 day

5. disp1 contains disp2: disp1_start < disp2_start < disp2_end < disp1_end => disp1, disp2 > 1 day

6. disp1 starts disp2: disp1_start = disp2_start < disp1_end < disp2_end => disp1, disp2 > 1 day

7. disp1 equals disp2: disp1_start = disp2_start and disp2_end = disp2_end (disp1, disp2 can be 1 day) */

**%macro** count_relations(drug_grp);

**%macro** ***getColours***;

**%mend** getColours;

/* calculate dispensation end date */

**data** count_&drug_grp._tmp1;

retain moh_id oat_type oat_drug_gen oat_drug_type din_pin oat_drug_name oat_drug_quant quantity_dispensed oat_dose_sum days_supply

date_of_service end_date_service;

set &drug_grp._fix_multi_out;

end_date_service = date_of_service + days_supply - **1**;

format end_date_service date9.;

keep moh_id oat_type oat_drug_gen oat_drug_type din_pin oat_drug_name date_of_service end_date_service days_supply quantity_dispensed

oat_drug_quant oat_dose_sum split_flag;

if date_of_service>=&study_start_dt. and date_of_service <= &study_end_dt.;

**proc** **sort**;

by moh_id date_of_service end_date_service;

**run**;

**data** count_&drug_grp._tmp2;

set count_&drug_grp._tmp1;

by moh_id;

prev_start = lag(date_of_service);

prev_end = lag(end_date_service);

format prev_start prev_end date9.;

if first.moh_id then

do;

prev_start = **.**;

prev_end = **.**;

relation_flag = **0**;

end;

retain count_before count_meet count_overlap count_finish count_contain count_start count_equal count_split count_flag;

if _n_ = **1** then

do;

count_before = **0**;

count_meet = **0**;

count_overlap = **0**;

count_finish = **0**;

count_contain = **0**;

count_start = **0**;

count_equal = **0**;

count_split = **0**;

count_flag = **0**;

end;

if prev_start ne **.** and prev_end ne **.** then

do;

/* split dose */

if prev_start = date_of_service and prev_end = end_date_service and split_flag = **1** then

do;

count_split = count_split + **1**;

relation_flag = **1**;

end;

/* 7. disp1 equals disp2: disp1_start = disp2_start and disp2_end = disp2_end (disp1, disp2 can be 1 day) */

else if prev_start = date_of_service and prev_end = end_date_service and split_flag ne **1** then

do;

count_equal = count_equal + **1**;

relation_flag = **2**;

end;

/* 6. disp1 starts disp2: disp1_start = disp2_start < disp1_end < disp2_end => disp1, disp2 > 1 day */

else if prev_start = date_of_service and date_of_service < prev_end and prev_end < end_date_service then

do;

count_start = count_start + **1**;

relation_flag = **3**;

end;

/* 5. disp1 contains disp2: disp1_start < disp2_start <= disp2_end < disp1_end => disp1 > 1 day, disp2 can be 1 day */

else if prev_start < date_of_service and end_date_service < prev_end then

do;

count_contain = count_contain + **1**;

relation_flag = **4**;

end;

/* 4. disp1 is finished by disp2: disp1_start < disp2_start < disp2_end = disp1_end => disp1, disp2 > 1 day */

else if prev_start < date_of_service and date_of_service < end_date_service and prev_end = end_date_service then

do;

count_finish = count_finish + **1**;

relation_flag = **5**;

end;

/* 3. disp1 overlaps disp2: disp1_start < disp2_start < disp1_end < disp2_end => disp1, disp2 > 1 day */

else if prev_start < date_of_service and date_of_service < prev_end and prev_end < end_date_service then

do;

count_overlap = count_overlap + **1**;

relation_flag = **6**;

end;

/* 2. disp1 meets disp2: disp1_end = disp2_start (disp1, disp2 can be 1 day) */

else if prev_end = date_of_service then

do;

count_meet = count_meet + **1**;

relation_flag = **7**;

end;

/* 1. disp1 before disp2: disp1_end < disp2_start (disp1, disp2 can be 1 day) */

else if prev_end < date_of_service then

do;

count_before = count_before + **1**;

relation_flag = **8**;

end;

/* catch errors */

else

do;

count_flag = count_flag + **1**;

relation_flag = **9**;

end;

end;

**run**;

**proc** **sql**;

create table count_&drug_grp._tmp3 as

select max(count_before) as max_count_before, max(count_meet) as max_count_meet, max(count_overlap) as max_count_overlap,

max(count_finish) as max_count_finish, max(count_contain) as max_count_contain, max(count_start) as max_count_start,

max(count_equal) as max_count_equal, max(count_split) as max_count_split, max(count_flag) as max_count_flag,

count (distinct moh_id) as num_participants

from count_&drug_grp._tmp2;

**quit**;

**data** count_&drug_grp._out;

set count_&drug_grp._tmp3;

total_rec = sum(max_count_before, max_count_meet, max_count_overlap, max_count_finish, max_count_contain, max_count_start,

max_count_equal, max_count_split, max_count_flag, num_participants);

**run**;

**data** relation_&drug_grp._out;

set count_&drug_grp._tmp2;

drop count:;

**run**;

**proc** **freq** data=relation_&drug_grp._out;

tables relation_flag oat_drug_type;

format relation_flag relation_fmt.;

**run**;

/* Delete temporary data sets to save work space */

**proc** **datasets** lib=work nolist;

delete count_&drug_grp._tmp:;

**run**;

**%mend** count_relations;

/* Code to aggregate nested dispensations */

**%macro** add_nested_rx(drug_grp);

**%macro** ***getColours***;

**%mend** getColours;

**proc** **sort** data=relation_&drug_grp._out(drop=prev_start prev_end) out=nest_&drug_grp._tmp1;

by moh_id date_of_service descending end_date_service;

**run**;

/* create groups of dispensations that will be aggregated

* recall: if one day dispensation at start or end of other dispensation then considered meets

* use start_dt, end_dt to keep track of minimum start date and maximum end date

* use interval_start and interval_end to create interval groupings

* to avoid grouping in the 1-day meets dispensations */

**data** nest_&drug_grp._tmp2;

set nest_&drug_grp._tmp1;

by moh_id;

retain start_dt end_dt interval_start interval_end;

format start_dt end_dt interval_start interval_end date9.;

if first.moh_id then

do;

start_dt = date_of_service;

end_dt = end_date_service;

end;

else if end_date_service > end_dt then

do;

start_dt = date_of_service;

end_dt = end_date_service;

end;

if (date_of_service = end_dt and end_date_service = end_dt)

or (date_of_service = start_dt and end_date_service = start_dt) then

do;

interval_start = date_of_service;

interval_end = end_date_service;

end;

else

do;

interval_start = start_dt;

interval_end = end_dt;

end;

**proc** **sort**;

by moh_id interval_start interval_end date_of_service descending end_date_service;

**run**;

/* create new start and stop dates based on overlap

* within each interval group, dispensations are sorted by date_of_service and descending end_date_service

* later (or new, since orderded by increasing date_of_service) date_of_service dates within an interval group indicates a new STOP date

- new overlap starting at date_of_service

- previous overlap period stops at (date_of_service - 1)

* earlier (or new, since ordered by descending end_date_service) end_date_of_service within an interval group indicates a new START date

- new overlap starts at (end_date_service + 1)

- previous overlap period ends at end_date_service */

**data** nest_&drug_grp._tmp3;

set nest_&drug_grp._tmp2;

by moh_id interval_start interval_end;

format start_new stop_new date9.;

/* if new interval reset */

if first.interval_start or first.interval_end then

do;

start_new = **.**;

stop_new = **.**;

end;

else

do;

start_new = end_date_service + **1**;

stop_new = date_of_service - **1**;

end;

if start_new < interval_start or start_new > interval_end then

start_new = **.**;

if stop_new < interval_start or stop_new > interval_end then

stop_new = **.**;

keep moh_id din_pin interval_start interval_end date_of_service end_date_service start_new stop_new;

**run**;

/* get all distinct start dates */

**proc** **sql**;

create table nest_&drug_grp._tmp4a as

select distinct moh_id, interval_start, interval_end, date_of_service

from nest_&drug_grp._tmp3

union

select distinct moh_id, interval_start, interval_end, start_new

from nest_&drug_grp._tmp3

where start_new ne **.**

order by moh_id, interval_start, interval_end, date_of_service;

**quit**;

/* create row_id to keep order when merging with stop dates */

**data** nest_&drug_grp._tmp4a;

set nest_&drug_grp._tmp4a;

by moh_id interval_start interval_end;

retain row_id;

if first.interval_start or first.interval_end then

row_id = **1**;

else row_id = row_id + **1**;

**run**;

/* get all distinct end dates */

**proc** **sql**;

create table nest_&drug_grp._tmp4b as

select distinct moh_id, interval_start, interval_end, end_date_service

from nest_&drug_grp._tmp3

union

select distinct moh_id, interval_start, interval_end, stop_new

from nest_&drug_grp._tmp3

where stop_new ne **.**

order by moh_id, interval_start, interval_end, end_date_service;

**quit**;

/* create row_id to keep order when merging with start dates */

**data** nest_&drug_grp._tmp4b;

set nest_&drug_grp._tmp4b;

by moh_id interval_start interval_end;

retain row_id;

if first.interval_start or first.interval_end then

row_id = **1**;

else row_id = row_id + **1**;

**run**;

/* merge start and stop dates

* there should be the same number of start and stop dates */

**proc** **sql**;

create table nest_&drug_grp._tmp5 as

select a.moh_id, a.row_id, a.interval_start, a.interval_end, a.date_of_service as date_of_service_new,

b.end_date_service as end_date_service_new

from nest_&drug_grp._tmp4a a

left join nest_&drug_grp._tmp4b b on a.moh_id = b.moh_id and a.row_id = b.row_id and a.interval_start = b.interval_start and

a.interval_end = b.interval_end

order by a.moh_id, a.interval_start, a.interval_end, a.row_id;

**quit**;

/* join back drug information */

**proc** **sql**;

create table nest_&drug_grp._tmp6 as

select a.moh_id, a.oat_type, a.oat_drug_gen, a.oat_drug_type, a.din_pin, a.oat_drug_name, a.oat_drug_quant, a.quantity_dispensed,

a.days_supply, a.date_of_service, a.end_date_service, a.interval_start, a.interval_end,

b.date_of_service_new, b.end_date_service_new

from nest_&drug_grp._tmp2 a

left join nest_&drug_grp._tmp5 b on a.moh_id = b.moh_id and a.interval_start = b.interval_start and a.interval_end = b.interval_end

and a.date_of_service <= b.date_of_service_new and b.end_date_service_new <= a.end_date_service

order by a.moh_id, a.interval_start, a.interval_end, b.date_of_service_new, b.end_date_service_new;

**quit**;

/* add OAT dose across each [date_of_service_new, end_date_service_new] interval */

**data** nest_&drug_grp._tmp7;

set nest_&drug_grp._tmp6;

by moh_id interval_start interval_end date_of_service_new end_date_service_new;

length oat_drugs $100.;

retain oat_dose_sum dose_pday_sum oat_drugs;

days_supply_new = end_date_service_new - date_of_service_new + **1**;

if first.moh_id or first.interval_start or first.interval_end

or first.date_of_service_new or first.end_date_service_new then

do;

dose_pday_sum = oat_drug_quant*quantity_dispensed/days_supply;

oat_dose_sum = dose_pday_sum*days_supply_new;

oat_drugs = oat_drug_type;

end;

/* catch Hydro + Diac combined exception - just use Hydro dispensation */

else if oat_drug_type = 'iOAT_Hydr' and oat_drugs = 'iOAT_Diac' then

do;

dose_pday_sum = oat_drug_quant*quantity_dispensed/days_supply;

oat_dose_sum = dose_pday_sum*days_supply_new;

oat_drugs = oat_drug_type;

end;

/* otherwise concatenate */

else

do;

dose_pday_sum = dose_pday_sum + oat_drug_quant*quantity_dispensed/days_supply;

oat_dose_sum = dose_pday_sum*days_supply_new;

if find(oat_drugs, oat_drug_type, 'i') = **0** then

oat_drugs = catx(' / ', oat_drugs, oat_drug_type);

end;

**run**;

/* last date_of_service_new or end_date_service_new of each interval [interval_start, interval_end] (created based on nested dispensations)

* has the final dose_pday and oat_dose for the interval

* note that [date_of_service_new, end_date_service_new] are not distinct across [interval_start, interval_end] groups

* in the case that there is partial overlap across between groups of fully nested dispensations (ie. [interval_start, interval_end]) */

**data** nest_&drug_grp._out;

set nest_&drug_grp._tmp7;

by moh_id interval_start interval_end date_of_service_new end_date_service_new;

drop oat_drug_type din_pin oat_drug_name oat_drug_quant quantity_dispensed days_supply date_of_service end_date_service;

if last.date_of_service_new or last.end_date_service_new;

**run**;

**proc** **freq** data=nest_&drug_grp._out;

tables oat_drugs /nocum;

**run**;

**proc** **sql**;

select max(length(oat_drugs)) as oat_drugs_length

from nest_&drug_grp._out;

**quit**;

/* Delete temporary data sets to save work space */

**proc** **datasets** lib=work nolist;

delete nest_&drug_grp._tmp:;

**run**;

**%mend** add_nested_rx;

/* Code to shift date of service for overlapping dispensations

* shift date = max(date of service, previous shifted date + previous days supply)

* Only shift if overlap is less than epsilon days (eps)

* check overlap based on shifted dates

* beta can be used to cap number of days a date is shifted by

* but if only shifting if overlap is <= eps then maximum shifting should be eps */

**%macro** shift_rx(drug_grp, eps);

**%macro** ***getColours***;

**%mend** getColours;

**proc** **sort** data=nest_&drug_grp._out;

by moh_id date_of_service_new end_date_service_new days_supply_new;

**run**;

**data** shift_&drug_grp._tmp;

set nest_&drug_grp._out;

by moh_id date_of_service_new end_date_service_new;

/* using retain, so date_of_service_shift and end_date_service_shift will be the previous row

* updated accordingly after overlap conditions are checked between current start date (date_of_service_new)

* and previous end date (end_date_service_shift) */

retain date_of_service_shift end_date_service_shift;

format date_of_service_shift end_date_service_shift date9.;

/* carry forward shifted end date */

days_fix_prev = ifn(first.moh_id, **.**, lag(days_supply_new));

/* if overlap then calculate days based on previous shifted dates */

if first.moh_id then

overlap_days = **.**;

else if date_of_service_new <= end_date_service_shift then

overlap_days = end_date_service_shift - date_of_service_new + **1**;

else overlap_days = **.**;

if first.moh_id then

do;

date_of_service_shift = date_of_service_new;

end_date_service_shift = end_date_service_new;

end;

/* if no epsilon criteria then shift regardless */

else if &eps. = **0** then

date_of_service_shift = max(date_of_service_new, end_date_service_shift + **1**);

/* otherwise if overlap < epsilon then shift start date if necessary */

else if **0** <= overlap_days <= &eps. then

date_of_service_shift = max(date_of_service_new, end_date_service_shift + **1**);

/* otherwise keep as is */

else date_of_service_shift = date_of_service_new;

/* update end date based on shifted date of service */

end_date_service_shift = date_of_service_shift + days_supply_new - **1**;

/* keep track of shifting */

shift_diff_start = abs(date_of_service_shift - date_of_service_new);

shift_diff_end = abs(end_date_service_shift - end_date_service_new);

drop days_fix_prev;

**run**;

**proc** **sort** data=shift_&drug_grp._tmp out=shift_&drug_grp._out;

by moh_id date_of_service_shift end_date_service_shift;

**run**;

**proc** **tabulate** data=shift_&drug_grp._out;

var shift_diff_start shift_diff_end;

table shift_diff_start shift_diff_end, n nmiss mean median mode min max Q1 Q3;

**quit**;

/* Delete temporary data sets to save work space */

**proc** **datasets** lib=work nolist;

delete shift_&drug_grp._tmp:;

**run**;

**%mend** shift_rx;

/* A Methodological Framework for Constructing Opioid Agonist Therapy Episodes in Administrative Data: Insights and Key Challenges

* Researcher: Kiana Yazdani

* Analyst: Cassidy Tam

* Related to request 434: Impact of stimulant use disorder on outcomes related to opioid agonist therapy in people living with HIV

* June/03/2025 */

/* Code that shifts overlapping dispensations based on original dispensation dates

* Used in exploratory work, generates tables in appendix 5 */

/* Code to shift date of service for overlapping dispensations

* shift date = max(date of service, previous shifted date + previous days supply)

* Only shift if overlap is less than epsilon days (eps)

* Overlap calculated using original dispensation dates */

**%macro** shift_rx_explore(drug_grp, eps);

**%macro** ***getColours***;

**%mend** getColours;

**proc** **sort** data=nest_&drug_grp._out;

by moh_id date_of_service_new end_date_service_new days_supply_new;

**run**;

**data** shift_eps&eps._&drug_grp._tmp;

set nest_&drug_grp._out;

by moh_id date_of_service_new end_date_service_new;

/* using retain, so date_of_service_shift and end_date_service_shift will be the previous row */

retain date_of_service_shift end_date_service_shift;

format date_of_service_shift end_date_service_shift prev_end date9.;

/* carry forward shifted end date */

days_fix_prev = ifn(first.moh_id, **.**, lag(days_supply_new));

prev_end = ifn(first.moh_id, **.**, lag(end_date_service_new));

/* if overlap then calculate days based on previous dates */

if first.moh_id then

overlap_days = **.**;

else if date_of_service_new <= prev_end then

overlap_days = prev_end - date_of_service_new + **1**;

else overlap_days = **.**;

if first.moh_id then

do;

date_of_service_shift = date_of_service_new;

end_date_service_shift = end_date_service_new;

end;

/* if no epsilon criteria then shift regardless */

else if &eps. = **0** then

date_of_service_shift = max(date_of_service_new, end_date_service_shift + **1**);

/* otherwise if overlap < epsilon then shift start date */

else if **0** < overlap_days <= &eps. then

date_of_service_shift = max(date_of_service_new, end_date_service_shift + **1**);

/* otherwise keep as is */

else date_of_service_shift = date_of_service_new;

/* update end date based on shifted date of service */

end_date_service_shift = date_of_service_shift + days_supply_new - **1**;

/* keep track of shifting */

shift_diff = abs(date_of_service_shift - date_of_service_new);

shift_diff_end = abs(end_date_service_shift - end_date_service_new);

drop days_fix_prev;

**run**;

**proc** **sort** data=shift_eps&eps._&drug_grp._tmp out=shift_eps&eps._&drug_grp._out;

by moh_id date_of_service_shift end_date_service_shift;

**run**;

title "&drug_grp., eps = &eps.";

**proc** **tabulate** data=shift_eps&eps._&drug_grp._out;

var shift_diff;

table shift_diff, n nmiss mean median mode min max Q1 Q3;

**quit**;

title;

/* Delete temporary data sets to save work space */

**proc** **datasets** lib=work nolist;

delete shift_eps&eps._&drug_grp._tmp:;

**run**;

**%mend** shift_rx_explore;

/* A Methodological Framework for Constructing Opioid Agonist Therapy Episodes in Administrative Data: Insights and Key Challenges

* Researcher: Kiana Yazdani

* Analyst: Cassidy Tam

* Related to request 434: Impact of stimulant use disorder on outcomes related to opioid agonist therapy in people living with HIV

* June/03/2025 */

/* Implements code found in 05c_overlap_rx_macro

* Handles fully nested and partially overlapping dispensations

* Even after fixing process is applied, overlap between dispensations of the same OAT drug type exist

* Count the different relationships as defined by Allen left-side temporal relations within each OAT drug type subset (count_relations)

* Handle nested and overlapping dispensations of the same OAT type:

- If nested (contains, finishes, starts) then aggregate

- If overlap <= 7 days then shift */

%include "&SAS_path\05c_overlap_rx_macro.sas";

/* 1. Buprenorphine */

%let drug_grp = bup;

%***count_relations***(bup);

%***add_nested_rx***(bup);

%***shift_rx***(bup, **7**);

/* 2. iOAT */

%let drug_grp = ioat;

%***count_relations***(ioat);

%***add_nested_rx***(ioat);

%***shift_rx***(ioat, **7**);

/* 3. SROM */

%let drug_grp = srom;

%***count_relations***(srom);

%***add_nested_rx***(srom);

%***shift_rx***(srom, **7**);

/* 4. Methadone */

%let drug_grp = meth;

%***count_relations***(meth);

%***add_nested_rx***(meth);

%***shift_rx***(meth, **7**);

/* JOIN ALL OAT SUBGROUPS BACK TOGETHER

* final cleaned OAT PNET dataset */

**data** all_oat;

set shift_bup_out shift_ioat_out shift_srom_out shift_meth_out;

by moh_id date_of_service_shift end_date_service_shift;

rename date_of_service_shift=date_of_service end_date_service_shift=end_date_service days_supply_new=days_supply;

oat_num_drugs = countc(oat_drugs, '/') + **1**;

* round dose/day to one decimal;

dose_pday = round(dose_pday_sum, **0.1**);

* round oat_dose to one decimal;

oat_dose = round(oat_dose_sum, **0.1**);

drop date_of_service_new end_date_service_new interval_start interval_end dose_pday_sum oat_dose_sum;

retain row_id;

if first.moh_id then

row_id = **1**;

else row_id + **1**;

**run**;

/* A Methodological Framework for Constructing Opioid Agonist Therapy Episodes in Administrative Data: Insights and Key Challenges

* Researcher: Kiana Yazdani

* Analyst: Cassidy Tam

* Related to request 434: Impact of stimulant use disorder on outcomes related to opioid agonist therapy in people living with HIV

* June/03/2025 */

/* Continuous treatment for a particular OAT type is determined by the permissible gap

* Group together dispensations of the same OAT type within the permissible gap to simplify

identifying OAT co-prescriptions and transitions

* Permissible gaps:

1. Buprenorphine: 6 days

* Suboxone --> Sublocade: 42 days

- This is under the assumption that days supply of Sublocade is 1 day

- In PharmaNet should expect days supply to represent how long the drug lasts for

- Sublocade is prescribed 1/month, so days supply should also be around 1 month, so permissible

gap would still be 6 days

- Cases of Sublocade in this data (< 5) was prescribed on the same day as a Suboxone prescription

and then followed by another Suboxone prescription (each with one day supply),

so the cleaning process ended up changing the large days supply of Sublocade to one day.

Therefore we use a permissible gap of 42 days for Suboxone cases in this data.

2. iOAT: 3 days

3. SROM: 5 days

4. Methadone: 5 days */

/* Load derived episode of care (EOC) data using DAD hospitalization data accounting for hospital transfers */

**data** eoc8;

set der.eoc8_all;

keep moh_id eoc_id eoc_start eoc_end admit DIAG1 DIAGX1;

**run**;

**proc** **sort** data=eoc8;

by moh_id eoc_id eoc_start eoc_end admit;

**run**;

**proc** **contents** data=eoc8;

**run**;

/* Set EOC most responsible diagnosis (MRD) as MRD from first hospitalization record

* Prioritize diagx1 (ICD10) > diag1 (ICD9)

* Starting 2001/02 ICD9 codes are converted from ICD10 */

**data** hosp_tmp;

set eoc8;

by moh_id eoc_id;

length eoc_mrd $20.;

retain eoc_mrd;

if first.eoc_id then

do;

if diagx1 ne '' then

eoc_mrd = diagx1;

else eoc_mrd = diag1;

end;

**run**;

**data** hosp_out;

set hosp_tmp;

by moh_id eoc_id;

if last.eoc_id;

drop diag1 diagx1;

**run**;

/* Combine EOC data and cleaned OAT data

* Rename start and end date variables for usability */

**data** hosp_and_oat_cleaned;

retain moh_id oat_drug_name date_of_service days_supply dose_per_day;

set hosp_out (keep = moh_id eoc_start eoc_end eoc_mrd in = in_h rename = (eoc_start=addate eoc_end=sepdate))

all_oat (keep = moh_id date_of_service end_date_service days_supply oat_type oat_drug_gen oat_drugs dose_pday

rename = (date_of_service=addate end_date_service=sepdate));

* Set hospitalizations to have oat type = 0;

if in_h eq **1** then

oat_type = **0**;

where sepdate >= &study_start_dt. and addate <= &study_end_dt.;

**run**;

* Add row id;

**proc** **sort** data=hosp_and_oat_cleaned;

by moh_id addate sepdate;

**run**;

**data** hosp_and_oat_cleaned2;

retain row_id moh_id;

set hosp_and_oat_cleaned;

by moh_id;

retain row_id;

if first.moh_id then

row_id = **1**;

else row_id + **1**;

**run**;

* Load cohort;

**data** edar434_cohort;

set myref.edar434_cohort;

**run**;

/* Work with cohort group who had at least one OAT dispensation during study period

* Exclude dispensations initiated before the study period and continued or ended during the study

* Sort by OAT type for next step */

**proc** **sql**;

create table episode_tmp1a as

select a.moh_id, a.study_baseline, a.end_fu_dt,

b.days_supply, b.addate, b.sepdate, b.eoc_mrd, b.oat_type, b.oat_drug_gen, b.oat_drugs, b.dose_pday

from edar434_cohort a

inner join hosp_and_oat_cleaned b on a.moh_id = b.moh_id and a.study_baseline <= b.addate <= a.end_fu_dt

order by a.moh_id , b.oat_type, b.addate, b.sepdate

;

**quit**;

/* Group together continuous dispensations of the same OAT type where gap between dispensations is within permissible gap

* Flag first date where therapeutic dose was achieved: if OAT achieves therapeutic dose right after hospitalization discharge

OAT was most likely started during hospitalization so want episode to start at hospitalization

* Therapeutic dose/day:

1. Buprenorphine: 16 mg

2a. iOAT - Hydromorphone: 250 mg

2b. iOAT - Diacetylmorphine: 500 mg

3. SROM: 1200 mg

4. Methadone: 120 mg/ml */

**data** episode_tmp1b;

set episode_tmp1a;

by moh_id oat_type;

retain block_id block_start block_end block_type block_type_str block_ther_dose block_ther_dose_start_dt block_drugs;

format block_start block_end block_ther_dose_start_dt date9.;

length block_drugs $50.;

* Flag if therapeutic dose has been achieved;

if oat_type = **1** and dose_pday >= &bup_dose. then

ther_dose_flag = **1**;

else if oat_type = **2** and oat_drugs = 'iOAT_Hydr' and dose_pday >= &ioat_dose_hydro. then

ther_dose_flag = **1**;

else if oat_type = **2** and oat_drugs = 'iOAT_Diac' and dose_pday >= &ioat_dose_diac. then

ther_dose_flag = **1**;

else if oat_type = **3** and dose_pday >= &srom_dose. then

ther_dose_flag = **1**;

else if oat_type = **4** and dose_pday >= &meth_dose. then

ther_dose_flag = **1**;

else if oat_type = **0** then

ther_dose_flag = **7**;

* Check for errors;

else if oat_type = **.** then

ther_dose_flag = **9**;

* Therapeutic dose not achieved;

else ther_dose_flag = **0**;

* Reset for each participant;

if first.moh_id then

do;

new = **1**;

block_id = **0**;

end;

* Gap defined as difference between continuous interval so far and next dispensation (starting at addate);

gap = addate - block_end;

if first.moh_id then

gap = **.**;

* Build intervals of continuous dispensations of the same OAT type, defined by permissible gap;

if oat_type = block_type and block_type ne **0** then

do;

* Buprenorphine;

if oat_type = **1** then

do;

* Special case of Sublocade -> Suboxone;

if find(block_drugs, 'Sublocade', 'i') and find(oat_drugs, 'Suboxone', 'i') then

do;

oat_bup_switch = **1**;

flag = **1**;

if gap > &bup_sub_gap. then

new = **1**;

end;

else if gap > &bup_gap. then

do;

flag = **2**;

new = **1**;

end;

end;

* iOAT;

else if oat_type = **2** and gap > &ioat_gap. then

do;

flag = **2**;

new = **1**;

end;

* SROM;

else if oat_type = **3** and gap > &srom_gap. then

do;

flag = **2**;

new = **1**;

end;

* Methadone;

else if oat_type = **4** and gap > &meth_gap. then

do;

flag = **2**;

new = **1**;

end;

end;

* Otherwise if hospitalizations and not continuous then create a new block;

else if oat_type = block_type and block_type = **0** and gap > **1** then

new = **1**;

* Otherwise if different OAT type then create a new block;

else if oat_type ne block_type then

new = **1**;

* If new block update block info;

if new = **1** then

do;

block_start = addate;

block_end = sepdate;

block_type = oat_type;

block_type_str = oat_drug_gen;

block_ther_dose = ther_dose_flag;

block_id = block_id + **1**;

* therapeutic dose variables;

if block_ther_dose = **1** then

do;

block_ther_dose_start_dt = addate;

end;

else

do;

block_ther_dose_start_dt = **.**;

end;

end;

* Otherwise continuous block;

else

do;

* Update therapeutic dose variables;

if block_ther_dose = **0** and ther_dose_flag = **1** then

do;

block_ther_dose = **1**;

block_ther_dose_start_dt = addate;

end;

* Update end date of continuous block;

if sepdate > block_end then

block_end = sepdate;

end;

* Update drugs to check for Suboxone -> Sublocade transition;

block_drugs = oat_drugs;

**run**;

**proc** **sort** data=episode_tmp1b;

by moh_id block_id block_start block_end;

**run**;

* Take last row of each block built above;

**data** episode_tmp1c;

set episode_tmp1b;

by moh_id block_id;

if last.block_id;

drop addate sepdate ther_dose_flag oat_type oat_drug_gen oat_drugs dose_pday gap new eoc_mrd flag;

rename block_id = block_id_old;

**run**;

* Sort back to chronological order of block start and end dates;

**proc** **sort** data=episode_tmp1c out=episode_tmp1d;

by moh_id block_start block_end;

**run**;

/* A Methodological Framework for Constructing Opioid Agonist Therapy Episodes in Administrative Data: Insights and Key Challenges

* Researcher: Kiana Yazdani

* Analyst: Cassidy Tam

* Related to request 434: Impact of stimulant use disorder on outcomes related to opioid agonist therapy in people living with HIV

* June/03/2025 */

/* Incorporate hospitalizations in continuous OAT treatments

* If hospitalization is surrounded by blocks of the same OAT type then assume continuous treatment over hospitalizations

- OAT blocks can be fully nested in hospitalization blocks */

**%macro** hosp_oat_comb(oat, oat_num);

**%macro** ***colours***;

**%mend** colours;

/* Load OAT and hospitalizations blocks created in 06ai_oat_epi_gap code */

**data** &oat._hosp_tmp1;

set episode_tmp1d;

where block_type = &oat_num. or block_type = **0**;

drop end_fu_dt days_supply perm_gap_check;

**run**;

**proc** **sort** data=&oat._hosp_tmp1;

by moh_id block_start block_end;

**run**;

/* Carry forward OAT type over hospitalizations if gap between OAT and hospitalization is within the permissible gap of the OAT type

* Mark previous type that is within the permissible gap for possible continuous treatment

* Recall want: next start <= previous end + gap */

**data** &oat._hosp_tmp2;

retain moh_id row_id;

set &oat._hosp_tmp1;

by moh_id;

retain carry_type carry_end row_id;

format carry_end date9.;

row_id = ifn(first.moh_id, **1**, row_id + **1**);

gap = block_start - carry_end;

if first.moh_id then

do;

carry_type = block_type;

carry_end = block_end;

gap = **.**;

end;

/* Reset type and end date if permissible gap was exceeded or

* previous block was a hospitalization or previous block was another OAT block

* Only want to carry OAT types over hospitalizations */

else if gap > &&&oat**._**gap. or carry_type = **0** or block_type ne **0** then

do;

carry_type = block_type;

carry_end = block_end;

end;

/* Otherwise carry forward OAT type

* Update carry end to calculate gap with largest end date thus far

* Skip over multiple/fully nested hospitalizations */

else if block_end > carry_end then

do;

carry_end = block_end;

end;

/* Flag fully nested hospitalizations */

else if block_end <= carry_end then

nested_flag = **1**;

**run**;

/* Get type that follows hospitalizations

* Sort by descending chronological order of blocks

* Carry forward type over hospitalizations if gap between hospitalization and next OAT is within the permissible gap of the OAT type

* Mark type that is within the permissible gap for possible continuous treatment */

**proc** **sort** data=&oat._hosp_tmp2;

by moh_id descending row_id;

**run**;

**data** &oat._hosp_tmp3;

set &oat._hosp_tmp2;

by moh_id;

/* Keep track of next_end to flag fully nested hospitalizations */

retain next_type next_start next_end;

format next_start next_end date9.;

next_gap = next_start - block_end;

if first.moh_id then

do;

next_type = block_type;

next_start = block_start;

next_end = block_end;

next_gap = **.**;

end;

/* Reset type and end date if permissible gap exceeded or

* previous block was a hospitalization or previous block was another OAT block

* Only want to carry OATs over hospitalizations */

else if next_gap > &&&oat**._**gap. or next_type = **0** or block_type ne **0** then

do;

next_type = block_type;

next_start = block_start;

next_end = block_end;

end;

/* Otherwise carry forward OAT type

* Update next end to calculate gap with largest end date thus far

* Skip over multiple/fully nested hospitalizations */

else if block_start < next_start then

do;

next_start = block_start;

next_end = block_end;

end;

/* Flag fully nested hospitalizations */

else if next_start <= block_start and block_end <= next_end and nested_flag = **.** then

nested_flag = **2**;

**run**;

**proc** **sort** data=&oat._hosp_tmp3;

by moh_id row_id;

**run**;

/* If same OAT type before and after hospitalization then set hospitalization to that OAT type

* Recall carry_type and next_type indicate OAT type within permissible gap */

**data** &oat._hosp_tmp4;

set &oat._hosp_tmp3;

by moh_id;

/* Group in hospitalization with continuous OAT blocks */

if block_type = **0** and carry_type ne **0** and carry_type = next_type then

do;

group_flag = **1**;

block_type_new = carry_type;

end;

/* Group nested hospitalizations with previous OAT block */

else if block_type = **0** and nested_flag = **1** then

do;

block_type_new = carry_type;

group_flag = **2**;

end;

/* Group nested hospitalizations with next OAT block */

else if block_type = **0** and nested_flag = **2** then

do;

block_type_new = next_type;

group_flag = **3**;

end;

/* Otherwise keep blocks as is */

else block_type_new = block_type;

**run**;

/* Define new blocks of continuous OAT treatments over hospitalizations */

**data** &oat._hosp_tmp5;

set &oat._hosp_tmp4;

by moh_id;

retain block2_start block2_end block2_id block2_type block2_ther_dose block2_ther_dose_dt block2_flag;

format block2_start block2_end block2_ther_dose_dt date9.;

block2_gap = block_start - block2_end;

/* Reset for each participant */

if first.moh_id then

do;

block2_start = block_start;

block2_end = block_end;

block2_type = block_type_new;

block2_id = **1**;

block2_ther_dose = block_ther_dose;

block2_ther_dose_dt = block_ther_dose_start_dt;

block2_flag = group_flag;

end;

/* New block if different OAT type */

else if block2_type ne block_type_new then

new = **1**;

/* New block if permissible gap exceeded */

else if block2_type = &oat_num. and block2_gap > &&&oat**._**gap. then

new = **1**;

/* New block if isolated hospitalization */

else if block2_type ne &oat_num. then

new = **1**;

/* Reset if new block */

if new = **1** then

do;

block2_start = block_start;

block2_end = block_end;

block2_type = block_type_new;

block2_id = block2_id + **1**;

block2_ther_dose = block_ther_dose;

block2_ther_dose_dt = block_ther_dose_start_dt;

block2_flag = group_flag;

end;

/* Otherwise update end date to be latest date thus far

* Update if therapeutic dose achieved or not - if achieved ensure date is earliest date achieved

* group_flag indicates that hospitalization was grouped together with OAT treatment */

else if not first.moh_id then

do;

if block_end > block2_end then

block2_end = block_end;

if block2_ther_dose = **0** and block_ther_dose = **1** then

do;

block2_ther_dose = **1**;

block2_ther_dose_dt = block_ther_dose_start_dt;

end;

else if block2_ther_dose = **1** and block_ther_dose = **1**

and block2_ther_dose_dt > block_ther_dose_start_dt then

&oat._block_ther_dose_dt = block_ther_dose_start_dt;

if group_flag ne **.** then

block2_flag = group_flag;

end;

**run**;

/* Output last row within each block group */

**proc** **sort** data=&oat._hosp_tmp5;

by moh_id block2_id block2_start block2_end;

**run**;

**data** &oat._hosp_out;

set &oat._hosp_tmp5;

by moh_id block2_id;

if last.block2_id;

keep moh_id block2:;

drop block2_gap block2_id;

**run**;

/* Delete temporary data sets to save work space */

**proc** **datasets** lib=work nolist;

delete &oat._hosp_tmp:;

**quit**;

**%mend** hosp_oat_comb;

/* Run grouping hospitalization code above for each OAT type */

* Buprenorphine;

%***hosp_oat_comb***(bup, **1**);

* iOAT;

%***hosp_oat_comb***(ioat, **2**);

* SROM;

%***hosp_oat_comb***(srom, **3**);

* Methadone;

%***hosp_oat_comb***(meth, **4**);

/* Combine all new OAT blocks created above */

**data** hosp_oat_comb_tmp1;

set bup_hosp_out(where=(block2_type ne **0**))

ioat_hosp_out(where=(block2_type ne **0**))

srom_hosp_out(where=(block2_type ne **0**))

meth_hosp_out(where=(block2_type ne **0**));

by moh_id block2_start block2_end;

**run**;

/* Merge all isolated hospitalizations that were not grouped to any Buprenoprhine/iOAT/SROM/Methadone blocks

* Will exist as a separate row in each data set */

**data** hosp_oat_comb_tmp2;

merge bup_hosp_out(where=(block2_type = **0**) in=a)

ioat_hosp_out(where=(block2_type = **0**) in=b)

srom_hosp_out(where=(block2_type = **0**) in=c)

meth_hosp_out(where=(block2_type = **0**) in=d);

by moh_id block2_start block2_end;

if a and b and c and d then

output;

**run**;

/* Combine new OAT blocks and isolated hospitalizations */

**data** episode_tmp2;

set hosp_oat_comb_tmp1

hosp_oat_comb_tmp2;

by moh_id block2_start block2_end;

retain block2_id;

if first.moh_id then

block2_id = **1**;

else block2_id = block2_id + **1**;

block2_dur = block2_end - block2_start + **1**;

if block2_flag and block2_dur <= **14** then

block2_flag = **2**;

else if block2_flag = **.** then

block2_flag = **0**;

length block2_type_str $100.;

if block2_type = **0** then

block2_type_str = 'Hospital';

else if block2_type = **1** then

block2_type_str = 'Buprenorphine';

else if block2_type = **2** then

block2_type_str = 'Injective OAT';

else if block2_type = **3** then

block2_type_str = 'SROM';

else if block2_type = **4** then

block2_type_str = 'Methadone';

**run**;

/* A Methodological Framework for Constructing Opioid Agonist Therapy Episodes in Administrative Data: Insights and Key Challenges

* Researcher: Kiana Yazdani

* Analyst: Cassidy Tam

* Related to request 434: Impact of stimulant use disorder on outcomes related to opioid agonist therapy in people living with HIV

* June/03/2025 */

/* If overlap between DIFFERENT OAT types <= 14 days, then shift start date so there is no overlap

- Can change relationship with other OAT blocks

* Overlap relationship defined as: oat 1 start < oat 2 start < oat 1 end < oat 2 end

* Nested relationships (started by/contains/finished by) will be handled in the next step

* Same as shifting process in cleaning process - see shift_rx macro in 05c_overlap_rx_macro

- epsilon = 14 days

* check_flag flags the case of multiple OATs overlapping another

- In this case, OATs are shifted based on the dates of the OAT it overlaps

- Ignore the nested relationship

* Note only updating prev_start/prev_end with latest (maximum) dates to skip over nested records

- ie. (1) record 1

(2) record 2: fully nested with record 1 (record 1 start < record 2 start and record 2 end < record 1 end)

(3) record 3: after record 2 but overlaps record 1 (record 2 end < record 3 start < record 1 end) */

**proc** **sort** data=episode_tmp2 out=episode3_tmp1;

by moh_id block2_start block2_end;

**run**;

**data** episode3_tmp2;

set episode3_tmp1;

by moh_id;

/* Keep track of previous shifted start and end date

* Carry forward shifted end date where overlap exists */

retain prev_start prev_end prev_type carry_end carry_type;

format prev_start prev_end start_shift end_shift ther_shift carry_end date9.;

* Reset for each participant;

if first.moh_id then

do;

carry_end = **.**;

carry_type = **.**;

end;

/* Calculate number of days between current start date and previous shifted end date (not including start and end date)

* If there is overlap then number of days between start and end date < 0 (including start and end date) */

overlap_days = ifn(first.moh_id, **.**, block2_start - prev_end - **1**);

* Reset for each participant;

if first.moh_id then

start_shift = block2_start;

* Do not shift hospitalizations or OATs overlapping with hospitalizations;

else if prev_type = **0** or block2_type = **0** then

start_shift = block2_start;

* Reset if no overlap;

else if overlap_days >= **0** then

start_shift = block2_start;

* If overlap;

else if **.** < overlap_days < **0** then

do;

* Only shift if different OAT types;

if block2_type = prev_type then

do;

check_flag = **4**;

start_shift = block2_start;

end;

/* Odd case with multiple overlaps, and shifting causes current row to start before previous shifted row

* Shift current row to start at same time as previous row, similar to shifting relative to two previous records

* Removes any overlap <= 14 days, recall overap must have block 1 end < block 2 end */

else if block2_start < prev_start and block2_start <= carry_end then

do;

if carry_type = block2_type then

do;

check_flag = **5**;

start_shift = block2_start;

end;

else

do;

check_flag = **1**;

start_shift = prev_start;

end;

end;

* Nested relationship;

else if prev_start <= block2_start and block2_end <= prev_end then

do;

check_flag = **2**;

start_shift = block2_start;

end;

* Previous record is nested within current record;

else if block2_start <= prev_start and prev_end <= block2_end then

do;

check_flag = **3**;

start_shift = block2_start;

end;

* Standard overlapping relationship, shift;

else if prev_start < block2_start and prev_end < block2_end and overlap_days >= -**14** then

do;

start_shift = prev_end + **1**;

if prev_type = **4** and block2_type = **1** then

bup_micro_flag = **1**;

end;

* Co-prescription relationship, no shifting;

else if overlap_days < -**14** then

start_shift = block2_start;

* Catch errors;

else check_flag = **5**;

end;

* Update end date based on shifted date of service;

end_shift = start_shift + (block2_end - block2_start);

* Keep track of shifting;

shift_diff_start = abs(start_shift - block2_start);

shift_diff_end = abs(end_shift - block2_end);

* Update therapeutic dose date;

ther_shift = block2_ther_dose_dt + shift_diff_start;

* Carry forward start and end date for next row;

if first.moh_id then

do;

prev_start = start_shift;

prev_end = end_shift;

prev_type = block2_type;

end;

/* Do not update if record was nested

* Check overlap with latest end date

* Carry forward latest start date in the case that end_shift = prev_end

* Since ordered by chronological order */

else if end_shift >= prev_end then

do;

prev_start = start_shift;

prev_end = end_shift;

prev_type = block2_type;

end;

* If no overlap between shifted start date and previous shifted end date then reset;

if block2_start > carry_end then

do;

carry_end = end_shift;

carry_type = block2_type;

end;

**run**;

**proc** **freq** data=episode3_tmp2;

table check_flag;

**run**;

**proc** **tabulate** data=episode3_tmp2;

var shift_diff_start shift_diff_end;

table shift_diff_start shift_diff_end, n nmiss mean median mode min max Q1 Q3;

**run**;

* Drop old dates, and rename new dates for next step;

**proc** **sort** data=episode3_tmp2;

by moh_id start_shift end_shift;

**run**;

**data** episode3_tmp3;

set episode3_tmp2;

drop prev: carry_end shift_diff_start shift_diff_end block2_start block2_end block2_ther_dose_dt block2_id check_flag overlap_days;

rename start_shift=block2_start_new end_shift=block2_end_new ther_shift=block2_ther_dose_dt_new block2_dur=block2_dur_old;

block2_dur_new = end_shift - start_shift + **1**;

**run**;

* Create new block ID;

**data** episode3_tmp4;

retain moh_id block2_id;

set episode3_tmp3;

by moh_id;

retain block2_id;

if first.moh_id then

block2_id = **1**;

else block2_id = block2_id + **1**;

drop block2_dur_old;

rename block2_start_new=block2_start block2_end_new=block2_end block2_ther_dose_dt_new=block2_ther_dose_dt block2_dur_new=block2_dur;

**run**;

/* A Methodological Framework for Constructing Opioid Agonist Therapy Episodes in Administrative Data: Insights and Key Challenges

* Researcher: Kiana Yazdani

* Analyst: Cassidy Tam

* Related to request 434: Impact of stimulant use disorder on outcomes related to opioid agonist therapy in people living with HIV

* June/03/2025 */

/* Remove unstable periods

* If fully nested/starts/finishes relationship between blocks of different OAT types and period is <= 14 days then defined as unstable period

* If two OATs have same start and end date and <= 14 days, then remove both blocks completely

* Check for nested relationship between two blocks of the same OAT type (nested_flag = 5) */

* Sort by descending end date to find nested/starts/finishes relationships;

**proc** **sort** data=episode3_tmp4 out=episode4a_tmp1;

by moh_id block2_start descending block2_end;

**run**;

**data** episode4a_tmp2;

set episode4a_tmp1;

by moh_id;

retain nested_start nested_end nested_str nested_id;

format nested_start nested_end date9.;

length nested_str $20.;

/* Reset for each new participant */

if first.moh_id then

do;

nested_id = **1**;

nested_start = block2_start;

nested_end = block2_end;

nested_str = block2_type_str;

nested_flag = **.**;

end;

/* Fully nested unstable OAT block within another OAT block */

else if nested_start < block2_start and block2_end < nested_end and nested_str ne 'Hospital' and block2_type_str ne 'Hospital' and

block2_dur <= **14** then

do;

if find(block2_type_str, nested_str) then

nested_flag = **5**;

else nested_flag = **2**;

end;

/* Nested period <= 14 days, equals */

else if nested_str ne 'Hospital' and block2_type_str ne 'Hospital' and block2_dur <= **14** and

block2_start = nested_start and block2_end = nested_end then

do;

if find(block2_type_str, nested_str) then

nested_flag = **5**;

else nested_flag = **3**;

end;

/* Nested period <= 14 days, starts */

else if nested_str ne 'Hospital' and block2_type_str ne 'Hospital' and block2_dur <= **14** and

nested_start = block2_start and block2_end < nested_end then

do;

if find(block2_type_str, strip(nested_str)) then

nested_flag = **5**;

else nested_flag = **4**;

end;

/* Nested period <= 14 days, finishes */

else if nested_str ne 'Hospital' and block2_type_str ne 'Hospital' and block2_dur <= **14** and

nested_start < block2_start and block2_end = nested_end then

do;

if find(block2_type_str, strip(nested_str)) then

nested_flag = **5**;

else nested_flag = **4**;

end;

/* Reset once non-nested block

* Overlap relationship considered non-nested */

else if block2_end > nested_end then

do;

nested_id = nested_id + **1**;

nested_start = block2_start;

nested_end = block2_end;

nested_str = block2_type_str;

nested_flag = **.**;

end;

**run**;

**proc** **freq** data=episode4a_tmp2;

tables nested_flag;

**run**;

/* Mark equal blocks <= 14 days

* Mark Buprenorphine micro-dosing in the case that there was another OAT block nested within */

**proc** **sql**;

create table episode4a_tmp3(drop=nested_flag) as

select a.*, coalesce(a.nested_flag, b.nested_flag) as nested_flag_new, coalesce(a.bup_micro_flag, c.bup_micro_flag) as bup_micro_flag_new

from episode4a_tmp2 a

left join episode4a_tmp2 b on a.moh_id = b.moh_id and a.nested_id = b.nested_id and b.nested_flag = **3** and a.nested_flag = **.**

left join episode4a_tmp2 c on a.moh_id = c.moh_id and a.nested_id = c.nested_id and c.bup_micro_flag = **1** and a.bup_micro_flag = **.**

order by a.moh_id, a.block2_id;

**quit**;

/* Remove unstable periods */

**data** episode4a_tmp4;

set episode4a_tmp3;

by moh_id;

where nested_flag_new = **.** or nested_flag_new = **5**;

keep moh_id block2_start block2_end block2_type block2_type_str block2_dur block2_ther_dose block2_ther_dose_dt bup_micro_flag_new;

**run**;

**proc** **sort** data=episode4a_tmp4;

by moh_id block2_start block2_end;

**run**;

* Create new block ID;

**data** episode4a_tmp5;

retain moh_id block2_id;

set episode4a_tmp4;

by moh_id;

retain block2_id;

if first.moh_id then

block2_id = **1**;

else block2_id = block2_id + **1**;

**run**;

/* A Methodological Framework for Constructing Opioid Agonist Therapy Episodes in Administrative Data: Insights and Key Challenges

* Researcher: Kiana Yazdani

* Analyst: Cassidy Tam

* Related to request 434: Impact of stimulant use disorder on outcomes related to opioid agonist therapy in people living with HIV

* June/03/2025 */

/* Need to flag co-prescriptions in the case of hospitalization -> multiple OATs starting at therapeutic dose:

If Multiple OATs defined as a co-prescription (ie. duration > 14 days) and

at least one OAT started at therapeutic dose (therapeutic dose date MUST EQUAL start date)

then want to start co-prescription at the hospitalization when implementing transition rules in next step

* Flag where co-prescriptions start at therapeutic dose (co_flag)

* Note: the overlap at the start will be captured by the transition rule */

/* Sort by descending block3 end date to find where overlap occurs at the start of the block */

**proc** **sort** data=episode4a_tmp5 out=episode4b_tmp1;

by moh_id block2_start descending block2_end;

**run**;

**data** episode4b_tmp2;

set episode4b_tmp1;

by moh_id;

retain start_tmp end_tmp type_tmp num_rows ther_dose_flag;

length type_tmp $100.;

format start_tmp end_tmp date9.;

* Reset for each participant;

if first.moh_id then

do;

start_tmp = block2_start;

end_tmp = block2_end;

type_tmp = block2_type_str;

num_rows = **1**;

if block2_ther_dose_dt = block2_start then

ther_dose_flag = **1**;

else ther_dose_flag = **0**;

end;

/* If same start date (allow one day difference)

* Ignore one day dispensations since the one day allowance will miss-classify as overlapping */

else if **0** <= abs(block2_start - start_tmp) <= **1** and (end_tmp ne start_tmp) then

do;

num_rows = num_rows + **1**;

/* If co-prescription of OATs and at least one OAT started at therapeutic dose

* then flag to start co-presription episode at hospitalization */

if block2_dur > **14** and type_tmp ne 'Hospital' and block2_type_str ne 'Hospital' then

do;

type_tmp = catx(' + ', type_tmp, block2_type_str);

* Update therapeutic dose status if not already achieved;

if ther_dose_flag ne **1** and block2_ther_dose_dt = block2_start then

ther_dose_flag = **1**;

* If started at therapeutic dose then co-prescription would need to start at hospitalization;

if ther_dose_flag = **1** then

co_flag = **1**;

end;

* Catch errors;

else if block2_dur <= **14** and type_tmp ne 'Hospital' and block2_type_str ne 'Hospital' then

co_flag = **2**;

end;

* If new start date then reset;

else

do;

start_tmp = block2_start;

end_tmp = block2_end;

type_tmp = block2_type_str;

num_rows = **1**;

if block2_ther_dose_dt = block2_start then

ther_dose_flag = **1**;

else ther_dose_flag = **0**;

end;

**run**;

* Add co_flag to all corresponding blocks;

**proc** **sql**;

create table episode4b_tmp3 as

select a.moh_id, a.block2_id, a.block2_start, a.block2_end, a.block2_type_str, a.block2_ther_dose, a.block2_ther_dose_dt, a.block2_dur,

a.bup_micro_flag_new, a.co_flag, coalesce(a.co_flag, b.co_flag) as co_flag_new

from episode4b_tmp2 a

left join episode4b_tmp2 b on a.moh_id = b.moh_id and a.start_tmp = b.start_tmp and find(b.type_tmp, strip(a.type_tmp)) and b.co_flag = **1**

order by a.moh_id, a.block2_id

;

**quit**;

**data** episode4b_tmp4;

set episode4b_tmp3;

by moh_id;

drop co_flag;

**run**;

/* A Methodological Framework for Constructing Opioid Agonist Therapy Episodes in Administrative Data: Insights and Key Challenges

* Researcher: Kiana Yazdani

* Analyst: Cassidy Tam

* Related to request 434: Impact of stimulant use disorder on outcomes related to opioid agonist therapy in people living with HIV

* June/03/2025 */

/* Apply transition rules to determine start and end of an episode */

/* Create numeric block3_type variable */

**data** episode_count_tmp1;

set episode4b_tmp4;

* Create numeric type, to identify same type in cases of multiple OATs;

if find(block2_type_str, 'Buprenorphine') and find(block2_type_str, 'Injective OAT') and

find(block2_type_str, 'SROM') and find(block2_type_str, 'Methadone') then

block2_type_num = **15**;

else if find(block2_type_str, 'Buprenorphine') and find(block2_type_str, 'Injective OAT') and find(block2_type_str, 'SROM') then

block2_type_num = **11**;

else if find(block2_type_str, 'Buprenorphine') and find(block2_type_str, 'Injective OAT') and find(block2_type_str, 'Methadone') then

block2_type_num = **12**;

else if find(block2_type_str, 'Buprenorphine') and find(block2_type_str, 'SROM') and find(block2_type_str, 'Methadone') then

block2_type_num = **13**;

else if find(block2_type_str, 'Injective OAT') and find(block2_type_str, 'SROM') and find(block2_type_str, 'Methadone') then

block2_type_num = **14**;

else if find(block2_type_str, 'Buprenorphine') and find(block2_type_str, 'Injective OAT') then

block2_type_num = **5**;

else if find(block2_type_str, 'Buprenorphine') and find(block2_type_str, 'SROM') then

block2_type_num = **6**;

else if find(block2_type_str, 'Buprenorphine') and find(block2_type_str, 'Methadone') then

block2_type_num = **7**;

else if find(block2_type_str, 'Injective OAT') and find(block2_type_str, 'SROM') then

block2_type_num = **8**;

else if find(block2_type_str, 'Injective OAT') and find(block2_type_str, 'Methadone') then

block2_type_num = **9**;

else if find(block2_type_str, 'SROM') and find(block2_type_str, 'Methadone') then

block2_type_num = **10**;

else if strip(block2_type_str) = 'Methadone' then

block2_type_num = **4**;

else if strip(block2_type_str) = 'SROM' then

block2_type_num = **3**;

else if strip(block2_type_str) = 'Injective OAT' then

block2_type_num = **2**;

else if strip(block2_type_str) = 'Buprenorphine' then

block2_type_num = **1**;

else if block2_type_str = 'Hospital' then

block2_type_num = **0**;

rename block2_type_str = block2_type;

format block2_type_num oat_type_fmt.;

**run**;

**proc** **freq** data=episode_count_tmp1;

tables block2_type_num;

**run**;

/* Note so far, each row corresponds to a block of a single OAT type

* Carry forward co-prescriptions at the end of a block to properly classify the next transition

* new indicates the start of a new block

* flag describes the transition type:

0 = Not applicable

1 = Sublocade -> Suboxone

2 = Permissible gap exceeded

3 = > 2 OATs

4.1 = Hospitalization previously, but OAT did not start at therapeutic dose

4.2 = Hospitalization -> OAT 1 + OAT 2 co-prescription: co-prescription started at therapeutic dose, continuous block

4.3 = Continuous hospitalizations

4.4 = Block starts with hospitalization, since OAT starts at therapeutic dose

4.5 = OAT fully nested in hospitalization

5.1 = Continuous OAT 1 block

5.2 = Continuous OAT 1 block over hospitalization

5.3 = OAT 1 -> OAT 2, with co-prescription overlap

5.4 = OAT 1 w/ fully nested OAT 2 co-prescription

5.5 = OAT 1 -> OAT 2, with overlap <= 14 days: OAT transition

5.6 = OAT 1 -> OAT 2 continuous transition

5.7 = Methadone -> Buprenorphine: Low-dose induction

6.1 = Multiple OATs -> Single, different OAT types, non-continuous

6.2 = Multiple OATs -> Single, different OAT types, continuous

6.3 = Multiple OATs -> hospitalization, within permissible gap of OAT 1 and/or 2

6.4 = Multiple OATs -> hospitalization, > permissible gap of OAT 1 and 2

6.5 = Multiple OATs -> Single, same type, within permissible gap

9 = Start of new patient

10 = Check */

**data** episode_count_tmp2;

set episode_count_tmp1;

by moh_id;

* For checking purposes;

format prev_start prev_end date9.;

prev_type = ifn(first.moh_id, **.**, lag(block2_type_num));

prev_start = ifn(first.moh_id, **.**, lag(block2_start));

prev_end = ifn(first.moh_id, **.**, lag(block2_end));

* To determine if unstable period;

prev_dur = ifn(first.moh_id, **.**, lag(block2_dur));

* Carry forward OAT type, therapeutic dose, duration, end date of block thus far;

retain carry_type_num carry_end carry_type_str;

format carry_end date9. flag flag_fmt.;

length carry_type_str_new carry_type_str $100.;

gap = block2_start - carry_end;

* Reset for each participant;

if first.moh_id then

do;

gap = **.**;

flag = **9**;

new = **1**;

end;

* Flag > 2 OATs;

else if carry_type_num > **10** or block2_type_num > **10** then

flag = **3**;

/* Hospitalization -> OATs */

else if carry_type_num = **0** then

do;

* Hospitalization -> single OAT;

if **0** < block2_type_num <= **4** then

do;

* Check permissible gap;

if block2_type_num = **1** and gap > &bup_gap. then

do;

flag = **2**;

new = **1**;

end;

else if block2_type_num = **2** and gap > &ioat_gap. then

do;

flag = **2**;

new = **1**;

end;

else if block2_type_num = **3** and gap > &srom_gap. then

do;

flag = **2**;

new = **1**;

end;

else if block2_type_num = **4** and gap > &meth_gap. then

do;

flag = **2**;

new = **1**;

end;

* If fully nested then start block at hospitalization (continuous);

else if block2_end <= carry_end then

flag = **4.5**;

* If next block is a co-prescription that started at therapeutic dose then start block at hospitalization (continuous);

else if co_flag_new = **1** then

flag = **4.2**;

* Otherwise if next OAT does not start at therapeutic dose then do not include hospitalization in block;

else if block2_ther_dose = **0** or block2_ther_dose_dt ne block2_start then

do;

flag = **4.1**;

new = **1**;

end;

* If started at therapeutic dose then start block at hospitalization (continuous);

else if block2_ther_dose_dt = block2_start then

flag = **4.4**;

* Check flag;

else flag = **10**;

end;

* Hospitalization -> hospitalization: if any gap then new block;

else if block2_type_num = **0** and gap > **1** then

do;

flag = **2**;

new = **1**;

end;

* Consecutive hospitalizations;

else if block2_type_num = **0** and **0** <= gap <= **1** then

flag = **4.3**;

* Check flag;

else flag = **10**;

end;

/* 2. single OAT */

else if **0** < carry_type_num <= **4** then

do;

* OAT 1 -> OAT 1 or OAT 2 transition or OAT 1 -> hospitalization;

if **0** <= block2_type_num <= **4** then

do;

* If gap > permissible gap then new;

if carry_type_num = **1** and gap > &bup_gap. then

do;

flag = **2**;

new = **1**;

end;

else if carry_type_num = **2** and gap > &ioat_gap. then

do;

flag = **2**;

new = **1**;

end;

else if carry_type_num = **3** and gap > &srom_gap. then

do;

flag = **2**;

new = **1**;

end;

else if carry_type_num = **4** and gap > &meth_gap. then

do;

flag = **2**;

new = **1**;

end;

* Continuous episode (not yet grouped together due to shifting);

else if carry_type_num = block2_type_num then

flag = **5.1**;

* Single OAT type carried over hospitalization, continuous episode;

else if block2_type_num = **0** then

flag = **5.2**;

* OAT transition within permissible gap;

else if gap >= **1** and carry_type_num ne block2_type_num then

do;

if bup_micro_flag_new = **1** then

flag = **5.7**;

else flag = **5.6**;

new = **1**;

end;

* Otherwise OAT 1 -> OAT 2 with overlap;

else if gap < **1** and carry_type_num ne block2_type_num then

do;

/* Co-prescription (overlap > 14 days), continuous episode

* Note gap = start - prev_end

* If overlap, then total number of overlap days = start - prev_end - 1 */

if (gap - **1**) < -**14** then

do;

* Nested co-prescription, where end dates are more than one day apart;

if block2_end < carry_end - **1** then

flag = **5.4**;

else flag = **5.3**;

* Flag co-prescription types to carry over later;

block2_type_co_pres = catx(' + ', carry_type_str, block2_type);

end;

/* Overlap <= 14 days = not a co-prescription, OAT transition

* In the case that previous block was OAT continued over hospitalization */

else if (gap - **1**) >= -**14** then

do;

flag = **5.5**;

new = **1**;

end;

end;

* Check flag;

else flag = **10**;

end;

/* Check flag

* OAT 1 -> multiple OATs - can only transition FROM multiple OATs */

else flag = **10**;

end;

/* 3. multiple OATs: co-prescription

* Note overlap <= 14 handled above as OAT transitions */

else if carry_type_num > **4** then

do;

/* [OAT 1 + OAT 2] -> single OAT that is not OAT 1 or OAT 2 then new (non-continuous)

* No rules yet about what the permissible gap is for this transition

* In this data gaps >> all permissible gaps, so can just handle all non-continuous cases in the same way */

if **0** < block2_type_num <= **4** and find(carry_type_str, strip(block2_type)) = **0** then

do;

new = **1**;

if gap > **1** then

flag = **6.1**;

* Continuous (no gap);

else if gap = **1** then

flag = **6.2**;

* Catch errors;

else flag = **10**;

end;

* [OAT 1 + OAT 2] -> OAT 1 or OAT 2 then check permissible gap of OAT that continues on;

else if **0** < block2_type_num <= **4** and find(carry_type_str, strip(block2_type)) then

do;

/* Check permissible gap of OAT that continues

* e.g. [OAT 1 + OAT 2] -> OAT 1, new block if permissible gap of OAT 1 is exceeded */

check_flag = **3**;

if block2_type_num = **1** and gap > &bup_gap. then

do;

flag = **2**;

new = **1**;

end;

else if block2_type_num = **2** and gap > &ioat_gap. then

do;

flag = **2**;

new = **1**;

end;

else if block2_type_num = **3** and gap > &srom_gap. then

do;

flag = **2**;

new = **1**;

end;

else if block2_type_num = **4** and gap > &meth_gap. then

do;

flag = **2**;

new = **1**;

end;

* Continuous block;

else flag = **6.5**;

end;

/* [OAT 1 + OAT 2] -> hospitalization

* Carry forward OATs that are within permissible gap */

else if block2_type_num = **0** then

do;

carry_type_str_new = '';

if find(carry_type_str, "Buprenorphine") and gap <= &bup_gap. then

do;

flag = **6.3**;

carry_type_str_new = catx(' + ', carry_type_str_new, "Buprenorphine");

end;

if find(carry_type_str, "Injective OAT") and gap <= &ioat_gap. then

do;

flag = **6.3**;

carry_type_str_new = catx(' + ', carry_type_str_new, "Injective OAT");

end;

if find(carry_type_str, "SROM") and gap <= &srom_gap. then

do;

flag = **6.3**;

carry_type_str_new = catx(' + ', carry_type_str_new, "SROM");

end;

if find(carry_type_str, "Methadone") and gap <= &meth_gap. then

do;

flag = **6.3**;

carry_type_str_new = catx(' + ', carry_type_str_new, "Methadone");

end;

/* If carry_type_str_new was not populated then hospitalization

* not within permissible gap of OAT 1 or OAT 2 => new block */

if carry_type_str_new = '' then

do;

new = **1**;

flag = **6.4**;

end;

* Otherwise update carry_type_str to OATs that followed within permissible gap;

else carry_type_str = carry_type_str_new;

end;

* Check flag;

else flag = **10**;

end;

* Catch errors;

else flag = **10**;

/* Reset if new block defined by transition rules above

* Reset values to carry forward for next transition */

if new = **1** then

do;

carry_end = block2_end;

carry_type_str = strip(block2_type);

carry_type_num = block2_type_num;

end;

* Otherwise continuous block;

else

do;

/* Update OAT type to carry forward if new OAT block ends after block thus far

* block2 end needs to end more than one day after end date thus far */

if block2_end > carry_end + **1** and block2_type_num ne **0** then

do;

carry_type_num = block2_type_num;

carry_type_str = block2_type;

end;

* If OAT fully nested in hospitalization then carry forward OAT type;

else if flag = **4.5** then

do;

carry_type_str = block2_type;

carry_type_num = block2_type_num;

end;

/* If block2 ends within one day of episode end date (thus far)

* Update OAT type to carry forward if not hospitalization

* If transition to OAT (with no co-prescription overlap) then only carry forward block2 type

* If co-prescription then carry forward both OAT types */

else if **0** <= abs(block2_end - carry_end) <= **1** and block2_type_num ne **0** then

do;

check_flag = **1**;

* Not co-prescription;

if flag ne **5.3** then

do;

check_flag = **2**;

carry_type_str = block2_type;

carry_type_num = block2_type_num;

end;

* Ending on co-prescription overlap, concatenate types;

else if find(carry_type_str, block2_type) = **0** then

carry_type_str = catx(' + ', carry_type_str, block2_type);

end;

* Update episode end date and episode duration thus far (now including current block2);

if block2_end > carry_end then

carry_end = block2_end;

end;

* Update numeric carry type variable if multiple OATs;

if find(carry_type_str, 'Buprenorphine') and find(carry_type_str, 'Injective OAT') and

find(carry_type_str, 'SROM') and find(carry_type_str, 'Methadone') then

carry_type_num = **15**;

else if find(carry_type_str, 'Buprenorphine') and find(carry_type_str, 'Injective OAT') and find(carry_type_str, 'SROM') then

carry_type_num = **11**;

else if find(carry_type_str, 'Buprenorphine') and find(carry_type_str, 'Injective OAT') and find(carry_type_str, 'Methadone') then

carry_type_num = **12**;

else if find(carry_type_str, 'Buprenorphine') and find(carry_type_str, 'SROM') and find(carry_type_str, 'Methadone') then

carry_type_num = **13**;

else if find(carry_type_str, 'Injective OAT') and find(carry_type_str, 'SROM') and find(carry_type_str, 'Methadone') then

carry_type_num = **14**;

else if find(carry_type_str, 'Buprenorphine') and find(carry_type_str, 'Injective OAT') then

carry_type_num = **5**;

else if find(carry_type_str, 'Buprenorphine') and find(carry_type_str, 'SROM') then

carry_type_num = **6**;

else if find(carry_type_str, 'Buprenorphine') and find(carry_type_str, 'Methadone') then

carry_type_num = **7**;

else if find(carry_type_str, 'Injective OAT') and find(carry_type_str, 'SROM') then

carry_type_num = **8**;

else if find(carry_type_str, 'Injective OAT') and find(carry_type_str, 'Methadone') then

carry_type_num = **9**;

else if find(carry_type_str, 'SROM') and find(carry_type_str, 'Methadone') then

carry_type_num = **10**;

* Numeric variable of co-prescription;

if find(block2_type_co_pres, 'Buprenorphine') and find(block2_type_co_pres, 'Injective OAT') and

find(block2_type_co_pres, 'SROM') and find(block2_type_co_pres, 'Methadone') then

block2_type_co_pres_num = **15**;

else if find(block2_type_co_pres, 'Buprenorphine') and find(block2_type_co_pres, 'Injective OAT') and find(block2_type_co_pres, 'SROM') then

block2_type_co_pres_num = **11**;

else if find(block2_type_co_pres, 'Buprenorphine') and find(block2_type_co_pres, 'Injective OAT') and find(block2_type_co_pres, 'Methadone') then

block2_type_co_pres_num = **12**;

else if find(block2_type_co_pres, 'Buprenorphine') and find(block2_type_co_pres, 'SROM') and find(block2_type_co_pres, 'Methadone') then

block2_type_co_pres_num = **13**;

else if find(block2_type_co_pres, 'Injective OAT') and find(block2_type_co_pres, 'SROM') and find(block2_type_co_pres, 'Methadone') then

block2_type_co_pres_num = **14**;

else if find(block2_type_co_pres, 'Buprenorphine') and find(block2_type_co_pres, 'Injective OAT') then

block2_type_co_pres_num = **5**;

else if find(block2_type_co_pres, 'Buprenorphine') and find(block2_type_co_pres, 'SROM') then

block2_type_co_pres_num = **6**;

else if find(block2_type_co_pres, 'Buprenorphine') and find(block2_type_co_pres, 'Methadone') then

block2_type_co_pres_num = **7**;

else if find(block2_type_co_pres, 'Injective OAT') and find(block2_type_co_pres, 'SROM') then

block2_type_co_pres_num = **8**;

else if find(block2_type_co_pres, 'Injective OAT') and find(block2_type_co_pres, 'Methadone') then

block2_type_co_pres_num = **9**;

else if find(block2_type_co_pres, 'SROM') and find(block2_type_co_pres, 'Methadone') then

block2_type_co_pres_num = **10**;

**run**;

**proc** **freq** data=episode_count_tmp2;

tables flag / nocum;

format flag flag_fmt.;

**run**;

/* Check if hospitalization should be included in OAT episode */

**data** episode_count_tmp3;

merge episode_count_tmp2

episode_count_tmp2(firstobs=**2** keep=moh_id block2_start flag new block2_type block2_type_num block2_ther_dose_dt

rename=(moh_id=pop_id2 block2_start=next_start flag=next_flag new=next_new block2_type=next_type

block2_type_num=next_type_num block2_ther_dose_dt=next_ther_dose_dt));

if moh_id ne pop_id2 then

do;

call missing(next_start);

call missing(next_flag);

call missing(next_new);

call missing(next_type_num);

call missing(next_type);

call missing(next_ther_dose_dt);

end;

**run**;

* Remove stand alone hospitalizations;

**data** episode_count_tmp3b;

set episode_count_tmp3;

by moh_id;

* New episode starts with hospitalization and next record is start of new episode, or no records follow then single hospitalization;

if block2_type_num = **0** and new = **1** and (next_new = **1** or next_start = **.**) then

delete_flag = **1**;

prev_new = ifn(first.moh_id, **.**, lag(new));

prev_flag = ifn(first.moh_id, **.**, lag(flag));

prev_gap = ifn(first.moh_id, **.**, intck('day', lag(block2_end), block2_start) - **1**);

format prev_flag flag_fmt.;

**run**;

**data** episode_count_tmp3c;

set episode_count_tmp3b;

by moh_id;

where delete_flag ne **1**;

keep moh_id block2: new flag carry: bup_micro_flag_new;

**run**;

/* A Methodological Framework for Constructing Opioid Agonist Therapy Episodes in Administrative Data: Insights and Key Challenges

* Researcher: Kiana Yazdani

* Analyst: Cassidy Tam

* Related to request 434: Impact of stimulant use disorder on outcomes related to opioid agonist therapy in people living with HIV

* June/03/2025 */

/* Define episodes

* Overall OAT episode = continuous OAT treatment: defined by permissible gap

* Episodes are defined by the transition rules as seen in 06ci_oat_epi_transitions

* Keep OAT types in co-prescriptions as distinct blocks */

* Join back study baseline and end of follow up to ensure episode is constrained within study;

**proc** **sql**;

create table episode_count_tmp4a as

select a.*, b.study_baseline, b.end_fu_dt

from episode_count_tmp3c a

left join edar434_cohort b on a.moh_id = b.moh_id

order by a.moh_id, a.block2_id, a.block2_start, a.block2_end;

**quit**;

/* Define start and end of episode based on transition rules */

**data** episode_count_tmp4b;

set episode_count_tmp4a;

by moh_id;

retain epi_start epi_end epi_id epi_oat_str epi_ther_dose_dt epi_type;

format epi_start epi_end epi_ther_dose_dt date9. epi_type epi_type_fmt.;

length epi_oat_str $50.;

if first.moh_id then

do;

epi_start = block2_start;

epi_end = min(block2_end, end_fu_dt);

epi_id = **1**;

if block2_type_num ne **0** then

epi_oat_str = block2_type;

else epi_oat_str = '';

epi_ther_dose_dt = block2_ther_dose_dt;

* Assume monotherapy;

epi_type = **1**;

end;

/* New episode determined by permissible gap (flag = 2, 6.4)

* or if previous block was isolated hospitalization (flag = 4.1) */

else if flag = **2** or flag = **4.1** or flag = **6.4** then

do;

epi_id = epi_id + **1**;

if block2_type_num ne **0** then

epi_oat_str = block2_type;

else epi_oat_str = '';

epi_ther_dose_dt = block2_ther_dose_dt;

epi_start = block2_start;

epi_end = min(block2_end, end_fu_dt);

* Assume monotherapy;

epi_type = **1**;

end;

/* Multiple OAT -> Single OAT if gap > 0 then consider new episode

* No guidelines for this case in transition rules

* In this data, all cases where a gap exists, gap >> permissible gap of any OAT */

else if flag = **6.1** then

do;

epi_id = epi_id + **1**;

if block2_type_num ne **0** then

epi_oat_str = block2_type;

else epi_oat_str = '';

epi_ther_dose_dt = block2_ther_dose_dt;

epi_start = block2_start;

epi_end = min(block2_end, end_fu_dt);

* Assume monotherapy;

epi_type = **1**;

end;

* Otherwise continuous episode;

else

do;

/* Update episode type */

* Co-prescription;

if flag in (**5.3**, **5.4**, **4.2**) then

do;

* Combination therapy;

if epi_type = **1** then

epi_type = **3**;

* Transition and combination therapy;

else if epi_type = **2** then

epi_type = **4**;

end;

* Transition;

else if flag in (**5.5**, **5.6**, **5.7**) then

do;

if epi_type = **1** then

epi_type = **2**;

* Transition and combination therapy;

else if epi_type = **3** then

epi_type = **4**;

end;

* Both transition and combination therapy;

else if flag in (**6.2**, **6.5**) then

epi_type = **4**;

* Update end date;

if epi_end < block2_end <= end_fu_dt then

epi_end = block2_end;

else if block2_end > end_fu_dt then

epi_end = end_fu_dt;

* Update therapeutic dose;

if epi_ther_dose_dt = **.** and block2_ther_dose_dt ne **.** then

epi_ther_dose_dt = block2_ther_dose_dt;

else if epi_ther_dose_dt > block2_ther_dose_dt > **.** then

do;

epi_ther_dose_check = **1**;

epi_ther_dose_dt = block2_ther_dose_dt;

end;

* Want earliest therapeutic dose date achieved;

else if block2_ther_dose_dt > epi_ther_dose_dt > **.** then

epi_ther_dose_check = **2**;

if find(epi_oat_str, strip(block2_type)) = **0** and block2_type_num ne **0** then

epi_oat_str = catx('/', epi_oat_str, block2_type);

end;

**run**;

* Keep variables of interest;

**PROC** **SQL**;

CREATE TABLE episode_count_tmp4c AS

SELECT t1.moh_id,

t1.study_baseline,

t1.end_fu_dt,

t1.epi_id,

t1.epi_start,

t1.epi_end,

t1.epi_type,

t1.epi_oat_str,

t1.epi_ther_dose_dt,

t1.block2_id,

t1.block2_start,

t1.block2_end,

t1.block2_type,

t1.block2_type_num,

t1.block2_ther_dose_dt,

t1.flag,

t1.carry_type_str,

t1.carry_type_num

FROM WORK.EPISODE_COUNT_TMP4B t1;

**QUIT**;

/* Add largest permissible gap to end of episode by checking in order of descending permissible gap size */

**proc** **sort** data=episode_count_tmp4c;

by moh_id epi_id;

**run**;

**data** episode_count_tmp4d;

set episode_count_tmp4c;

by moh_id epi_id;

format epi_end_new date9.;

if find(carry_type_str, "Buprenorphine", 'i') then

epi_end_new = min(end_fu_dt, epi_end + &bup_gap.);

else if find(carry_type_str, "SROM", 'i') then

epi_end_new = min(end_fu_dt, epi_end + &srom_gap.);

else if find(carry_type_str, "Methadone", 'i') then

epi_end_new = min(end_fu_dt, epi_end + &meth_gap.);

else if find(carry_type_str, "Injective OAT", 'i') then

epi_end_new = min(end_fu_dt, epi_end + &ioat_gap.);

if last.epi_id then

output;

drop block2:;

**run**;

**proc** **freq** data=episode_count_tmp4d;

tables epi_type;

**run**;

/* Check gap between episodes

* Recall permissible gap was added to the end */

**data** episode_count_check;

set episode_count_tmp4d;

by moh_id;

prev_end = ifn(first.moh_id, **.**, lag(epi_end));

format prev_end date9.;

gap = ifn(first.moh_id, **.**, epi_start - prev_end);

prev_carry = ifc(first.moh_id, '', lag(carry_type_str));

**run**;

/* Final episodes */

**data** episode_count_out;

retain moh_id study_baseline end_fu_dt epi_id epi_type epi_oat_str

epi_start epi_end_new epi_dur epi_discon epi_end epi_per_dur epi_induc epi_ther_dose_dt epi_induc_dur

epi_stabl_dt epi_stabl_dur;

set episode_count_tmp4d;

by moh_id;

format epi_stabl_dt date9.;

* New duration: adding permissible gap to end of episode;

epi_dur = epi_end_new - epi_start + **1**;

* Induction (yes/no);

if epi_ther_dose_dt ne **.** then

epi_induc = **1**;

else epi_induc = **0**;

* Induction duration;

if epi_ther_dose_dt ne **.** then

epi_induc_dur = epi_ther_dose_dt - epi_start + **1**;

else epi_induc_dur = **.**;

* Stabilization duration;

if epi_induc ne **0** then

epi_stabl_dur = epi_end_new - epi_ther_dose_dt;

else epi_stabl_dur = **.**;

* Stabilization date;

if epi_ther_dose_dt = epi_start then

epi_stabl_dt = epi_start;

else if epi_ther_dose_dt ne **.** and epi_ther_dose_dt ne epi_end_new then

epi_stabl_dt = epi_ther_dose_dt + **1**;

else epi_stabl_dt = **.**;

* Treatment discontinuation: if discontinued;

if epi_end_new ne end_fu_dt then

epi_discon = **1**;

else epi_discon = **0**;

* Persistence = start date to last dose (end date);

epi_per_dur = epi_end - epi_start + **1**;

label epi_id = 'Episode ID'

epi_start = 'Episode start date'

epi_end_new = 'Episode end date'

epi_type = 'Episode treatment type'

epi_oat_str = 'List of distinct OATs per episode'

epi_ther_dose_dt = 'First date therapeutic dose achieved'

epi_dur = 'Episode duration'

epi_discon = 'If episode discontinued'

epi_end = 'Last dose date for episode'

epi_induc = 'If induction completed'

epi_induc_dur = 'Induction duration'

epi_stabl_dur = 'Stabilization duration'

epi_stabl_dt = 'Stabilization start date'

epi_per_dur = 'Persistence duration'

;

drop flag carry_type_str carry_type_num;

rename epi_end=epi_last_dose_dt epi_end_new=epi_end;

**run**;
